# Author Correction: High expression of *Rex-orf-I* and *HBZ* mRNAs and bronchiectasis in lung of HTLV-1A/C infected macaques

**DOI:** 10.1038/s41467-026-77329-y

**Published:** 2026-09-03

**Authors:** Sarkis Sarkis, Anna Gutowska, Mohammad Arif Rahman, Luca Schifanella, Katherine C. Goldfarbmuren, Massimiliano Bissa, Ramona Moles, Christina Ramirez, Elijah F. Edmondson, Andrew Warner, Melvin Doster, Isabela Silva de Castro, Robyn Washington-Parks, Sophia Brown, Joshua Kramer, Matthew W. Breed, Kristin E. Killoran, Yogita Jethmalani, Leonid Serebryannyy, Damian FJ. Purcell, Cynthia A. Pise-Masison, Genoveffa Franchini

**Affiliations:** 1 Animal Models and Retroviral Vaccines Section, Basic Research Laboratory, Center for Cancer Research, National Cancer Institute, Bethesda, MD USA; 2 Advanced Biomedical Computational Science, Frederick National Laboratory for Cancer Research, Frederick, MD USA; 3 Center for Cancer Research Collaborative Bioinformatics Resource, National Cancer Institute, Bethesda, MD USA; 4 Department of Cell and Molecular Biology, Center for Immunology and Microbial Research, Cancer Center and Research Institute, University of Mississippi Medical Center, Jackson, MS USA; 5 Molecular Histopathology Laboratory, Frederick National Laboratory for Cancer Research, Frederick, MD USA; 6 Laboratory Animal Sciences Program, Frederick National Laboratory for Cancer Research, Frederick, MD USA; 7 Clemson University, Clemson, SC USA; 8 Vaccine Research Center, National Institute of Allergy and Infectious Diseases, National Institutes of Health, Bethesda, MD USA; 9 Department of Microbiology and Immunology, The Peter Doherty Institute for Infection and Immunity, The University of Melbourne, Parkville, VIC Australia; 10 Office of Scientific Programs, Center for Cancer Research, National Cancer Institute, Bethesda, MD USA

**Keywords:** Virus-host interactions, Mechanisms of disease, Viral infection, Viral host response

Correction to: *Nature Communications* https://doi.org/10.1038/s41467-025-63325-1, published online 26 September 2025

In the version of this article initially published, there were data-entry errors in the Source Data for Fig. 3 and Supplementary Figs. 6, 7 for week 21 bronchoalveolar lavage (BAL) neutrophil-frequency values for animal DHF6. Following correction of the Source Data and repeated analysis, one additional comparison became statistically significant: at week 21, the frequency of BAL CD162^+^IL-10^+^ neutrophils is significantly higher in HTLV-1A-infected animals than in HTLV-1A/C_*oI-L*_-infected animals. In the main text, “In concert, both IL-10^+^ neutrophils and the CD162^+^IL-10^+^ neutrophil subset were present at lower frequencies in HTLV-1A/C_*oI-L*_ infection (Fig. 3c)” now replaces the original “In concert, IL-10 producing neutrophils had lower frequencies in HTLV-1A/C_*oI-L*_ infection (Fig. 3c).” The text, Fig. 3c, Supplementary Figs. 6, 7 and associated Source Data have been updated in the HTML and PDF versions of the article. For comparison, the original figures are available as Supplementary Information accompanying this article.

## Supplementary information

Original Fig. 3, Supplementary Figs. 6, 7

