# Supplementary material for: High expression of Rex-orf-I and HBZ mRNAs and bronchiectasis in lung of HTLV-1A/C infected macaques
Source: Nat Commun. 2025 Sep 26;16:8470. doi: 10.1038/s41467-025-63325-1 (PMC12474910; doi:10.1038/s41467-025-63325-1)

Supplementary Figure 1

a

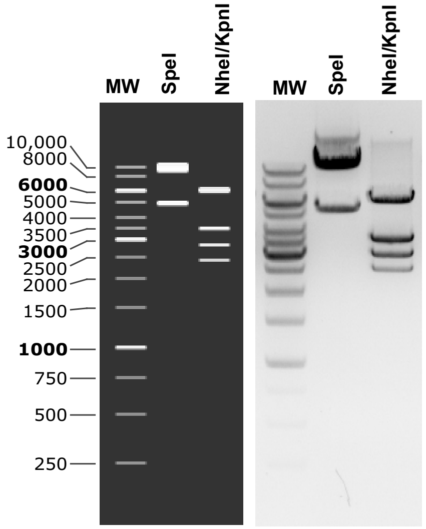

b

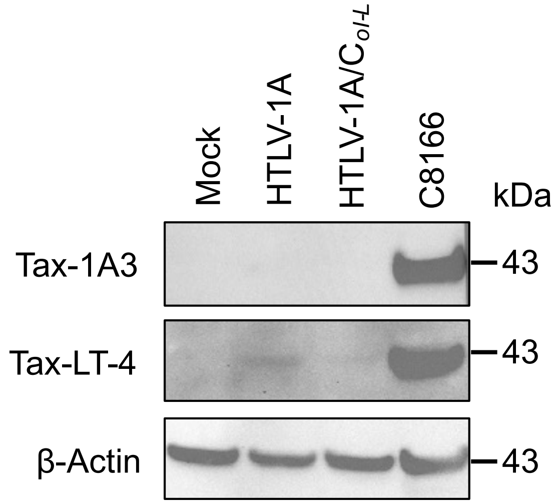

c

```
                           Nuclear Localization Signal        CREB-1 activation region
HTLV-1A-Tax      MAHFPGFGQSLLFGYPVYVFGDCVQGDWCPISGGLCSARLHRHALLATCPEHQITWDPID   60
HTLV-1C-Tax      MAHFPGFGQSLLYGYPVYVYGDCVQGDWCPISGGLCSARLHRHALLATCPEHQITWDPID   60
                 ************:******:****************************************

                                      CBP/P300         IKK Binding domain   LZD-1
HTLV-1A-Tax      GRVIGSALQFLIPRLPSFPTQRTSKTLKVLTPPITHTTPNIPPSFLQAMRKYSPFRNGYM   120
HTLV-1C-Tax      GRVIGSALQFLIPRLPSFPTQRTPKTLKVLTPPTTHTTPNIPPSFFQAIRKHSPFRNGYM   120
                 *********************** ********** **********:**:**:********

                                        NF-κB activation region
HTLV-1A-Tax      EPTLGQHLPTLSFPDPGLRPQNLYTLWGGSVVCMYLYQLSPPITWPLLPHVIFDHPGQLG   180
HTLV-1C-Tax      EPTLGQQLPSLSFPDPGLRPQNLYTLWGSSVVCMYLYQLSPPITWPLLPQVIFCHPGQLG   180
                 ******:**:******************.*********************:*** *****

                      Nuclear Export Signal                        LZD-2
HTLV-1A-Tax      AFLTNVPYKRIEELLYKISLTTGALIILPEDCLPTTLFQPVRAPVTLTAWQNGLLPFHST   240
HTLV-1C-Tax      AFLTNVPYKRMEELLYKIFLNTGALIILPEGCLPTTLFQPIRAPATLTAWQNGLLPFQST   240
                 **********:******* *.**********.*********:***.***********:**

HTLV-1A-Tax      LTTPGLIWTFTDGTPMISGPCPKDGQPSLVLQSSSFIFHKFQTKAYHPSFLLSHGLIQYS   300
HTLV-1C-Tax      LTTPGLIWTFSDGTPMISGPCPKDGQPSLVLQSSSFIFHKFQTKAYHPSVLLSHGLIQYS   300
                 **********:**************************************.**********

                  ATF/CREB activation domain                         PDZ Binding
HTLV-1A-Tax      SFHNLHLLFEEYTNIPISLLFNEKEADDNDHEPQISPGGLEPPSEKHFRETEV* 353
HTLV-1C-Tax      SFHSLHLLFEEYTNIPISLLFNEKEADDTDHGPQIPSEGLEPPSEKHFHETEV* 353
                 ***.************************.** ***   ***********:****
```

Supplementary Figure 2

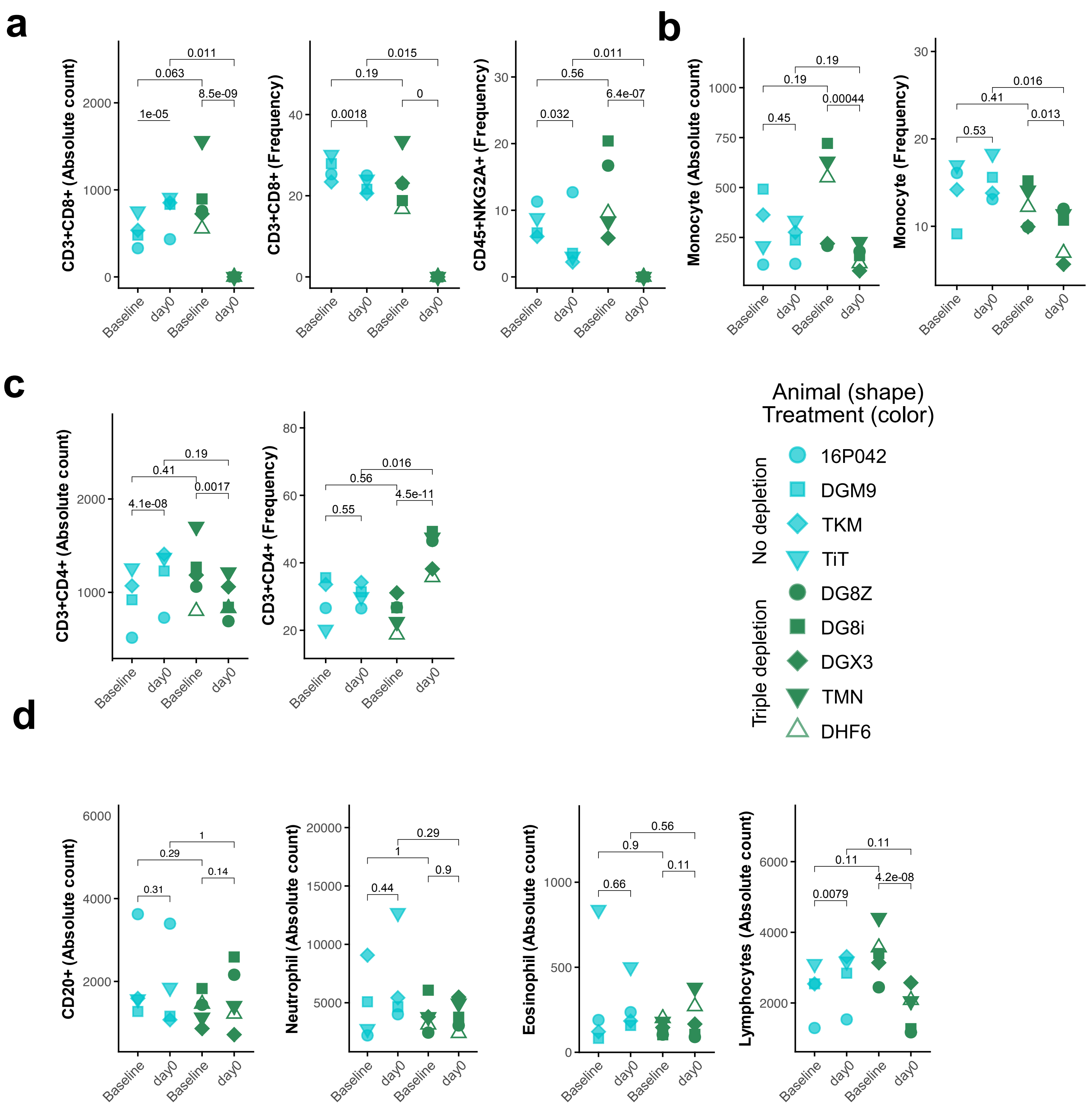

Supplementary Figure 3

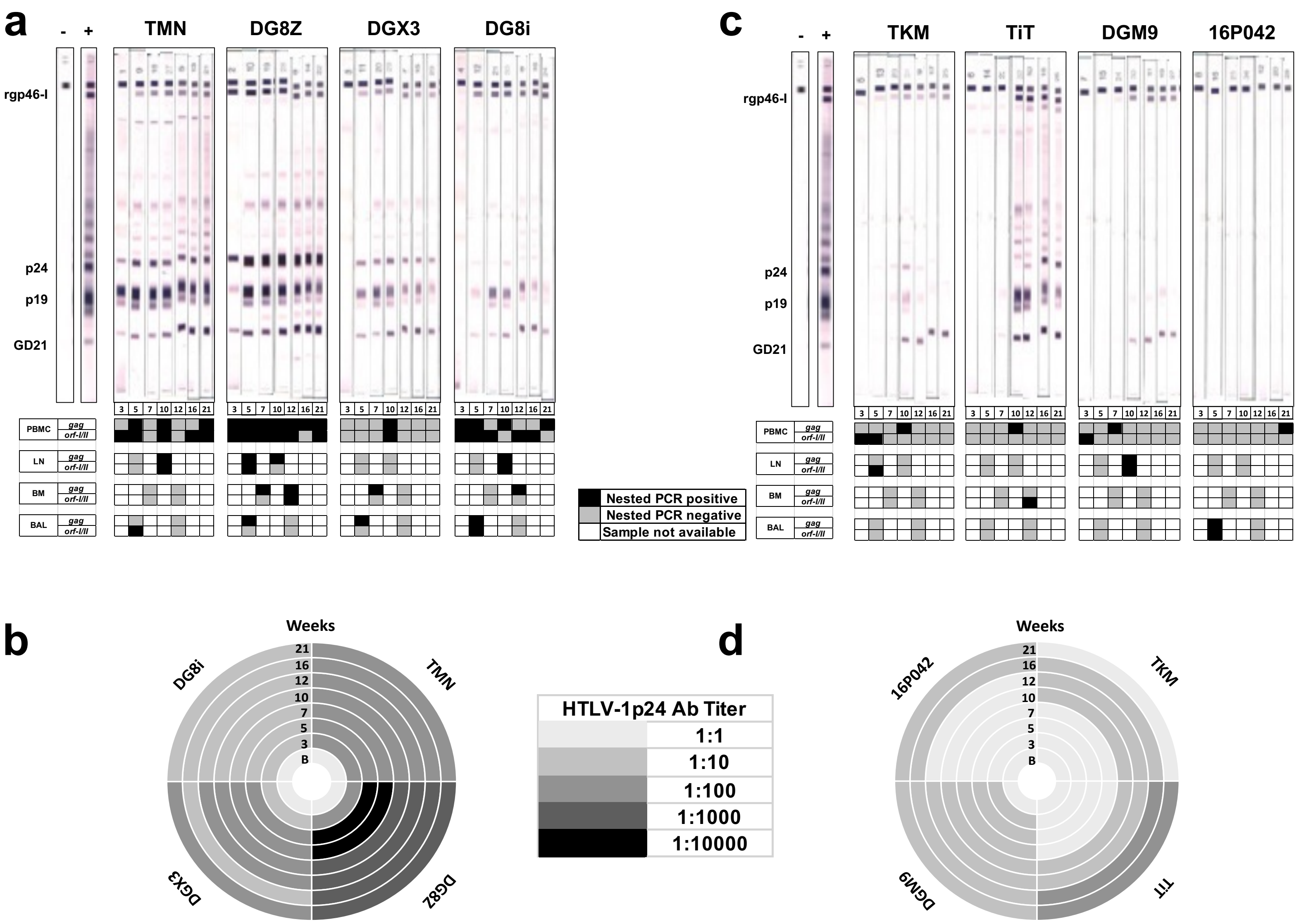

Supplementary Figure 3

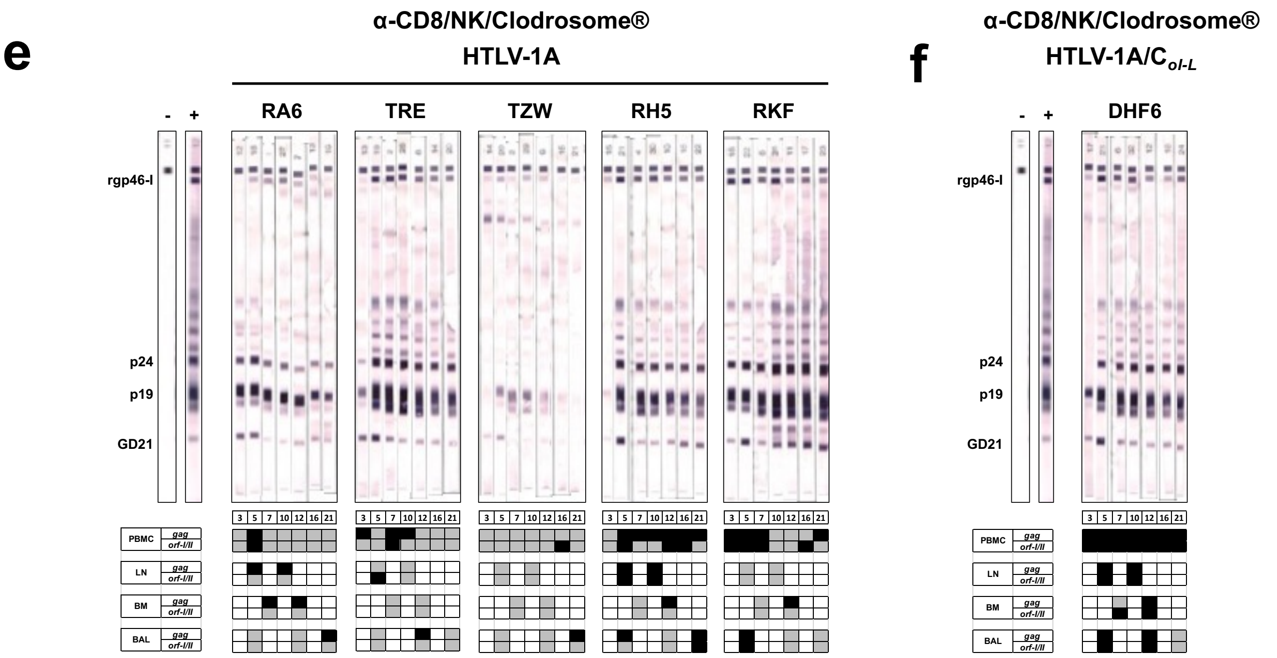

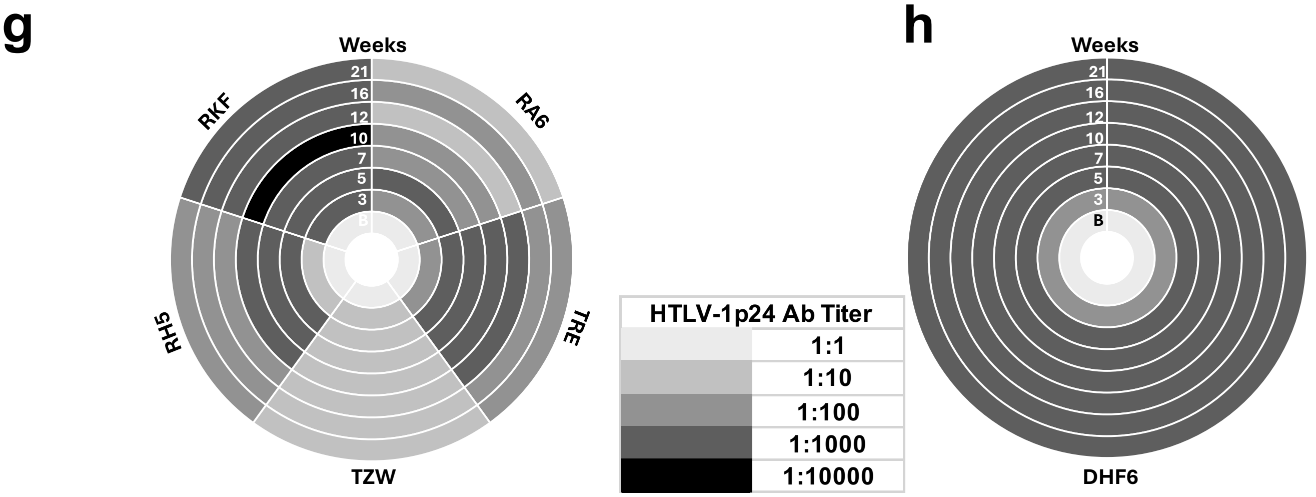

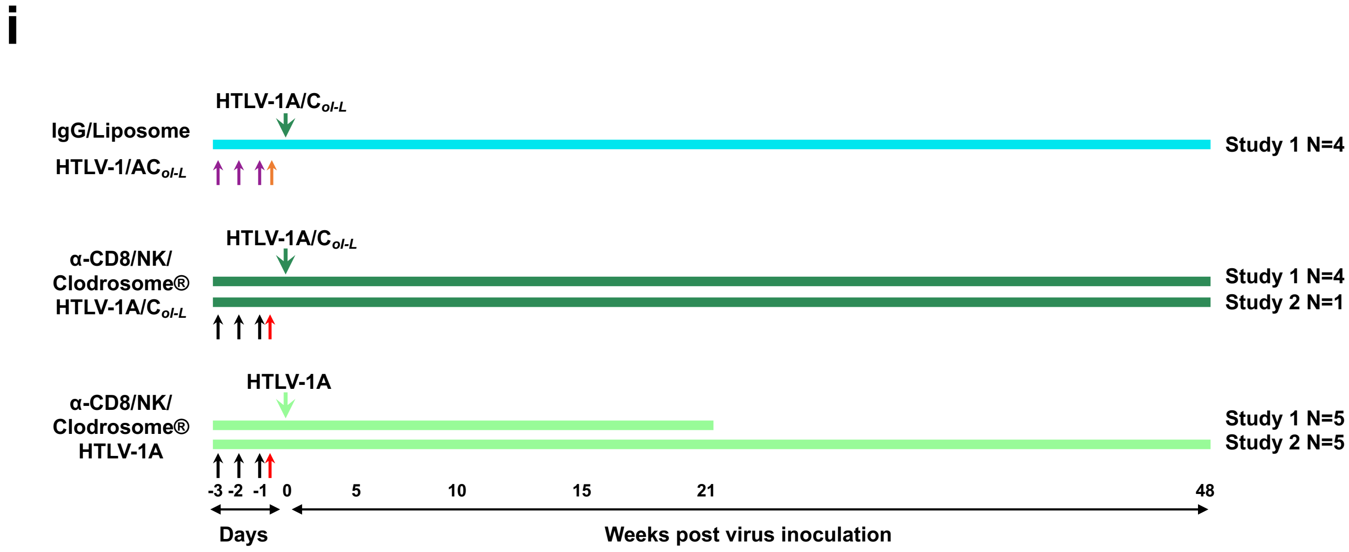

Supplementary Figure 4

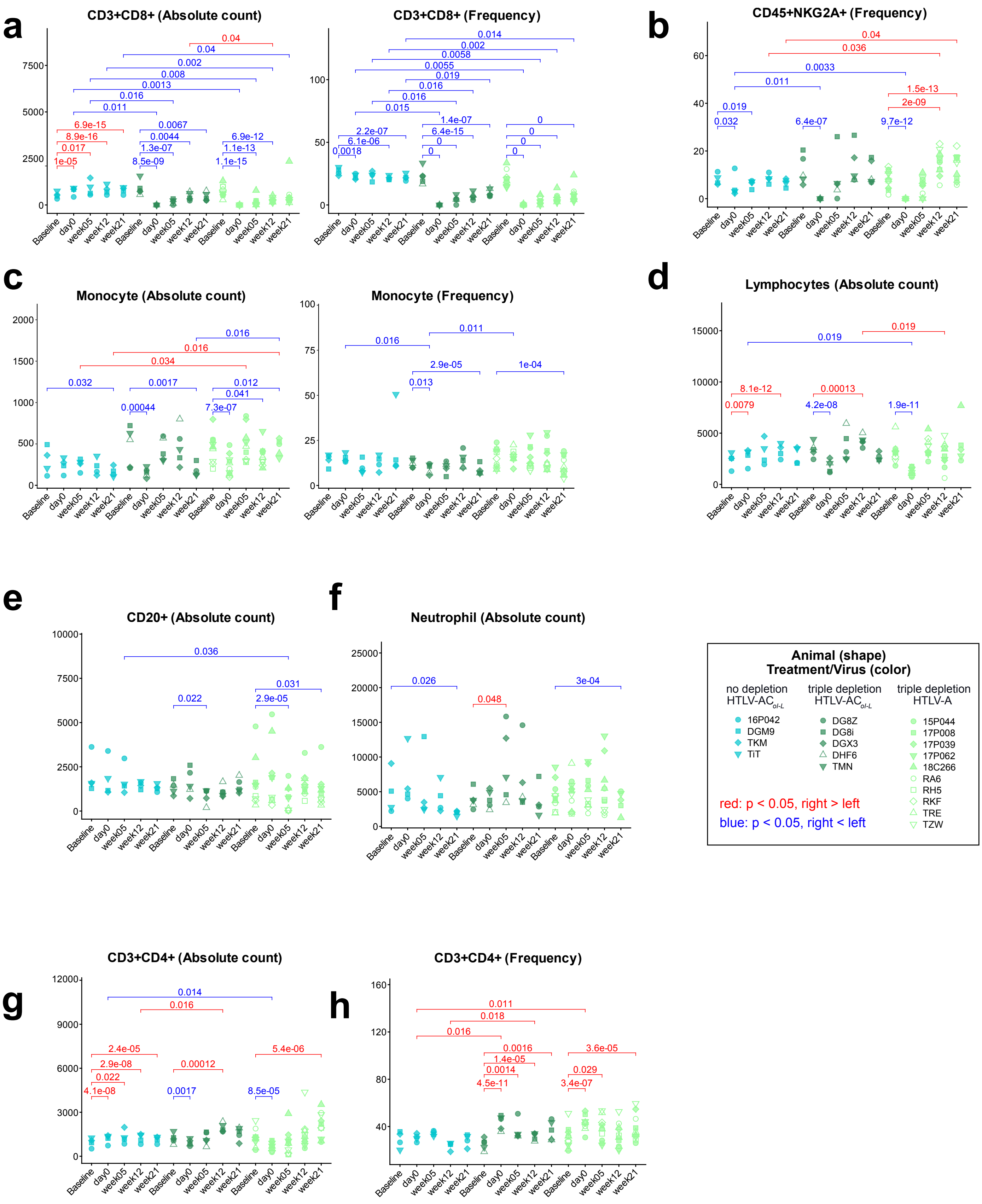

Supplementary Figure 5

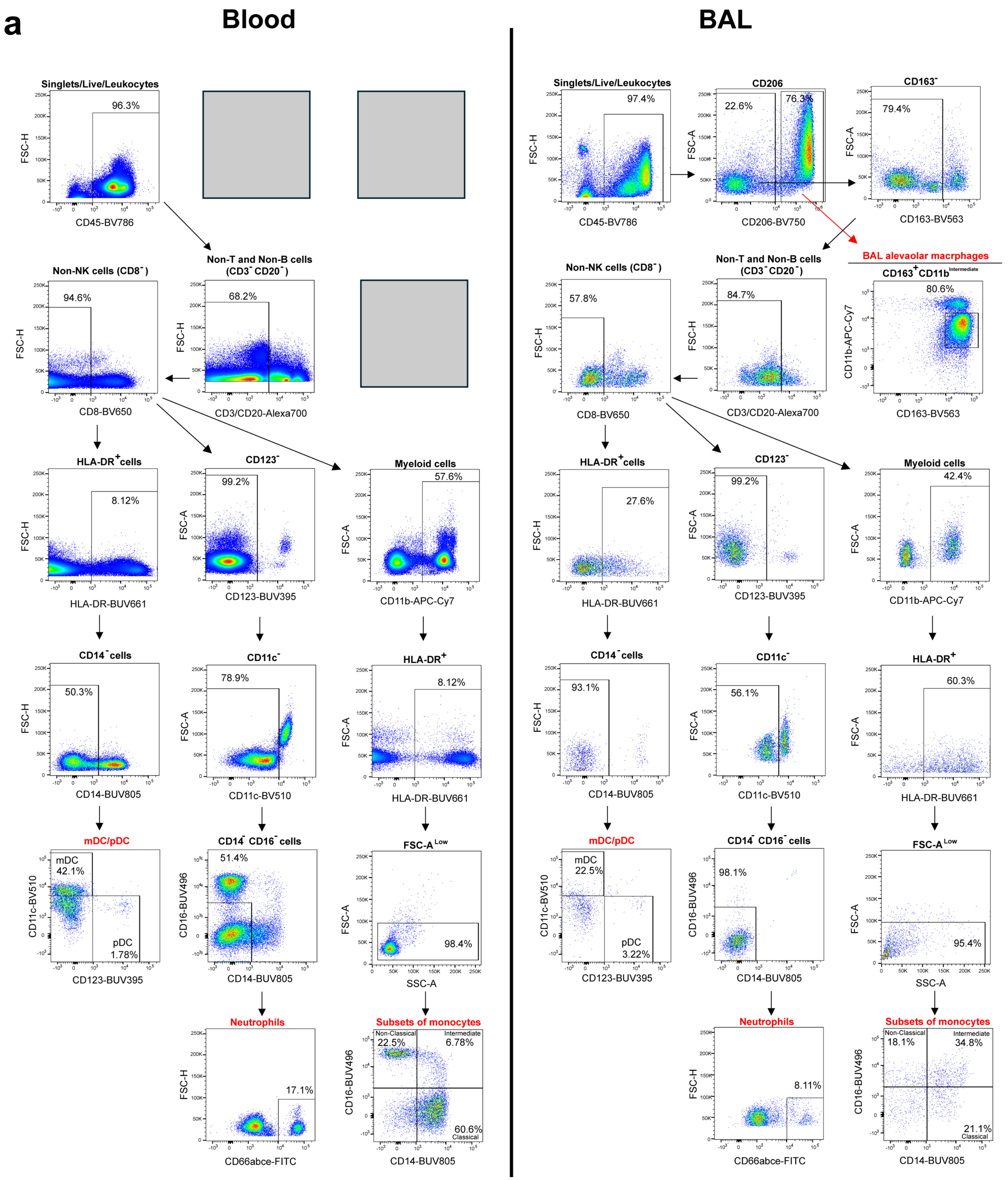

Supplementary Figure 5

b

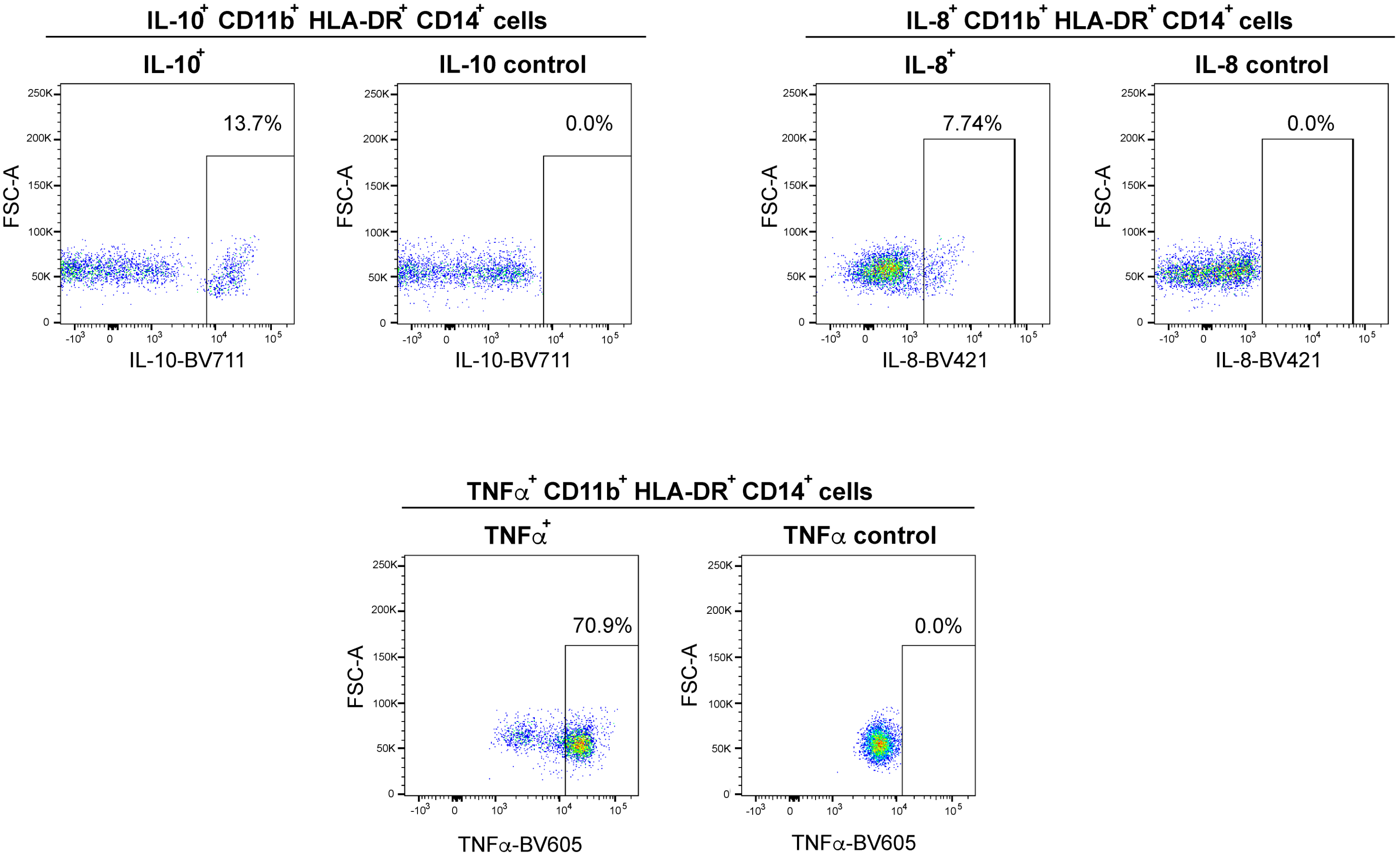

Supplementary Figure 6

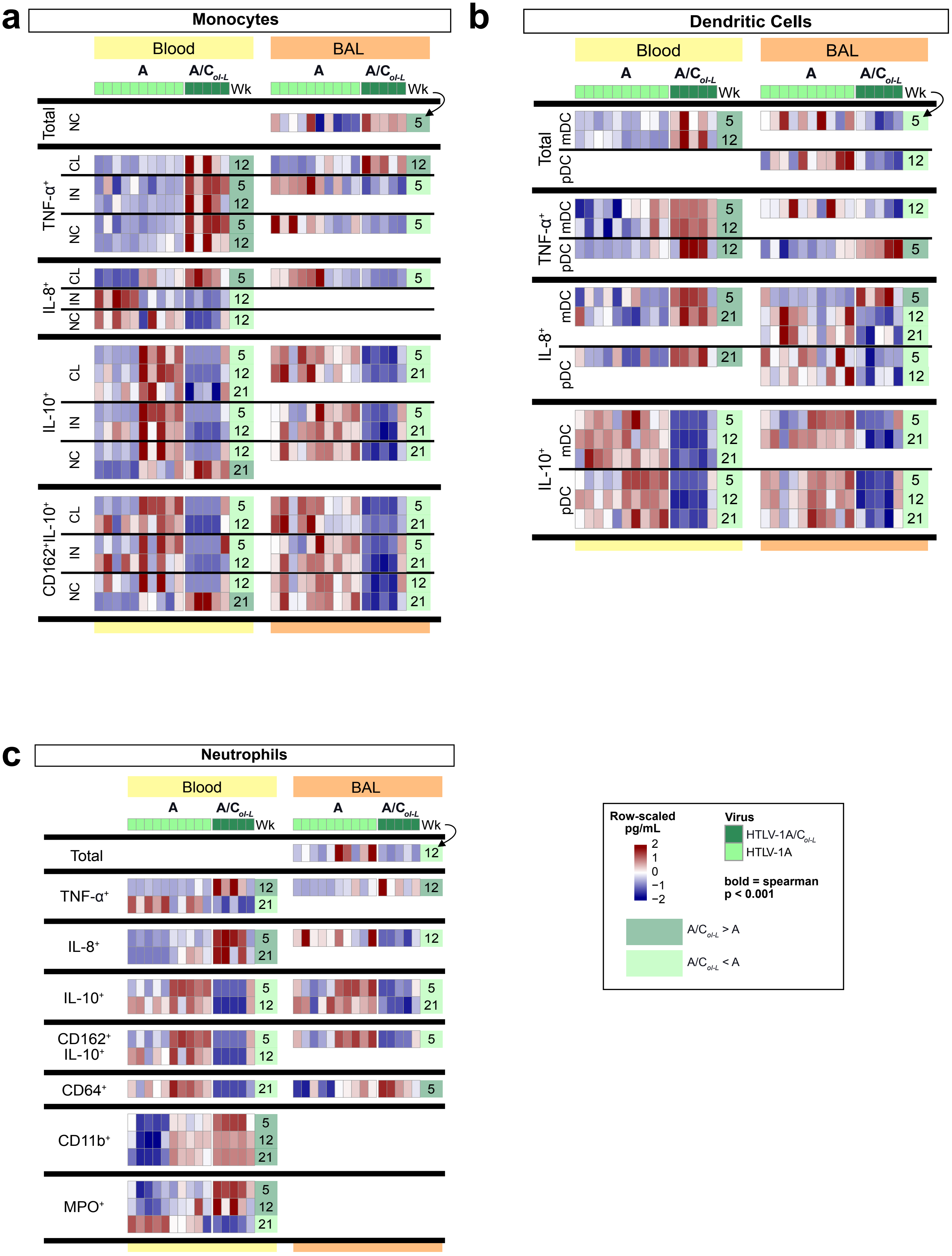

Supplementary Figure 7

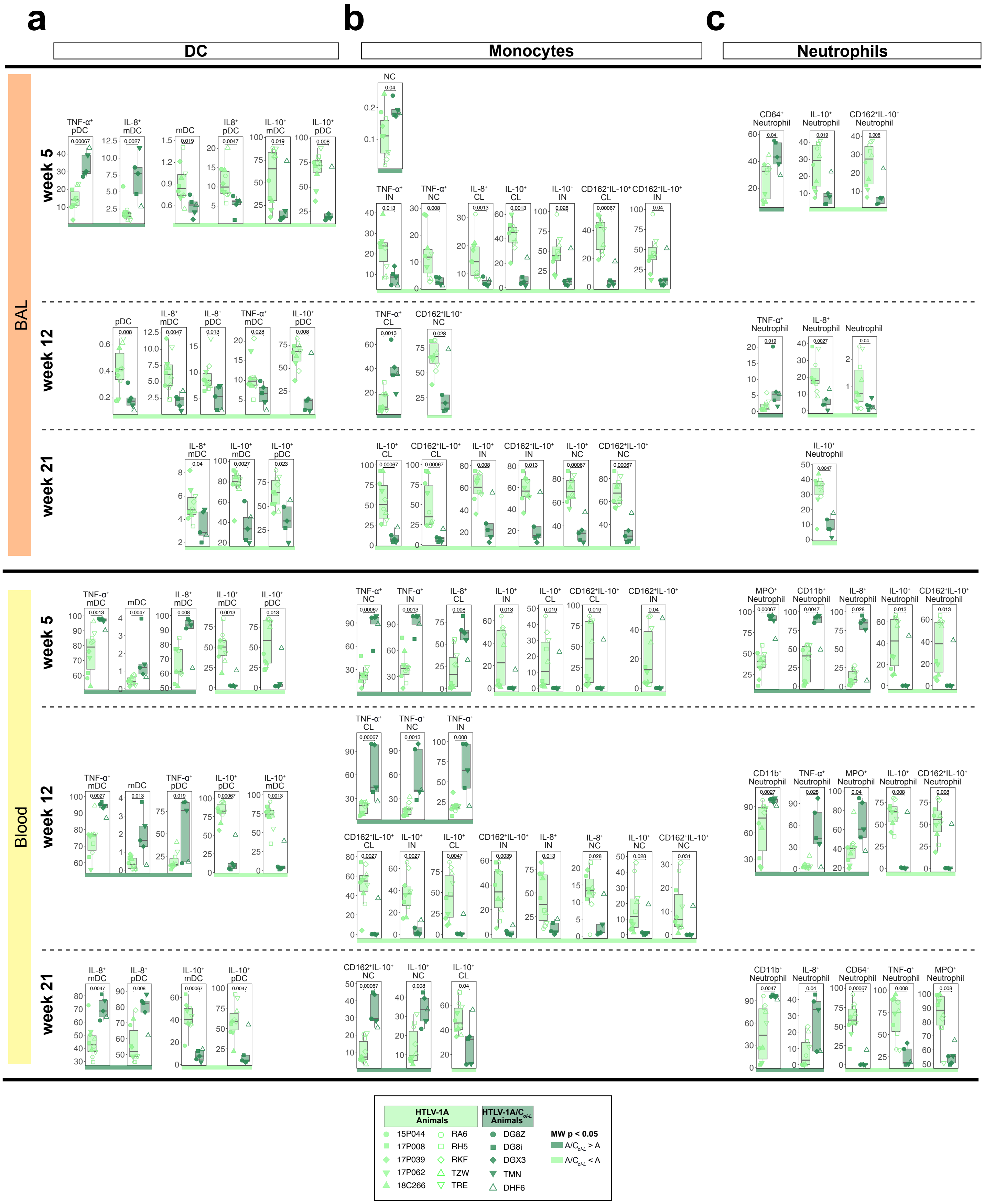

Supplementary Figure 8

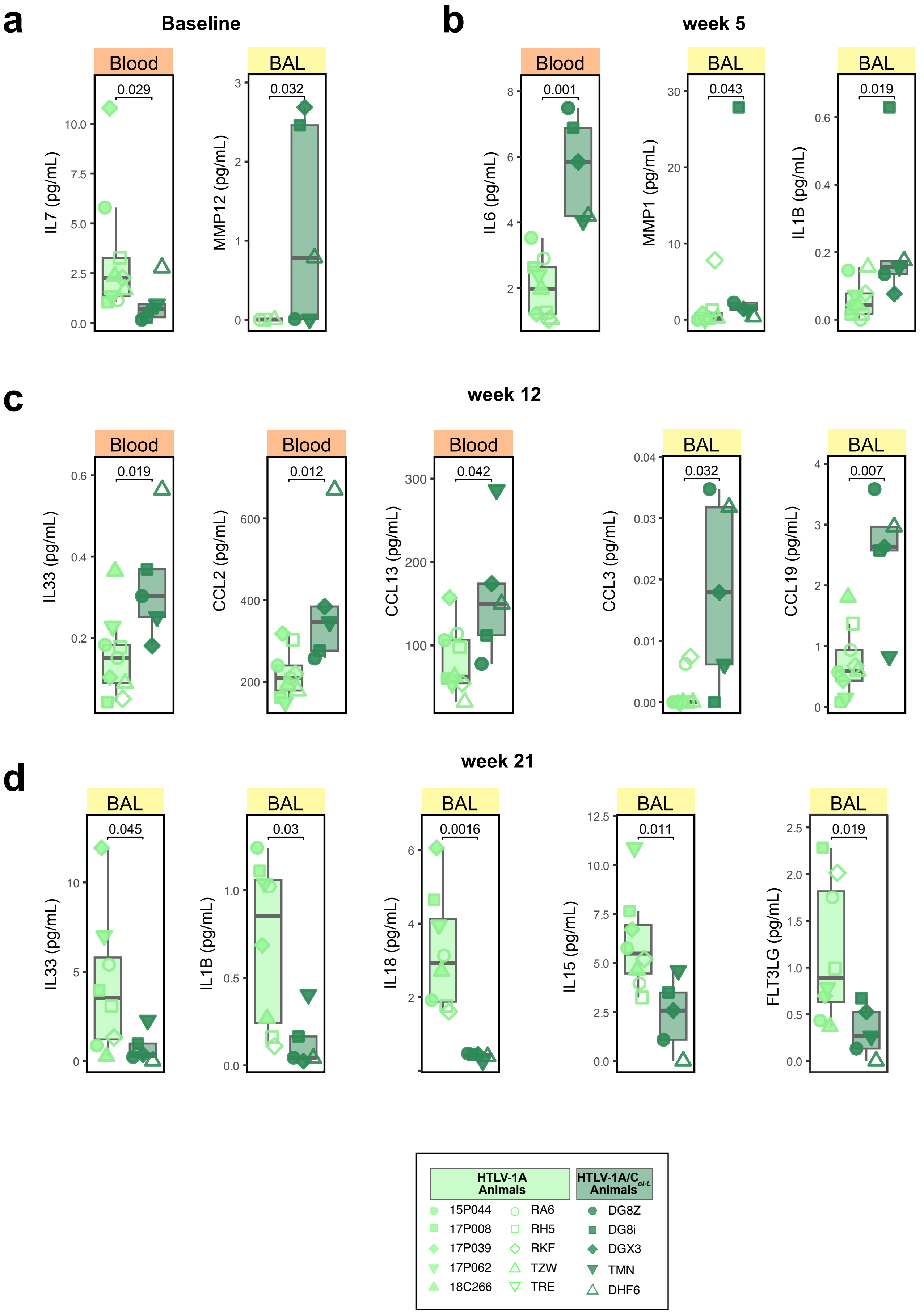

Supplementary Figure 9

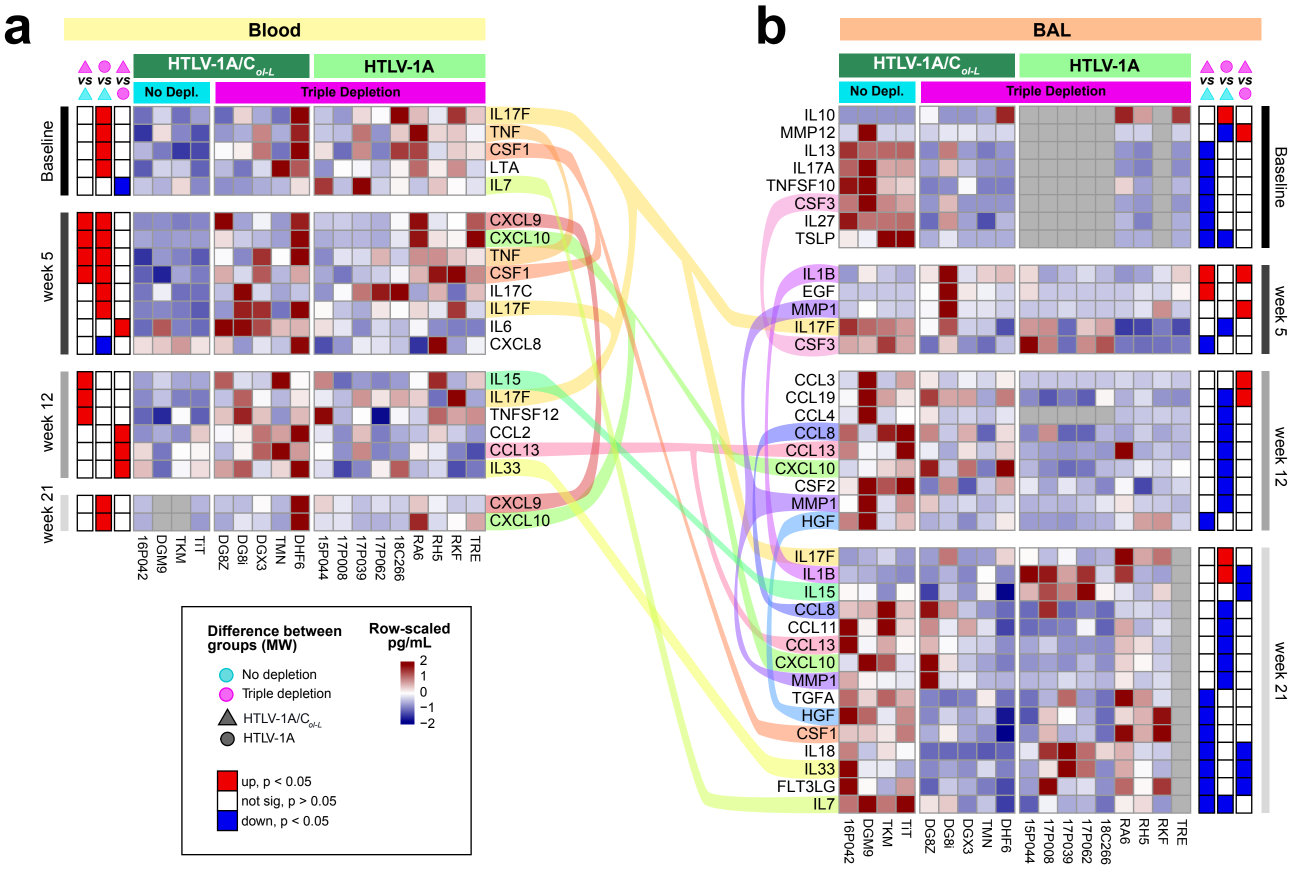

Supplementary Figure 10

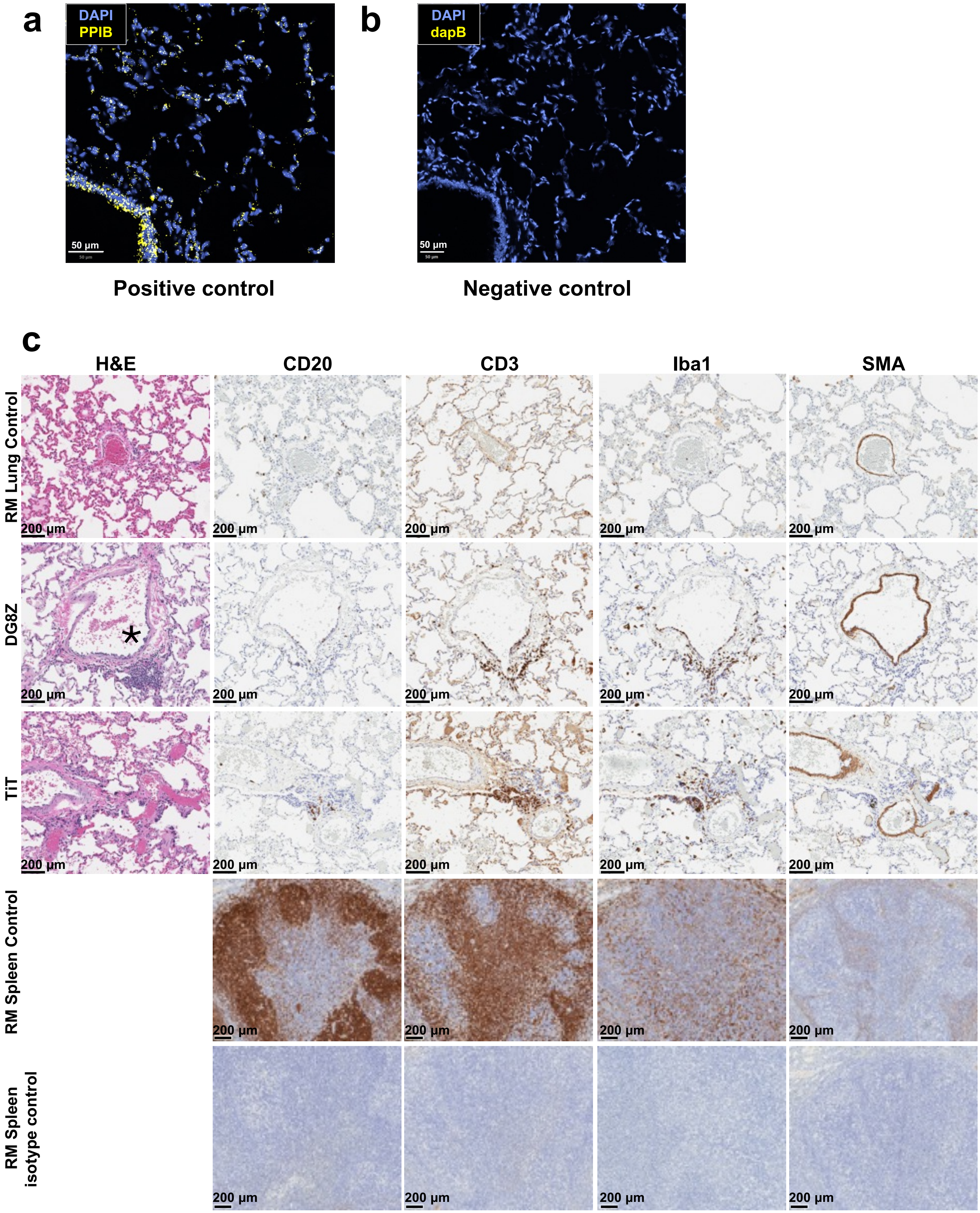

Supplementary Figure 11

a

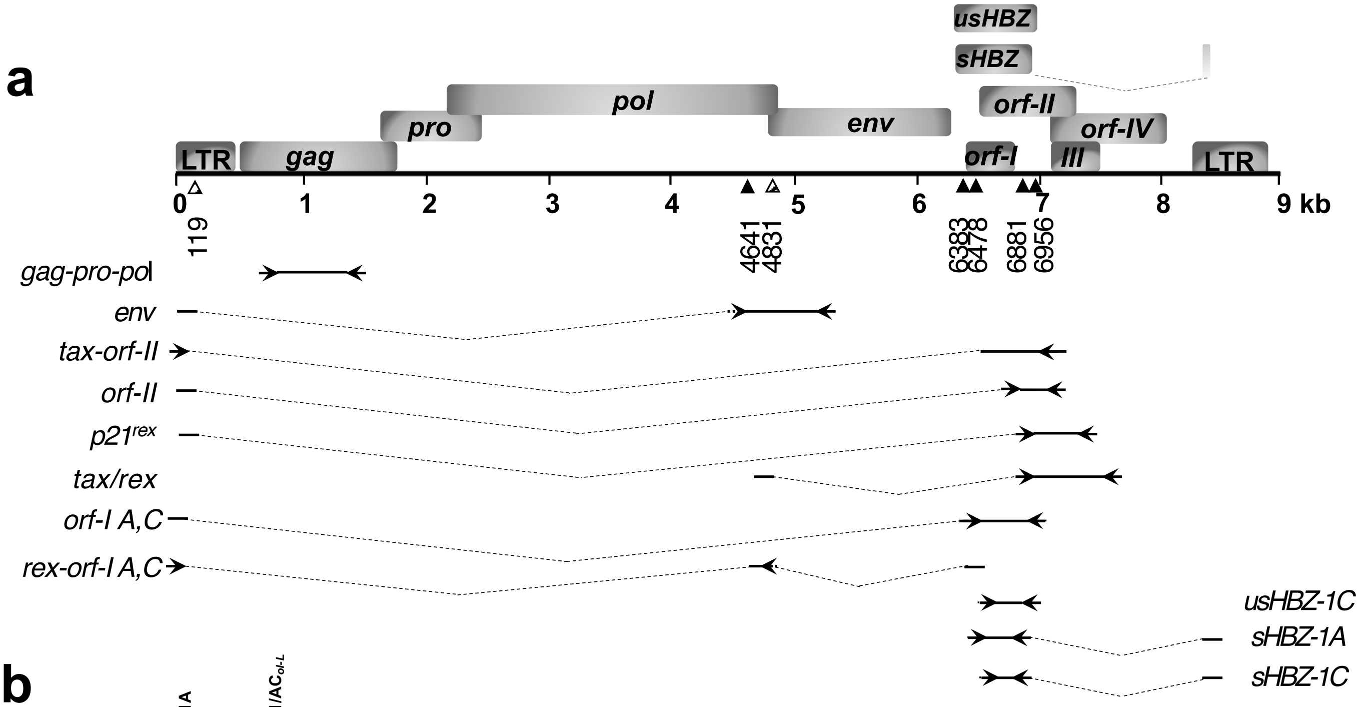

b

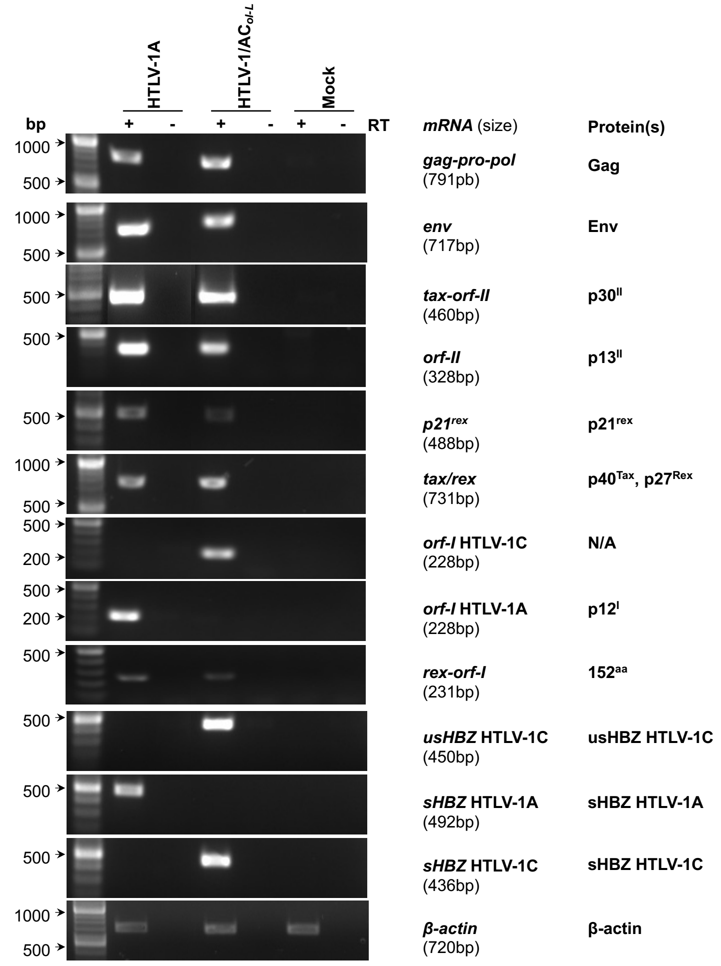

Supplementary Figure 11

c

```
Rex-orfI_A      MPKTRRRPRRSQRKRPPTPWQLPPFSLQGLHLAFQLSSIAINPQLLHFFFPSTMLFRL--   58
Rex-orfI_AC     MPKTRRRPRRSQRKRPPTPWQPPPFSLQGLCLALQLSSITINPQLRHSFSSSTTLPCLPS   60
                ********************* ******** **:*****:***** * *  ** *  *

Rex-orfI_A      -LSPLSPLALTALLLFLLSPGDVSGLLLRPPPAPCLLLFLPFQILSNLLFLLFLPLFFSL   117
Rex-orfI_AC     SLFCLSPLALAALLFLQFSPGAVGGLLLRPPPAPSLLLSLPFQKLSNLFLLLFFPLSFSL   120
                 *  ******:***:: :*** *.**********.*** **** ****::***:** ***

Rex-orfI_A      PLLLSPSLPITMRFPARWRFFPWRAPSQPAAAFLF*   152
Rex-orfI_AC     SLLLGPSPPITMRFPARWRFLFRRAPSQPAFFF*--   153
                 ***.** ************:  *******  *
```

- RNA Binding domain /Nuclear localization signal of Rex
- ER Retention/retrieval motif of p12
- Methionine for p12/p8
- IL-2Rβ and γ binding domain
- Calcineurin binding domain
- Ubiquitylation site of p12
- Transmembrane helices
- Cleavage site

d

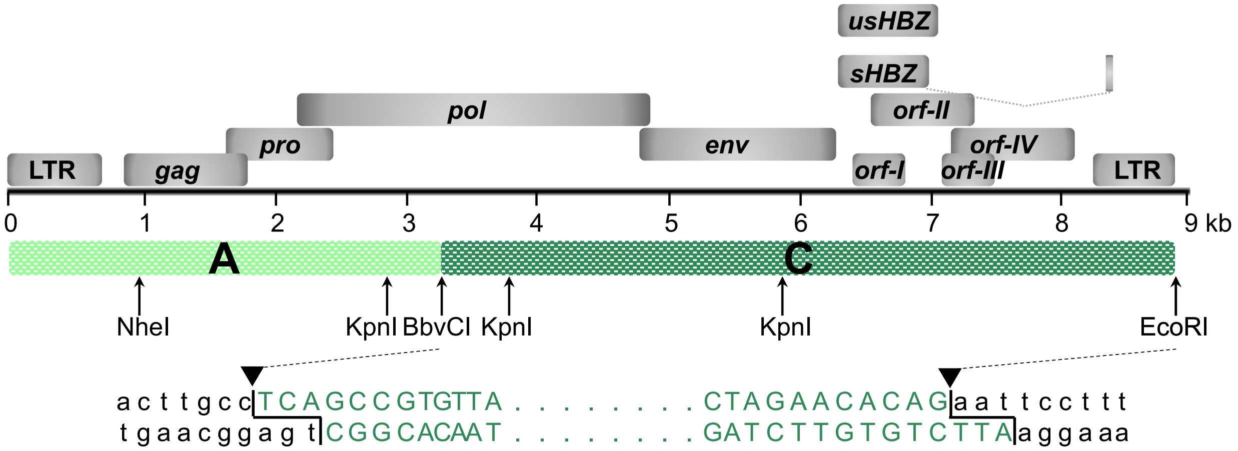

e

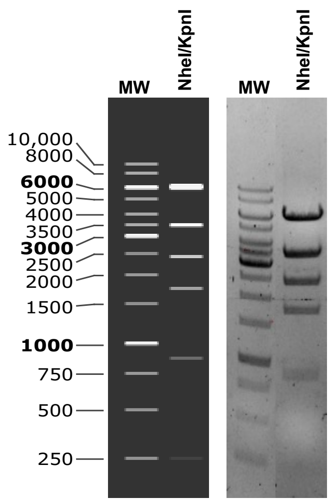

f

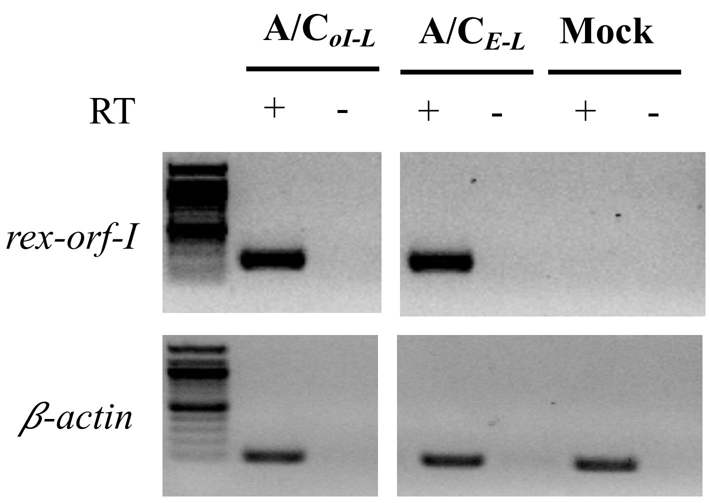

Supplementary Figure 12

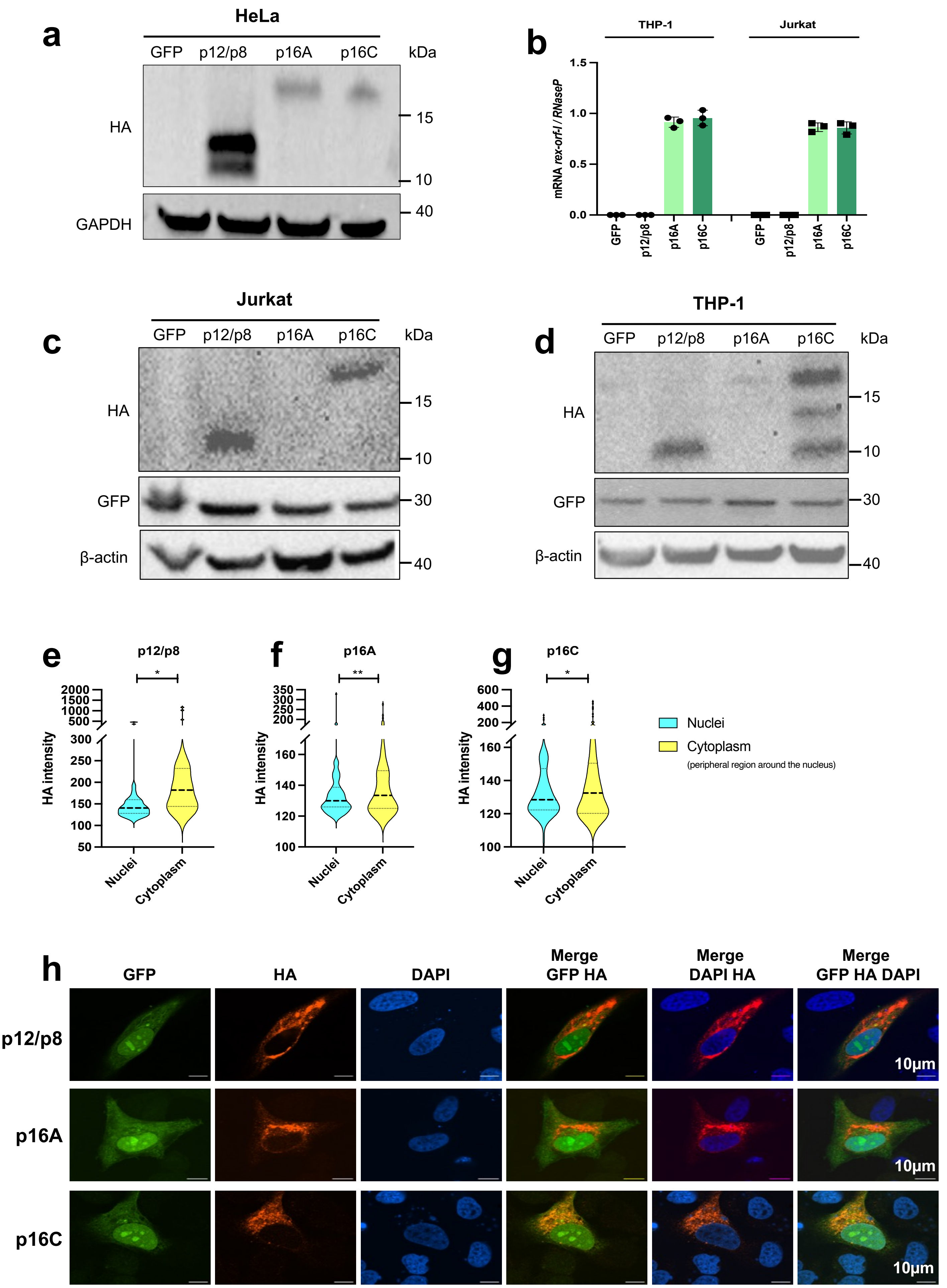

Supplementary Figure 13

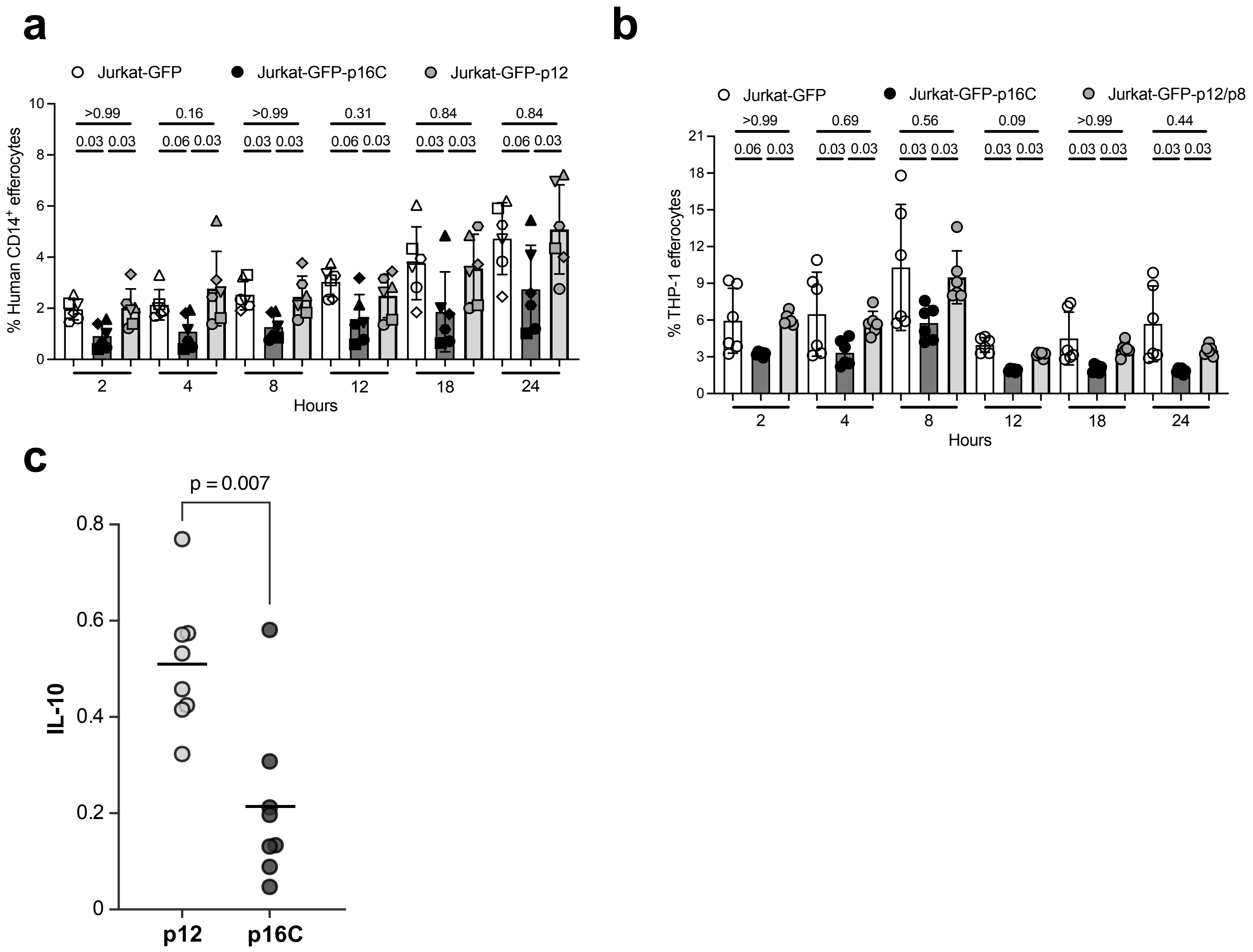

Supplementary Table 1

Group	Animal ID	Source	Treatment history	Viral exposure	Route of exposure	Sex	Virus PCR	Date of Birth
IgG/Liposome HTLV-1A/Co$_{l-L}$	TKM	Morgan Island	gp120$_{\Delta V1}$[1] vaccination	None	-	Female	SIV -	1/1/17
	TiT	Morgan Island	Naïve control	SIVmac251	Intravaginal	Female	SIV -	1/1/17
	DGM9	Morgan Island	Naïve control	SIVmac251	Intrarectal	Male	SIV -	1/1/17
	16P042	Covance, Alice Texas	4 X DNA /gp120$_{\Delta V1}$/ ALFQA[2]	SIVmac251	intrarectal	Male	SIV -	5/22/16
α-CD8/NK/Clodrosome ® HTLV-1A/C$_{ol-L}$	TMN	Morgan Island	SAMT-247 microbicide gel[3]	SIVmac251	Intravaginal	Female	SIV -	4/1/17
	DG8Z	Morgan Island	gp120$_{\Delta V1}$ vaccination + V2 Nanoparticles	SIVmac251	Intrarectal	Male	SIV -	3/10/17
	DGX3	Morgan Island	gp120$_{\Delta V1}$ vaccination + V2 Nanoparticles	SIVmac251	Intrarectal	Male	SIV -	1/1/17
	DG8i	Morgan Island	gp120$_{\Delta V1}$ vaccination + empty Nanoparticles	SIVmac251	Intrarectal	Male	SIV -	5/16/17
	DHF6	Morgan Island	ΔV1DNA+TTB-nanoparticles/ALVAC/ΔV1gp120_Alum+TTB-Nanoparticles	SIVmac251	Intrarectal	Male	SIV -	1/1/17
α-CD8/NK/Clodrosome ® HTLV-1A	15P044	Covance, Alice Texas	2 X DNA (A244HIVgp160$_{WT}$+HIVgagp55) / ALVAC-HIV / gp120$_{WT}$	SHIV-1157(QNE)Y173H	Intrarectal	Male	SIV -	5/3/15
	18C266	Covance, Alice Texas	2 X DNA (A244HIVgp160$_{WT}$+HIVgagp55) / ALVAC-HIV / gp120$_{WT}$	SHIV-1157(QNE)Y173H	Intrarectal	Male	SIV -	8/9/18
	17P008	Covance, Alice Texas	2 X DNA (A244HIVgp160$_{\Delta V1}$+HIVgagp55) / ALVAC-HIV / gp120$_{\Delta V1}$	SHIV-1157(QNE)Y173H	Intrarectal	Male	SIV -	3/25/17
	17P062	Covance, Alice Texas	Naïve control	SHIV-1157(QNE)Y173H	Intrarectal	Male	SIV -	9/17/17
	17P039	Covance, Alice Texas	Naïve control	SHIV-1157(QNE)Y173H	Intrarectal	Male	SIV -	6/9/17
	RA6	Morgan Island	gp120$_{\Delta V1}$ vaccination + SAMT-247	SIVmac251	Intravaginal	Female	SIV -	4/1/17
	TRE	Morgan Island	gp120$_{\Delta V1}$ vaccination + SAMT-247	SIVmac251	Intravaginal	Female	SIV -	1/1/17
	TZW	Morgan Island	gp120$_{\Delta V1}$ vaccination	SIVmac251	Intravaginal	Female	SIV -	4/29/17
	RH5	Morgan Island	naïve control + SAMT-247	SIVmac251	Intravaginal	Female	SIV -	1/1/17
	RKF	Morgan Island	naïve control + Empty Intra Vaginal Ring	SIVmac251	Intravaginal	Female	SIV -	5/1/19

Supplementary Table 2

a

HTLV-1A/C_{ol-L}		
**Animal ID**	**Lung Lobes**	**Histopathology diagnosis**
**DG8Z**	**Right Cranial**	Histiocytic pneumonia (lipid type) Type 2 cell hyperplasia
		Type 2 cell hyperplasia
	**Right Middle**	Bronchiectasis
		Dilated bornchioles
		Mild pneumonia
		Congestion and hemorrhage
	**Right Caudal**	Bronchiectasis
		Dilated bornchioles
		Mild pneumonia
		Congestion
		Edema
		Bacteria/contaminant
	**Accessory**	Bronchiectasis
		Dilated bornchioles
		Mild pneumonia
	**Left Cranial/Cranial**	Bronchiectasis
	**Left Cranial/Caudal**	Bronchiectasis with and
		Pneumonia
		Type 2 cell hyperplasia
	**Left Caudal**	Bronchiectasis with and
		Pneumonia
		Type 2 cell hyperplasia
**TMN**	**Right Cranial**	Multinucleated giant cells
		Histiocytes
		Type 2 cell hyperplasia
		Subpleural and interestitital fibrosis
	**Right Middle**	Mild subpleral fibrosis
		Alveolar histioytosis
		Type 2 cell hyperplasia
	**Right Caudal**	Mild subpleral fibrosis
		Alveolar histioytosis
	**Accessory**	Mild subpleral fibrosis
		Alveolar histioytosis
	**Left Cranial/Cranial**	Mild subpleral and interestitital fibrosis
		Foreign body granuloma
		Multinucleated giant cells are engulfing some foreign bodies
		Squamous metaplasia in bronchial epithlium
	**Left Cranial/Caudal**	Mild subpleral and interestitital fibrosis
		Foreign body granuloma
		Multinucleated giant cells are engulfing some foreign bodies
	**Left Caudal**	Mild subpleral and interestitital fibrosis
		Foreign body granuloma
		Multinucleated giant cells are engulfing some foreign bodies
		Histiocytes
**TiT**	**Right Cranial**	Bronchiectasis
		Hemorrhage
		Edmea
	**Right Middle**	Mild hemorrhage
		Congestion
		Edema
	**Right Caudal**	Hemorrhage
		Congestion
	**Accessory**	No Specific Findings
	**Left Cranial/Cranial**	Minimum focal pneumonia
	**Left Cranial/Caudal**	Edema
		Congestion
	**Left Caudal**	Hemorrhage
		Congestion

b

HTLV-1A		
**Animal ID**	**Lung Lobes**	**Histopathology diagnosis**
**TRE**	**Right Cranial**	Mild pleural fibrosis
	**Right Middle**	Mild pleural and interstitial (focal) fibrosis
	**Right Caudal**	Mild pleural fibrosis
	**Accessory**	No specific findings
	**Left Cranial/Cranial**	Moderate pleural and interstitial (Focal) fibrosis
	**Left Cranial/Caudal**	Mild pleural fibrosis
	**Left Caudal**	Mild pleural and interstitial (focal) fibrosis
**RH5**	**Right Cranial**	Mild interstitial fibrosis
	**Right Middle**	Mild interstitial fibrosis
	**Right Caudal**	No specific findings
	**Accessory**	No specific findings
	**Left Cranial/Cranial**	No specific findings
	**Left Cranial/Caudal**	No specific findings
	**Left Caudal**	No specific findings
**RKF**	**Right Cranial**	Moderate pleural and interstitial (Focal) fibrosis
	**Right Middle**	No specific findings
	**Right Caudal**	No specific findings
	**Accessory**	No specific findings
	**Left Cranial/Cranial**	Mild pleural and interstitial (focal) fibrosis
	**Left Cranial/Caudal**	Mild pleural fibrosis
	**Left Caudal**	No specific findings

Supplementary Table 3

a

Lung Lobes	Animal ID	DNA: Gag	DNA: orfI-II	mRNA: *gag*	mRNA: *rex-orf-I*	mRNA: *tax*	mRNA: *usHBZ*	mRNA: *sHBZ*	Histopathology: Bronchiectasis	Histopathology: Fibrosis
		HTLV-1A								
Right Cranial	RA6	□	□	□	□	□	□	□	nd	nd
	TRE	□	□	□	□	□	□	□	-	Mild
	TZW	□	□	□	□	□	□	□	nd	nd
	RH5	□	□	□	□	□	□	□	-	Mild
	RKF	□	□	□	□	□	□	□	-	Moderate
Right Middle	RA6	□	□	□	□	□	□	□	nd	nd
	TRE	□	□	□	□	□	■	□	-	Mild
	TZW	□	□	□	□	□	□	□	nd	nd
	RH5	□	□	□	■	□	□	□	-	Mild
	RKF	□	□	□	□	□	□	□	-	-
Right Caudal	RA6	□	□	□	□	□	□	□	nd	nd
	TRE	□	□	□	□	□	□	□	-	Mild
	TZW	□	□	□	□	□	□	□	nd	nd
	RH5	□	□	□	□	□	□	□	-	-
	RKF	■	□	□	□	□	□	□	-	-
Accessory	RA6	□	□	□	□	□	□	□	nd	nd
	TRE	□	□	□	□	□	□	□	-	-
	TZW	□	□	□	□	□	□	□	nd	nd
	RH5	■	□	□	□	□	□	□	-	-
	RKF	□	□	□	□	□	□	□	-	-
Left Cranial / Cranial	RA6	□	□	□	□	□	□	□	nd	nd
	TRE	□	□	□	□	□	■	□	-	Mild
	TZW	■	□	□	■	□	□	□	nd	nd
	RH5	□	□	□	□	□	□	□	-	-
	RKF	□	□	□	□	□	□	□	-	Mild
Left Cranial / Caudal	RA6	□	□	□	□	□	□	□	nd	nd
	TRE	□	□	□	■	□	□	□	-	Mild
	TZW	□	□	□	■	□	□	□	nd	nd
	RH5	□	□	□	■	□	□	□	-	-
	RKF	□	□	□	□	□	□	□	-	Mild
Left Caudal	RA6	□	□	□	□	□	□	□	nd	nd
	TRE	□	□	□	■	□	□	□	-	Mild
	TZW	□	□	□	■	□	□	□	nd	nd
	RH5	□	□	□	□	□	□	□	-	-
	RKF	□	□	□	□	□	□	□	-	-

■	Detected
□	Not Detected
+	Detected
-	Not Detected
nd	Not done

b

Lung Lobes	Animal ID	DNA: Gag	DNA: orfI-II	mRNA: *gag*	mRNA: *rex-orf-I*	mRNA: *tax*	mRNA: *usHBZ*	mRNA: *sHBZ*	Histopathology: Bronchiectasis	Histopathology: Fibrosis
		HTLV-1A/C_{ol-L}								
Right Cranial	DG8Z	□	□	■	■	□	■	□	-	-
	TMN	□	□	■	□	■	□	■	-	Mild
	TiT	□	□	■	■	□	□	■	+	-
	DHF6	■	■	□	■	□	■	■	nd	nd
Right Middle	DG8Z	□	■	■	■	□	□	■	+	-
	TMN	□	□	□	□	□	□	□	-	Mild
	TiT	□	□	□	■	■	□	□	-	-
	DHF6	■	■	□	□	□	■	□	nd	nd
Right Caudal	DG8Z	□	□	■	□	□	□	■	+	-
	TMN	□	□	□	■	■	□	■	-	Mild
	TiT	□	■	■	■	□	□	■	-	-
	DHF6	■	□	□	■	□	□	■	nd	nd
Accessory	DG8Z	□	□	■	□	□	□	■	+	-
	TMN	□	□	■	□	□	□	□	-	Mild
	TiT	□	□	■	■	■	□	■	-	-
	DHF6	□	□	□	■	□	■	■	nd	nd
Left Cranial / Cranial	DG8Z	□	■	□	□	□	□	□	+	-
	TMN	□	□	■	□	□	□	□	-	Mild
	TiT	□	□	■	■	□	□	□	-	-
	DHF6	■	□	□	■	□	□	■	nd	nd
Left Cranial / Caudal	DG8Z	□	□	■	□	□	□	□	+	-
	TMN	□	□	■	□	■	□	■	-	Mild
	TiT	□	□	■	□	□	□	□	-	-
	DHF6	■	■	□	□	□	■	■	nd	nd
Left Caudal	DG8Z	□	□	■	■	□	□	□	+	-
	TMN	□	□	■	■	■	□	■	-	Mild
	TiT	■	□	■	■	□	□	□	-	-
	DHF6	□	■	□	■	□	■	■	nd	nd

Supplementary Table 4

Animal ID	Tested lung lobes	HTLV-1 *gag* RNAscope
**TMN**	Right Cranial	Rare positive cells, perivascular immune cells
	Left Cranial / Cranial	Not detected
**DG8Z**	Left Cranial / Cranial	Not detected
	Right Caudal	Not detected
	Right middle	Not detected
**TiT**	Left caudal	Rare positive cells, submucosal, large airways
	Right caudal	Not detected
**Control**	AUP038M	Not detected

Supplementary Table 5

Transcript	Primer	Sequences 5'-3'	Position (nucleotide)*	Amplicon size (bp)	Technical approach	Application
gag-pro-pol	Gag_HTLV-1C_Fr	AGCCAGCCTACTCCCAAAAG	653	791	RT-PCR (1st)	Virus characterization
	Gag_HTLV-1C_Rv	AAGCCCGCAACATATCTCC	1443		RT-PCR (1st and 2nd)	
	Gag_HTLV-1_Fr_Nest	CCAAGTCCTTCCAGTCATGC	830	614	RT-PCR (1st)	Virus detection
	qPCR_HTLV-1_gag_Fr	CCCATCTTACGTTCCTTAGCC	1338	132	qRT-PCR	Virus detection
	qPCR_HTLV-1_gag_Rv	TTTGTCTTTGGGGGTCCAG	1469		qRT-PCR	
env	Env_HTLV-1C_SJ_Fr	CGTCTAGCTTCCTGGTCTTAATAG	CGTCTAG$^{118\wedge4642}$CTTCCTGGTCTTAATAG	717	RT-PCR	Virus characterization
	Env_HTLV-1C_Rv	ACCAGATGGGGTCATATCCT	5351		RT-PCR	
tax-orf-II	LTR_HTLV-1A_Fr	CTGTGGTGCCTCCTGAACTAC	85	460	RT-PCR	Virus characterization
	p30_HTLV1C_Rv	GGTCCCAGGTGATCTGATGCT	7124		RT-PCR	
orf-II	p13_HTLV-1C_SJ_Fr	GCCGTCTAGCAGGTCCTCC	GCCGTCTAG$^{118\wedge6892}$CAGGTCCTCC	328	RT-PCR	Virus characterization
	p13_HTLV-1C_Rv	GTTCTCTGGGTGGGGAAGG	7200		RT-PCR	
p21 ^{rex}	p21_HTLV-1A/C_SJ_Fr	CGCCGTCTAGCCCACTTC	CGCCGTCTAG$^{118\wedge6957}$CCCACTTC	488	RT-PCR	Virus characterization
	p21_HTLV1A/C_Rv	GGGGAAAGCTGGTAGAGGTA	7434		RT-PCR	
tax/rex	Tax/Rex_HTLV-1C_SJ_Fr	ACCAACACCATGGCCCAC	ACCAACACCATGG$^{4831\wedge6950}$CCCAC	731	RT-PCR	Virus characterization
	Tax/Rex_HTLV-1C_Rv	AGGGTTGATTGGAACGGAAG	7674		RT-PCR	
	qPCR_Tax_Fr	TCCTCACCAATGTTCCCTACA	7497	189	qRT-PCR	Virus detection
	qPCR_Tax_Rv	CTGGAGTGGTGAGGGTTGA	7685		qRT-PCR	
orf-I HTLV-1A	p12_HTLV-1A_SJ_Fr	CGTCTAGCACTATGCTGTTTCG	CGTCTAG$^{118\wedge6479}$CACTATGCTGTTTCG	228	RT-PCR	Virus characterization
	p12_HTLV-1A_Rv	GGCTGAGGAGAAGAGGAAGC	6680		RT-PCR	
orf-I HTLV-1C	p12_HTLV-1C_SJ_Fr	CGTCTAGCACTACGCTGCCTTG	CGTCTAG$^{118\wedge6479}$CACTACGCTGCCTTG	228	RT-PCR	Virus characterization
	p12_HTLV-1C_Rv	GGCCGAGGAGAAGAGAAAGC	6680		RT-PCR	
rex-orf-IA	LTR_HTLV-1_Fr	CTGTGGTGCCTCCTGAACTAC	85	231	RT-PCR	Virus characterization
	Rex-orf-I_HTLV-1C_SJ_Rv	AGGTTGCCATGGTGTTGGT	AGGTTG$^{6383\wedge4832}$CCATGGTGTTGGT		RT-PCR	
	Rex-orf-I_HTLV-1A_Fr_Nest	TATAGCCATCAATCCCCAAC	6436	262	RT-PCR (1st and 2nd)	Virus detection
	Rex-ORFI_HTLV-1A_Rv	GCTGAGGAGAAGAGGAAGC	6698		RT-PCR (1st)	
	Rex-orf-IA_Rv_6603	AAGGAAGAGGAGAAGGCACGG	6623	187	RT-PCR (2nd)	
rex-orf-IC	LTR_HTLV-1_Fr	CTGTGGTGCCTCCTGAACTAC	85	231	RT-PCR	Virus characterization
	Rex-orf-I_HTLV-1C_SJ_Rv	AGGTTGCCATGGTGTTGGT	AGGTTG$^{6383\wedge4832}$CCATGGTGTTGGT		RT-PCR	
	Rex-orf-I_HTLV-1C_Fr_Nest	CATAACCATCAACCCCCAAC	6437	262	RT-PCR (1st and 2nd)	Virus detection
	Rex-ORFI_HTLV-1C_Rv	GCCGAGGAGAAGAGAAAGC	6698		RT-PCR (1st)	
	Rex-orf-I_Rv_6603	AAGAGAGAGAAGGAGGCTTGG	6623	187	RT-PCR (2nd)	
	Rex-orf-I_AC-1PCR-Fr	CCGCCGATCCCAAAGAAAA	4796	135	RT-PCR (1st)	
	Rex-orf-I_AC-1PCR-Rv	GGGGTTGATGGTTATGCTAGAC	6452		RT-PCR (1st)	
	qPCR-Rex-orf-I-Fr	CGATCCCAAAGAAAAAGACC	4800	63	qRT-PCR (2nd)	
	qPCR-Rex-orf-I-Rv	GAGTCCTTGGAGGCTGAAC	6413		qRT-PCR (2nd)	
	MGBqPCR-Rex-orf-I-probe	CAACACCATGG$^{\wedge}$CAACCTCCTCC	4822		qRT-PCR (2nd)	
usHBZ HTLV-1A	HBZsUSf	GTTAACTTTGTATCTGTAGGG	6941	497	RT-PCR (1st)	Virus detection
	sHBZ_HTLV-1A_Rv	TAGCCATCAACCCCCAACTC	6439		RT-PCR (1st and 2nd)	
	usHBZ_HTLV-1C_Fr_Nest	GGAGGAGGAAGCTGTGCTTG	6819	378	RT-PCR (2nd)	
	sHBZ_HTLV-1A_Rv	TAGCCATCAACCCCCAACTC	6439		RT-PCR (1st and 2nd)	
usHBZ HTLV-1C	usHBZ_HTLV-1C_Fr	ATGATTAACTTTGTATTTGTAGGGCC	6944	450	RT-PCR (1st)	Virus characterization and Virus detection
	usHBZ_HTLV-1C_Rv	CTTCCCAGCTCCTTGTTCTG	6495		RT-PCR (1st and 2nd)	
	usHBZ_HTLV-1C_Fr_Nest	GGAGGAGGAATCTGTGCTTG	6819	325	RT-PCR (2nd)	Virus detection
sHBZ HTLV-1A	sHBZ_HTLV-1A_Fr	CCTCAGGGCTGTTTCGATG	CCTCAG$^{8324\wedge6922}$GGCTGTTTCGATG	484	RT-PCR	Virus characterization
	sHBZ_HTLV-1A_Rv	TAGCCATCAACCCCCAACTC	6439		RT-PCR	
	sHBZ_HTLV-1A_Fr	CCTCAGGGCTGTTTCGATG	CCTCAG$^{8324\wedge6922}$GGCTGTTTCGATG	492	RT-PCR (1st)	Virus detection
	sHBZ_HTLV-1A_Rv	TAGCCATCAACCCCCAACTC	6439		RT-PCR (1st and 2nd)	
	usHBZ_HTLV-1C_Fr_Nest	GGAGGAGGAAGCTGTGCTTG	6819	381	RT-PCR (2nd)	
	sHBZ_HTLV-1A_Rv	TAGCCATCAACCCCCAACTC	6439		RT-PCR (1st and 2nd)	
sHBZ HTLV-1C	sHBZ_HTLV-1C_SJ_Fr	GGCCTCAGGGCCATTTCG	GGCCTCAG$^{8324\wedge6922}$GGCCATTTCG	436	RT-PCR (1st)	Virus characterization and Virus detection
	sHBZ_HTLV-1C_Rv	CTTCCCAGCTCCTTGTTCTG	6495		RT-PCR (1st and 2nd)	
	sHBZ_HTLV-1C_Fr_Nest	GGAGGAGGAATCTGTGCTTG	6819	325	RT-PCR (2nd)	Virus detection

Supplementary Fig. 1. Restriction enzyme characterization of HTLV-1A/C_{oI-L} and comparison of Tax amino-acid sequences to HTLV-1A. (a) HTLV-1A/C_{oI-L} clones were screened using SpeI and NheI plus KpnI. The left panel indicates the expected restriction digest fragments separated by electrophoresis on a 0.7% agarose gel. The right panel is the digestion pattern of the HTLV-1A/C_{oI-L} clone. (b) Representative western blot analysis of three independent experiment of Tax expression in HEK293T cells transiently transfected with HTLV-1A, HTLV-1A/C_{oI-L} molecular clones, or mock transfected using different Tax antibodies (Tax-1A3 and Tax-LT-4). The HTLV-1 transformed cell lines C8166 were used as positive control for Tax expression. (c) Amino acid sequence alignments of HTLV-1A (HTLV-1A-Tax) and HTLV-1A/C_{oI-L} (HTLV-1C-Tax) Tax viral protein. Functional and structural domains required for Tax activity are highlighted as labeled for Nuclear Localization Signal (NLS) at the N-terminus, CREB-1 activation region, CBP/p300 binding domain, IKK binding domain, leucine zipper domain (LZD), NF-κB activation region, Nuclear Export Signal (NES), second leucine zipper domain, ATF/CREB activation domain, and a PDZ binding domain at the C-terminus. The multi-alignments were performed with the Clustal O (1.2.4) program using default parameters. Asterisks (*), colons (:), periods (.), and spaces ( ) indicate that the amino acids at a given position are respectively identical, conserved, semi-conserved, or different, between the two sequences. Source data are provided as a Source Data file.

Supplementary Fig. 2. Blood cell population frequency in depleted or replete animals before exposure to HTLV-1A/C_{oI-L}. Scatterplots illustrate frequency or absolute count of cell populations for baseline and Day 0. Day 0 correspond to the day following the three consecutive days (Day -3,-2,-1) of treatment with either MT807R1 or IgG control antibodies, and the treatments (Day -1) with either Clodrosome® or Liposome control and prior to HTLV-1A/C_{oI-L} inoculation. Each teal and dark green symbols represents an animal from the non-depleted (n=4) and triple depleted (n=5) groups inoculated with HTLV-1A/C_{oI-L} virus, respectively. The p-values between timepoints within a treatment group were calculated by fitting generalized estimating equations with animal as a random effect, while p-values between treatment groups for the same timepoint were calculated by Mann-Whitney/Wilcoxon non-parametric tests. The p-values are shown for all comparisons with $p<0.05$. (a-d) Comparisons of cell subsets at baseline and Day 0 for each virus: (a) $CD8^+$ (left and middle panels) and NK cells (right); (b) Monocytes; (c) $CD4^+$; and (d) $CD20^+$ cells (far left), lymphocytes (left), neutrophils (right) and eosinophils (far right). Source data are provided as a Source Data file.

Supplementary Fig. 3. Seroconversion and systemic virus detection in animals exposed to HTLV-1A/C_{oI-L} or HTLV-1A. (a-d) Study 1: Virological and immunological parameters of (a, b) triple depleted (n=4) and (c, d) replete animals (n=4) from study 1. (a, c) Sera from all animals were assayed for reactivity to HTLV-1 antigens and the week of sera collection is indicated below each western blot strip. Heatmaps depict the detection (black) or absence of detection (gray) of HTLV-1 *gag* and *orf-I/II* gene regions by Nested PCR in the blood, lymph nodes, bone marrow, and BAL samples throughout the course of the study. (b, d) HTLV-1 p24Gag antibody titers in blood were measured in all animals at baseline (B) and after virus exposure (weeks 3, 5, 7, 10, 12, 16, and 21). Undiluted, or dilutions of 1:10, 1:100, 1:1000, and 1:10000 sera were used to assess

titers, coded in grey by intensity. (e-h) Study 2: Virological and immunological parameters in triple depleted animals (n=5) exposed to (e, g) HTLV-1A or (f, h) HTLV-1A/C_{oI-L} (n=1) from study 2. (i) Study 1 and 2 design schematic. Black and purple arrows represent the 3 consecutive days of treatment with either MT807R1 or IgG control antibodies, respectively, while red and orange arrows represent the treatments with either Clodrosome® or Liposome® control. Dark green and light green arrows indicate the inoculation day (Day 0) of the lethally γ-irradiated 729.6 B-cell line producing HTLV-1A/C_{oI-L} or HTLV-1A viruses respectively. In Study 2, some subsequent analysis include data from historical animals exposed in identical conditions to HTLV-1A (n=5) as described in a prior study[39]. Source data are provided as a Source Data file and Supplementary Data file.

Supplementary Fig. 4. Changes in blood cell populations following infection with HTLV-1A or HTLV-1A/C_{oI-L}. (a-g) Summary of cell population changes with time for the triple depleted groups inoculated with HTVL-1A or HTLV-1A/C_{oI-L}. Changes are shown for (a) $CD8^+$ T cells, (b) NK cells, (c) monocytes, (d) lymphocytes, (e) $CD20^+$ B cells, (f) neutrophils, and (g-h) $CD4^+$ T cells. Scatterplots illustrate frequency or absolute count of cell populations for baseline and subsequent timepoints following the viral inoculation. Teal corresponds to animals (n=4) belonging to the non-depleted group inoculated with HTLV-1A/C_{oI-L}, dark green corresponds to animals (n=5) belonging to the triple depleted group inoculated with HTLV-1A/C_{oI-L} and light green corresponds to animals (n=10) belonging to the triple depleted group inoculated with HTLV-1A. Each symbol corresponds to an animal of the designated group. The p-values between timepoints within a virus group were calculated by fitting generalized estimating equations with animal as a random effect, while p-values between treatment groups for the same timepoint were calculated by Mann-Whitney/Wilcoxon non-parametric tests. The p-values are shown for all comparisons with $p<0.05$ and are colored according to the direction of significant difference: red, right > left; blue, right < left. Source data are provided as a Source Data file.

Supplementary Fig. 5. Gating Strategy. (a)Representative flow plot showing the gating strategy performed on Blood (left panel) and BAL samples (right panel) from an HTLV-1A infected macaque (17P039) at week 21 post viral inoculation to identify the myeloid cells: (i) three monocyte subsets (classical, intermediate, and non-classical), (ii) dendritic cells (mDCs and pDCs) and (iii) neutrophils (highlighted in red). (b) Representative flow plot showing the fluorescence minus one (FMO) staining on whole blood samples from a naïve, uninfected animals, as control for our strategy.

Supplementary Fig. 6. Cell population and subset differences between triple depleted HTLV-1A/C_{oI-L} and HTLV-1A, highlighting patterns for each cell population between compartments. (a-c) Heatmaps depict the row-scaled frequencies of (a) dendritic cells, subdivided into plasmacytoid (pDCs) and myeloid (mDCs) dendritic cells, (b) total monocytes and subsets ($CD14^+CD16^-$ classical, CL; $CD14^-CD16^+$ non-classical, NC; and $CD14^+CD16^+$ intermediate, IN), and (c) neutrophils including those expressing IL-8, IL-10, TNF-α, CD162 and CD64 in blood (left, yellow) and BAL (right, orange). Only cell populations with $p<0.05$ by Mann-Whitney test between the two triple depleted groups at the timepoint indicated are shown. Populations are sorted

by cytokine produced, then cell subset, then time. Thick black lines separate total cell populations and expression of individual cytokines, while thin black lines separate cell subsets within a group. Timepoint labels are underlaid according to their pattern of significance: HTLV-1A/C_{oI-L} > HTLV-1A (dark green); HTLV-1A/C_{oI-L} < HTLV-1A (light green). Raw non-rowscaled values for each population in this figure are provided as Raw Data and plotted in Figure 3. Source data are provided as a Source Data file.

Supplementary Fig. 7. Alternative visualization of Figure 3. Scatterplots depict cell population frequencies of (a) plasmacytoid dendritic cells (pDCs) and myeloid dendritic cells (mDCs) and their subsets expressing IL-8, IL-10, or TNF-α markers. (b) Total monocytes, with the three monocyte populations ($CD14^+CD16^-$ classical, CL; $CD14^-CD16^+$ non-classical, NC; and $CD14^+CD16^+$ intermediate, IN), and their subsets expressing IL-8, IL-10, both CD162 and IL-10, or TNF-α markers. (c) Neutrophils and their subsets expressing IL-8, IL-10, both CD162 and IL-10, TNF-α, CD11b, CD64, or MPO markers in BAL (top, highlighted in orange) and blood (bottom, highlighted in yellow). Only cell populations with $p<0.05$ by Mann-Whitney test between the two triple depleted groups at weeks 5, 12, or 21 are shown and sorted by timepoint (top to bottom, separated by dotted black lines) then by direction of the HTLV-1A/C_{oI-L} vs HTLV-1A difference (green underlines), and then by increasing p-value. Dark green and teal correspond to animals from the triple depleted group inoculated with HTLV-1A/C_{oI-L} and HTLV-1A respectively. Each symbol corresponds to an animal of the designated group. Source data are provided as a Source Data file.

Supplementary Fig. 8. Distinct cytokine and chemokine profiles in blood and BAL of HTLV-1A/C_{oI-L} and HTLV-1A infected macaques. Scatterplots depict cytokine/chemokine pg/mL at (a) week00, (b) week05, (c) week12 or (d) week21 in BAL (orange) and blood (yellow). Only biomarkers with $p<0.05$ by Mann-Whitney test between the two triple depleted groups at the indicated timepoints are shown and sorted by compartment. Source data are provided as a Source Data file.

Supplementary Fig. 9. Comparable cytokine and chemokine profiles in blood and BAL of all animals inoculated with HTLV-1A/C_{oI-L}. (a-b) Heatmaps depict row scaled pg/mL of significantly different (Mann-Whitney, $p<0.05$) between all groups of animals in Study 1, Study 2 and a prior study[39] inoculated either with HTLV-1A/C_{oI-L} or HTLV-1A at baseline, week 5, 12 and 21 (from top to bottom respectively) in blood (a, yellow) and BAL (b, orange). Significance color bars at periphery of each heatmap designate whether the Mann-Whitney test for the pairwise contrast at top is significantly up/down for the variable in each row. Alluvials connect the same cytokine overtime and between blood and BAL. Source data are provided as a Source Data file

Supplementary Fig. 10. Positive and negative control of the RNAscope and the immunohistochemistry assays. (a) PPIB, expressed in all pulmonary cell types, was labeled with Mmu-PPIB RNAscope probe (yellow) and was used as positive control to test RNA quality within the lung tissue sections. (b) Panel depicts dapB staining labeled with dapB RNAscope probe (yellow) and used as negative control. DAPI was used for nuclear counterstain. Scale bars 50 µm.

(c) Panels of images showing single-antibody IHC for CD20, CD3, Iba1, and SMA as well as the H&E stain performed on the same section. From top to bottom, panels represent sections of lung of an uninfected rhesus macaque: DG8Z left cranial/caudal lobe; TiT left caudal lobe. Spleen of an uninfected rhesus macaque was used as positive control for staining or stained with the isotype control. In H&E-stain of DG8Z lobe, (*) indicates vessels with increased rolling lymphocytes along the tunica intima. Scale bars 200 μm.

Supplementary Fig. 11. HTLV-1C *rex-orf-I* mRNA in 729.6 infected cells. Total RNA isolated from stably infected 729.6 lymphoblastic B-cell line producers of either HTLV-1A, HTLV-1A/C_{oI-L}, or parental uninfected 729.6 B-cells were subject to an RT-PCR using the specific primers depicted in the presence (+) or absence (-) of the reverse transcriptase (RT). (a) The primers used to detect HTLV-1 viral mRNA species were designed either on the exon sequence of the un-spliced mRNAs (*gag-pro-pol, usHBZ,* and *β-actin*) or boundary spanning primers overlapping the splice sites of the singly (*env*, *orf-II*, *p21^rex^*, and *sHBZ*) and doubly spliced (*tax/rex* and *rex-orf-I*) mRNAs. The splice donor (open triangles) and splice acceptor (closed triangles) sites and their positions are indicated below the HTLV-1 provirus genome scheme. Arrows indicate the position of the forward and reverse primers with nucleotide numbering starting at the beginning of the R region for the positive-sense transcripts. Black solid lines designate the region amplified by PCR, and dotted lines designate the introns. (b) The PCR products were separated on a 1% agarose gel and visualized by ethidium bromide staining showing from top to bottom *gag-pro-pol*, *env*, *tax-orf-II*, *orf-II*, *p21*^{rex}, *tax/rex*, *orf-IC, orf-IA*, *rex-orf-I, usHBZ-1*C, *sHBZ-1*A, *sHBZ-1*C and the *β-actin* used as control. (c) Amino acid alignment between the *rex-orf-I* type C and type A putative protein (p16). Functional and structural domains required for p12/p8 (Type A) activity as well as for Rex localization were highlighted starting with the nuclear localization signal of *rex-orf-I* (NLS, gray-blue) at the N-terminus followed by the endoplasmic reticulum retention domain (green), the IL-2 receptor binding domain (purple) and the Calcineurin binding motif (orange) at the C-terminus. The proteolytic cleavage sites between positions 9 and 10, and between 29 and 30 in the p12 protein sequences are represented with red inverted triangles. The multi-alignments were performed with the Clustal O (1.2.4) program using default parameters. Asterisks (*), colons (:), periods (.), and spaces ( ) indicate that the amino acids at a given position are respectively identical, conserved, semi-conserved or different between the two sequences. (d) Schematic representation of HTLV-1A/C_{E-L} chimeric molecular clone with the restriction sites used in the construction and verification of the HTLV-1A/C_{E-L} molecular clone are shown below. Lowercase letters indicate pAB_HTLV-1A backbone DNA sequence and uppercase letters indicate the inserted DNA fragment cassette including a portion of the polymerase gene, and the entire 3' end region of the HTLV-1C spanning the envelop to the 3'LTR. (e) HTLV-1A/C_{E-L} clones were screened using NheI plus KpnI. The left panel is an image indicating the expected restriction digest fragments separated by electrophoresis on a 0.8% agarose gel. The right panel is the digestion pattern of the HTLV-1A/C_{E-L} clone. (f) Total RNA isolated from stably infected 729.6 lymphoblastic B-cell line producers of either HTLV-1A/C_{oI-L}, HTLV-1A/C_{E-L}, or parental uninfected 729.6 B-cells were subject to an RT-PCR using the specific primers for *rex-orf-I* mRNA in the presence (+) or absence (-) of the reverse transcriptase (RT). The PCR products were

separated on a 1% agarose gel and visualized by ethidium bromide staining showing from top to bottom *rex-orf-I* and the *β-actin* used as control. Source data are provided as a Source Data file.

Supplementary Fig. 12. Distinct expression of p16A and p16C produced by *rex-orf-I mRNA* in Jurkat and THP-1 cells. (**a**) Representative western blot analysis of three independent experiment of HA in HeLa cells transfected with the lentiviral particles expressing either p12-HA, p16A-HA, p16C-HA or with the empty vector lentiviral particle used as control (GFP). GAPDH was used as loading control for cell lysates. (b) *Rex-orf-I* mRNA expression normalized to *RNAseP* mRNA expression assessed by qPCR in the THP-1 (left panel) and Jurkat (right panel) stably transduced cell lines. Data are mean ± SD from biological replicates. Representative western blot analysis of three independent experiments of HA, GFP in (c) THP-1 and (d) Jurkat cell lines stably transduced with the same lentiviral vectors mentioned above. GFP and actin were used as loading controls for cell lysates. (e, f, g) Quantification of the distribution of the HA tagged proteins in the nucleus and the cytoplasm of HeLa cells (100 cells) transfected with the lentiviral particles expressing (e) p12-HA, (f) p16A-HA, or (g) p16C-HA. These data are representative of three independent experiments. Significance: * $p < 0.05$ and ** $p < 0.01$. (h) Representative images showing from top to bottom localization of p16A in HeLa cells transfected with p16A-HA of p12/p8-HA and p16C-HA lentiviral vectors. 48h post transfection, cells were fixed and immune-stained with antibodies against HA (red), GFP (green) and the nuclei contrasted with DAPI (blue) and analyzed by fluorescence microscopy. Scale bar 10 µm. Source data are provided as a Source Data file.

Supplementary Fig. 13. p16C encoded by *rex-orf-I* doubly spliced mRNA inhibits efferocytosis. (a,b) Percent uptake of apoptotic Jurkat cell lines stably transduced with p16C-HA or p12-HA by (a) primary $CD14^+$ cells, or (b) monocytic THP-1 cell line quantified by flow cytometry at 2, 4, 8, 12, 18 and 24 hours post cocultivation. Data were collected from six independent experiments (n=6 healthy human donors) run in triplicate for the CD14+ cells and from two independent experiment run in triplicate for the THP-1 cell lines. Comparisons were performed using (a,b) two-tailed Mann–Whitney U test and median. (a,b) Graphs show mean ± SD using Wilcoxon test. (c) Expression of IL-10 in THP-1 cell line stably transduced with (a) p12/p8-HA (n=8) or (b) p16C-HA (n=5) GFP-expressing lentiviral vectors. 48 hours post PMA stimulation, culture supernatants were collected and assessed for cytokine expression using PEA. Source data are provided as a Source Data file.

Supplementary Table 1. Animal prior history. The table lists the history of the fourteen concurrent animals in addition to the five historic animals used in this study, including animal ID, source, vaccination protocols, virus strains, route of virus exposure, sex, age at the initiation of the study as well as PCR status before enrollment in the current study. As indicated by superscript, ([1]) $gp120_{\Delta V1}$ = deletion of V1 in SIV/HIV gp120 envelope protein[60], ([2]) ALFQA = Army Liposome Formulation Containing QS21 saponin absorbed to aluminum hydroxide[61], and ([3]) SAMT-247 = S-acyl-2-mercaptobenzamide thioester. Source data are provided as a Source Data file.

Supplementary Table 2. Detailed histopathological observation in the lung lobes of the infected macaques. (a,b) From top to bottom, seven lung lobes (right cranial, right middle, right caudal, accessory, left cranial/cranial part, left cranial/caudal part and left caudal), were obtained at euthanasia (48 weeks post viral inoculation) from (a) three infected animals from Study 1 either triple depleted for $CD8^{+}$T, NK, and monocytes (DG8Z, TMN) or not depleted (TiT) prior to HTLV-1A/$C_{oI\text{-}L}$ viral inoculation and from (b) three infected animals from Study 2 (TRE, RH5 and RKF) triple depleted for $CD8^{+}$T, NK, and monocytes prior to HTLV-1A viral inoculation. Source data are provided as a Source Data file.

Supplementary Table 3. HTLV-1A/$C_{oI\text{-}L}$ viral expression and histopathology diagnosis in the lung lobes of the infected macaques. (a,b) From top to bottom, seven lung lobes (right cranial, right middle, right caudal, accessory, left cranial/cranial part, left cranial/caudal part and left caudal), were obtained at euthanasia (48 weeks post viral inoculation) from (a) three infected animals from Study 2 triple depleted for $CD8^{+}$T, NK, and monocytes prior to HTLV-1A viral inoculation or from (b) three infected animals from Study 1 either triple depleted for $CD8^{+}$T, NK, and monocytes (DG8Z, TMN) or not depleted (TiT) prior to HTLV-1A/$C_{oI\text{-}L}$ viral inoculation. Each heatmap block depicts, from left to right, the detection (black) or the absence of detection (gray) of DNA gag and orf-I/II region followed by the transcripts of *gag, rex-orf-I*, *tax*, *usHBZ*, and *sHBZ*. The histopathology diagnosis in each lobe is mentioned as (-) for absence of detection of bronchiectasis and/or fibrosis, (+) for detection of bronchiectasis, and (nd) in the lobes where the histopathology assay was not done. Source data are provided as a Source Data file and Supplementary Data file.

Supplementary Table 4. Detection of HTLV-1 *gag* by RNAscope in the lung of infected macaques. A total of seven lung lobes from the three infected animals in addition to an uninfected macaque were stained with HTLV-1 *gag* probe. Source data are provided as a Source Data file.

Supplementary Table 5. Primers and probes. This table describes the list of primers and probes used for the transcriptional characterization of the HTLV-1A/$C_{oI\text{-}L}$ molecular clone as well as in the detection of the virus in the blood, lymph nodes, Bone Marrow, BAL as well as in the lung lobes of the infected animals. Abbreviations: Fr: Forward, Rv: Reverse, SJ: Splice Junction, bp: base pair. 1st and 2nd indicate when primers were used in the first and/or second round of PCR respectively. (*) Depicts the position of the primers with the nucleotide numbering starting at the beginning of the R region for the positive-sense transcripts. (^) indicates the position of the boundary spanning primers. Source data are provided as a Source Data file.

Supplementary figure 1a

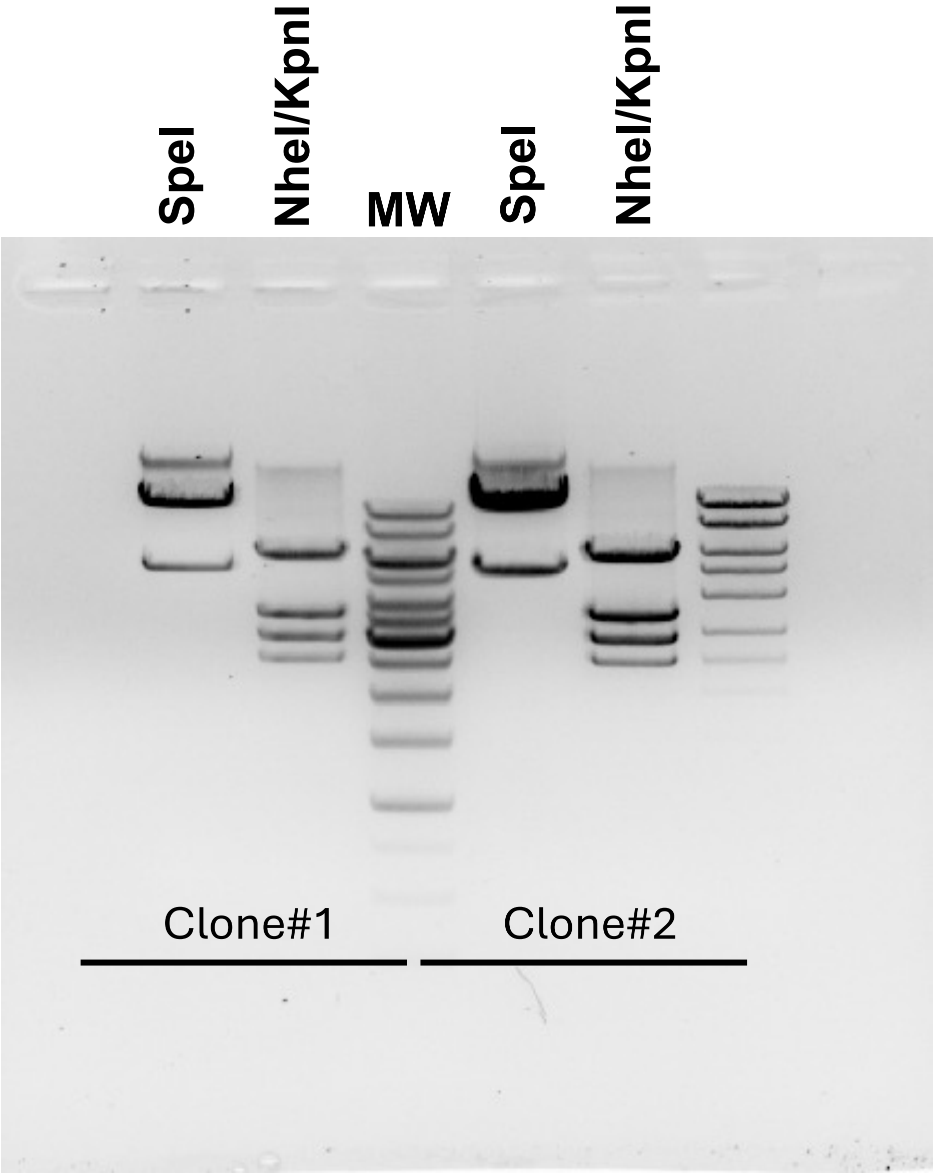

Supplementary figure 1b

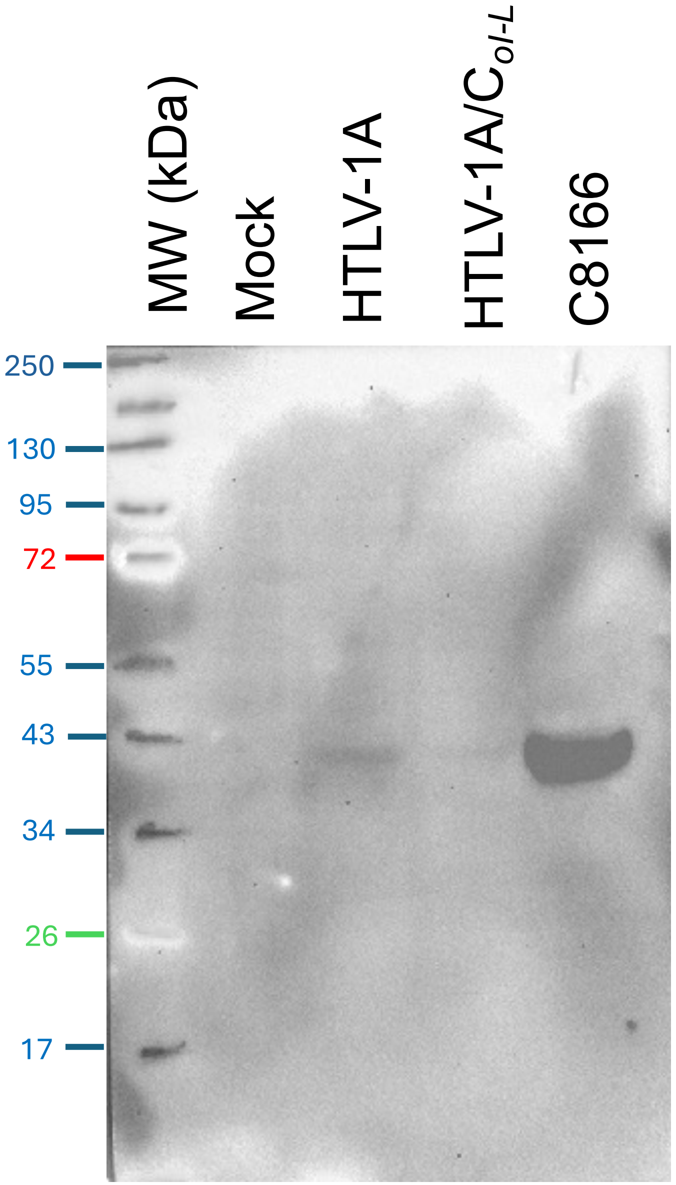

Tax clone LT-4

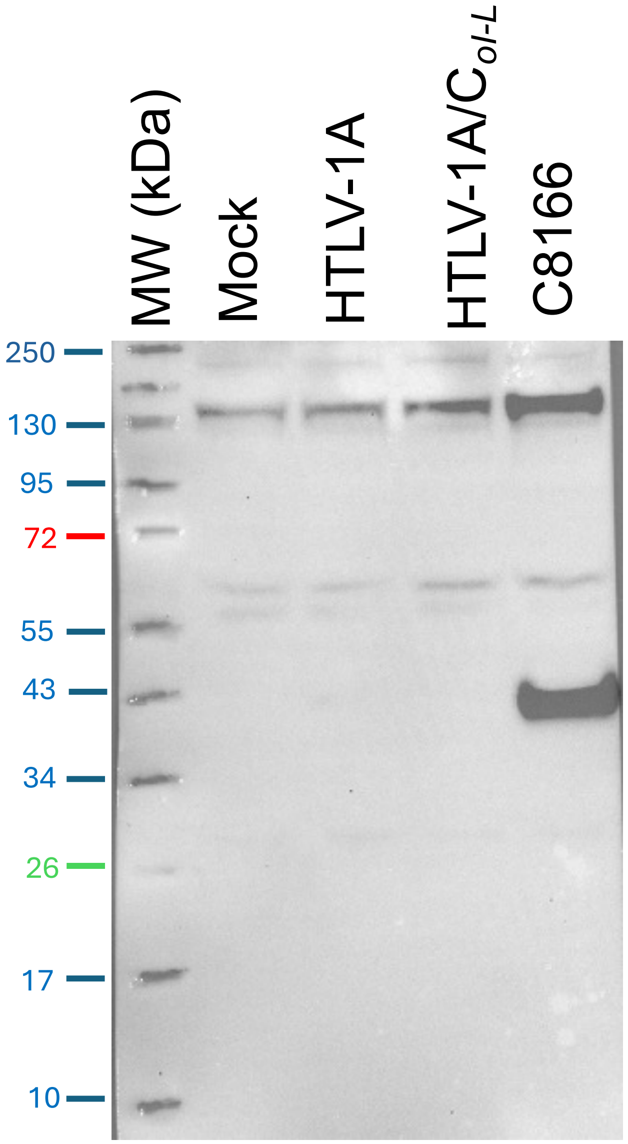

Tax clone 1A3

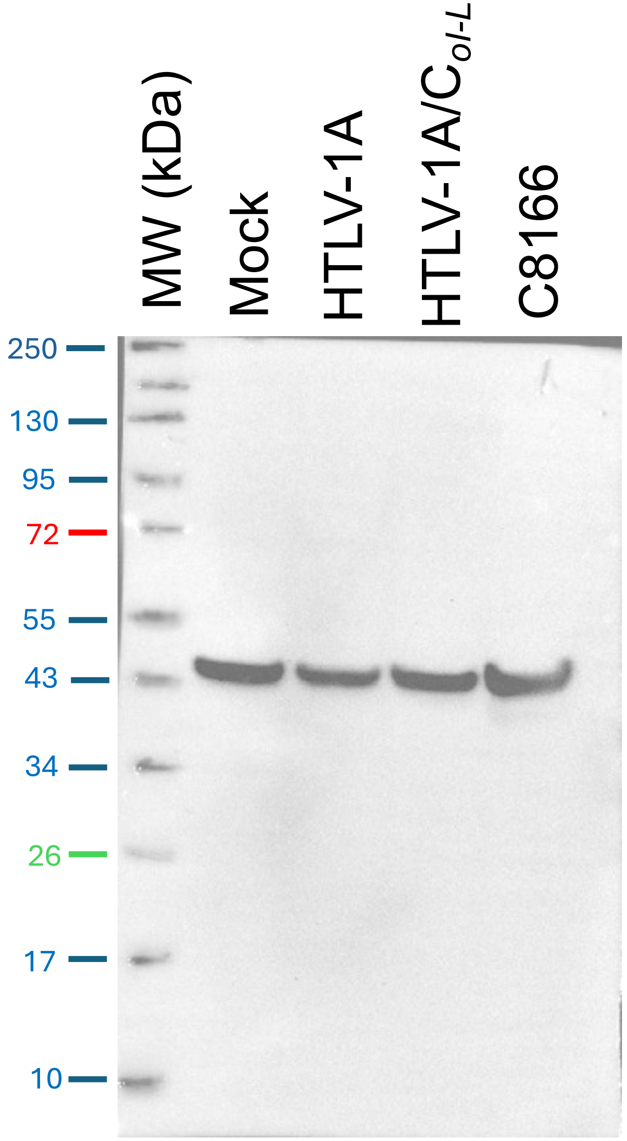

β-actin

Supplementary figures 3a,c,e,f

Raw_Data_Study_1_HTLV-1A/C$_{ol\text{-}L}$

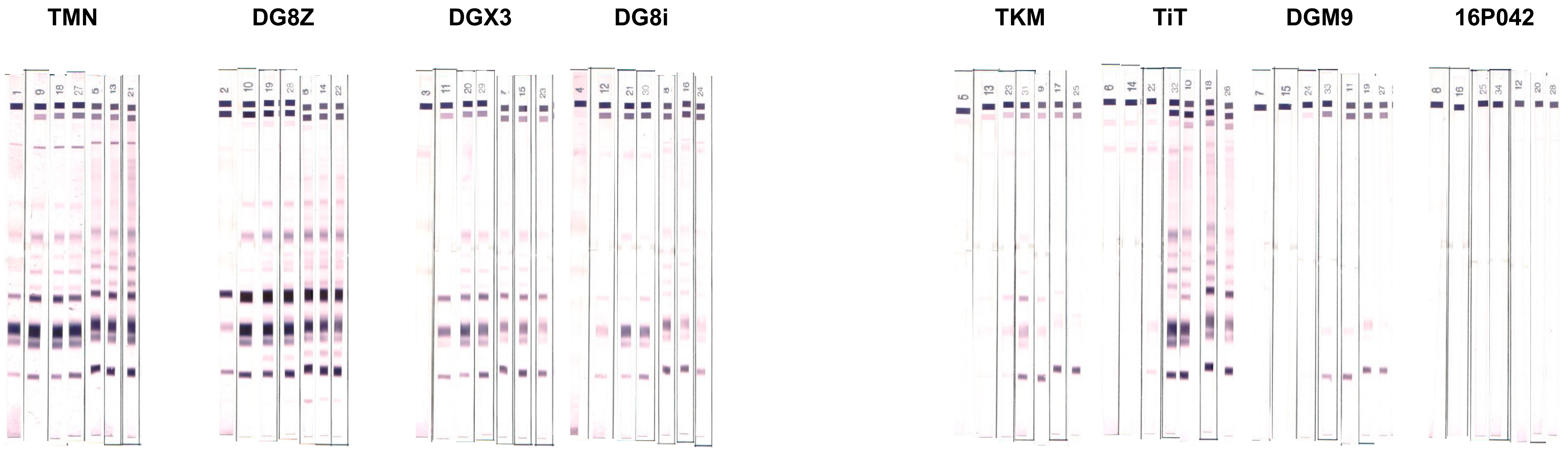

Each strip represents the reactivity to specific HTLV-1 viral antigens in the plasma of the indicated animals
With from left to right at week 3, 5, 7, 10, 12,16, 21

Supplementary figures 3a,c,e,f

Raw_Data_Study_2_both_groups

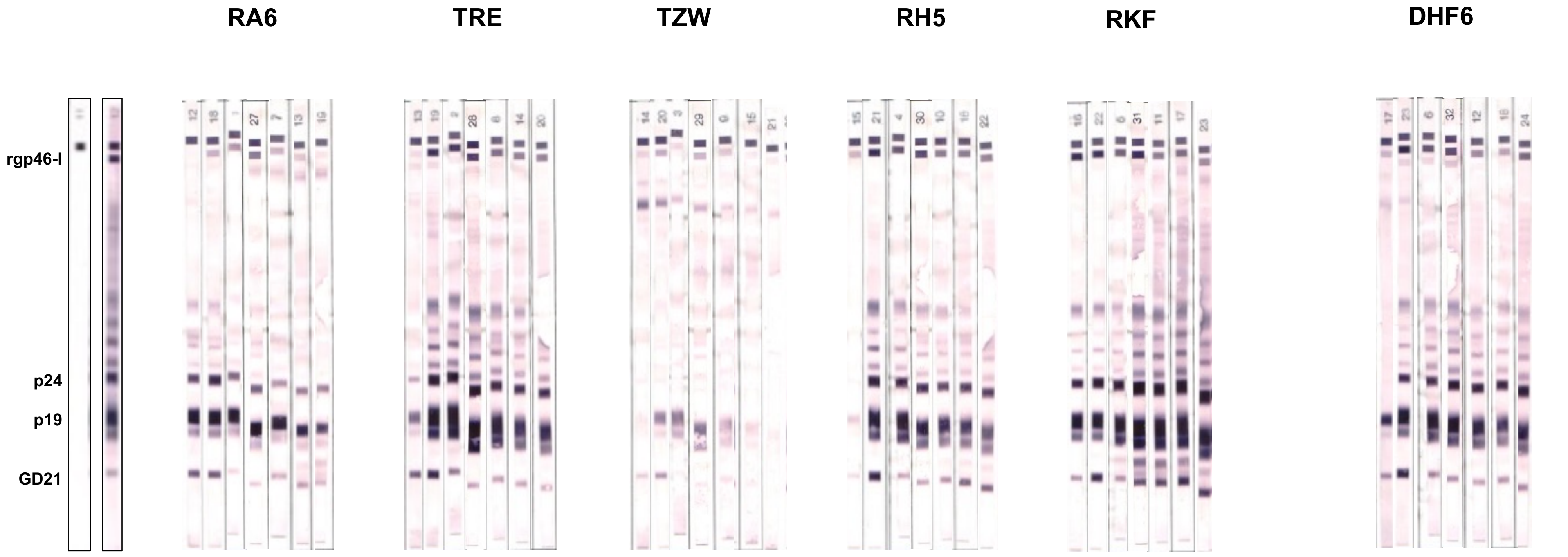

Each strip represents the reactivity to specific HTLV-1 viral antigens in the plasma of the indicated animals
With from left to right at week 3, 5, 7, 10, 12,16, 21

Supplementary figures 3a,c,e,f

Study_1_HTLV-1A/C_{ol-L}_PBMC_Week_03

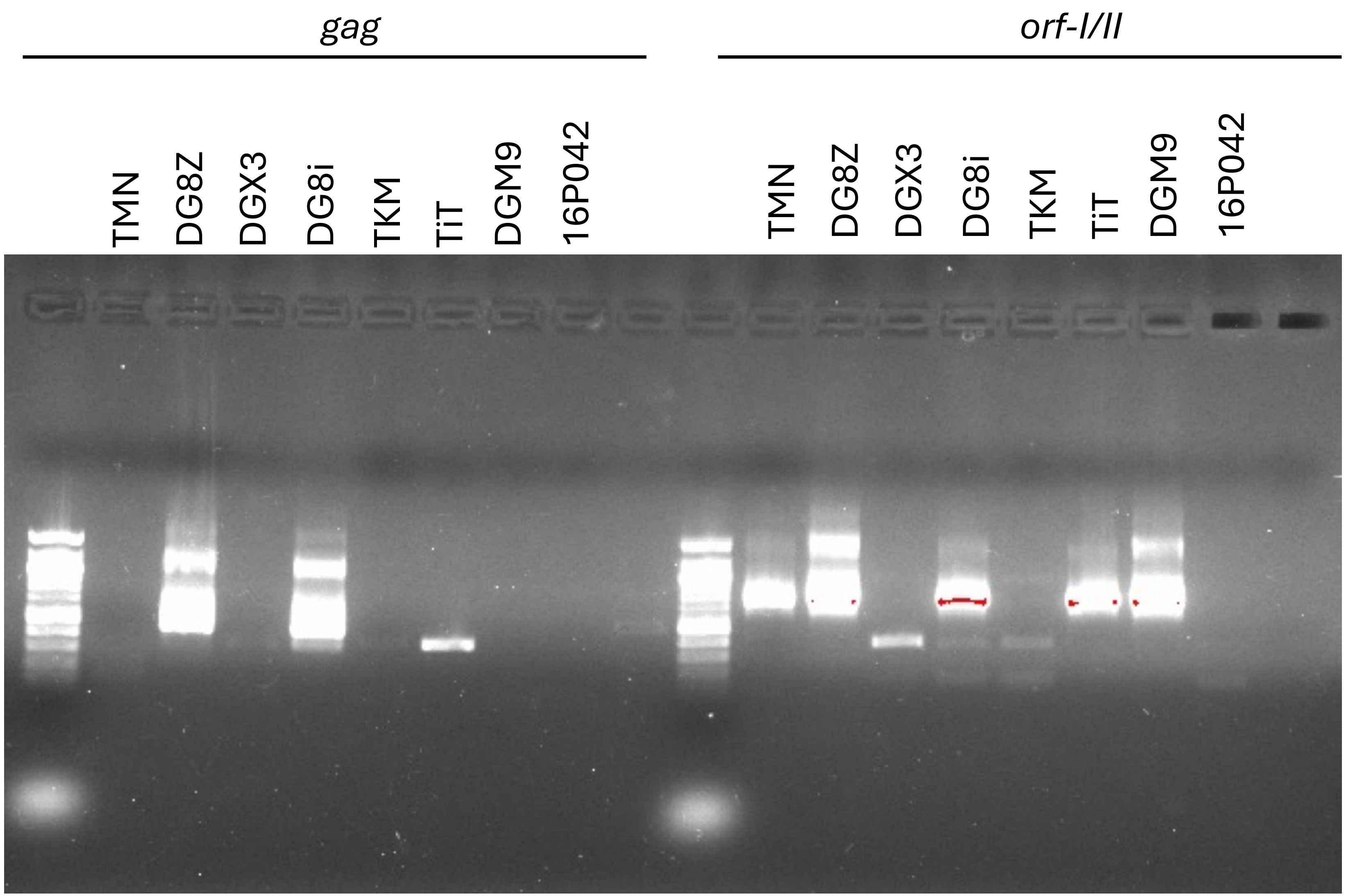

Supplementary figures 3a,c,e,f

Study_1_HTLV-1A/$C_{ol\text{-}L}$ _PBMC_Lymphnode_BAL_Week_05

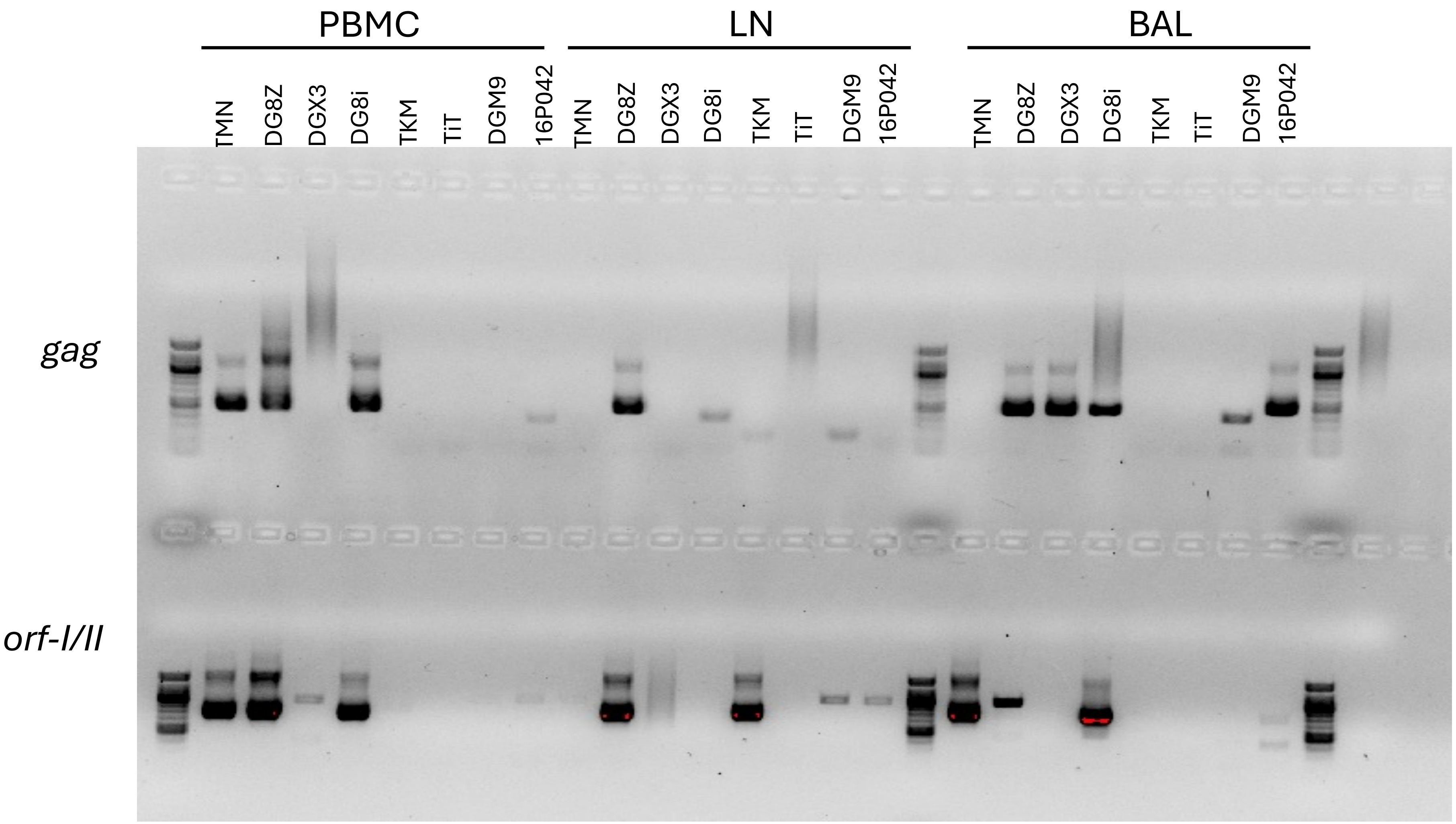

Supplementary figures 3a,c,e,f

Study_1_HTLV-1A/C$_{ol\text{-}L}$ _PBMC_Bone Marrow_Week_07

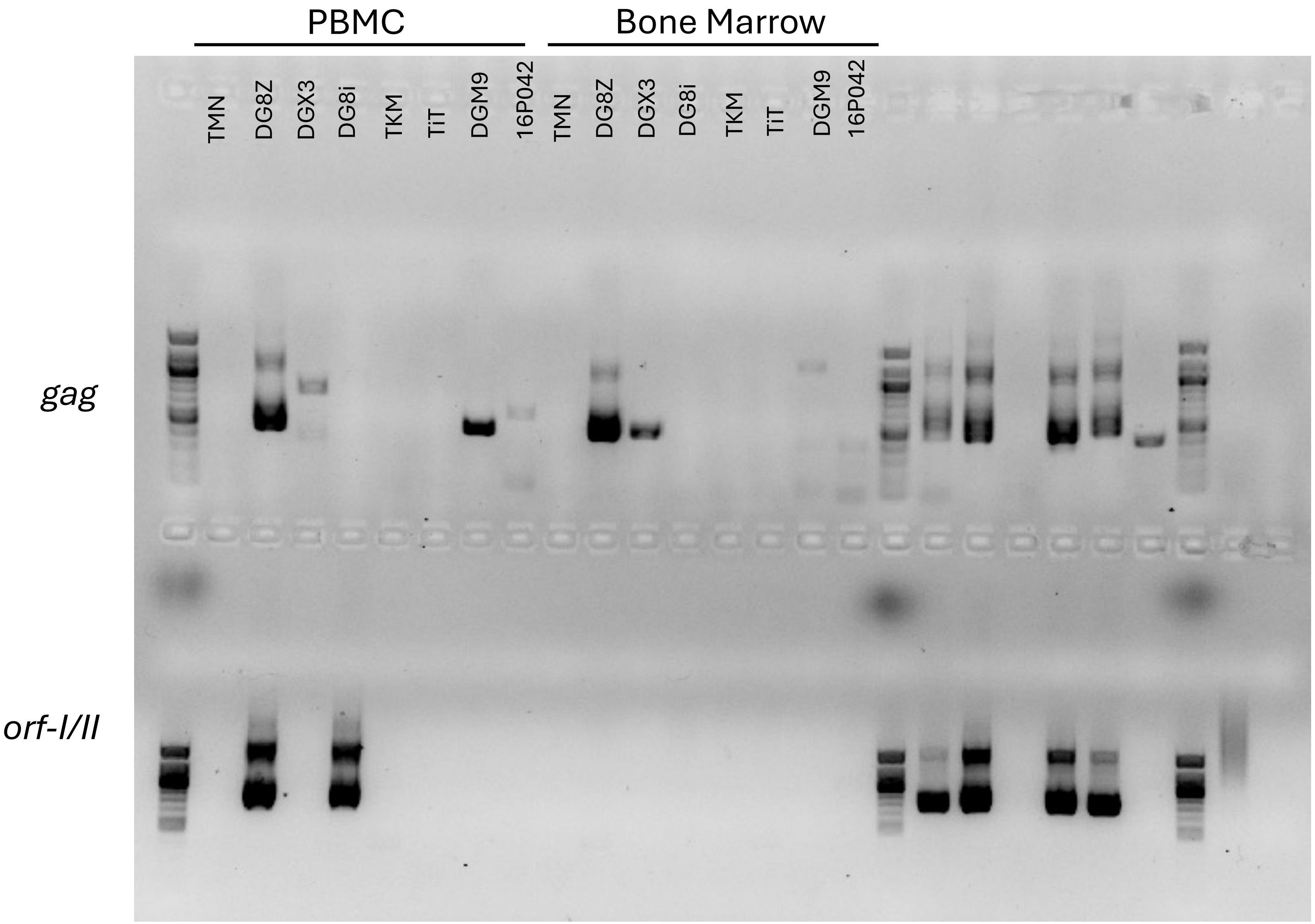

Supplementary figures 3a,c,e,f

Study_1_HTLV-1A/C$_{ol\text{-}L}$ _PBMC_LN_Week_10

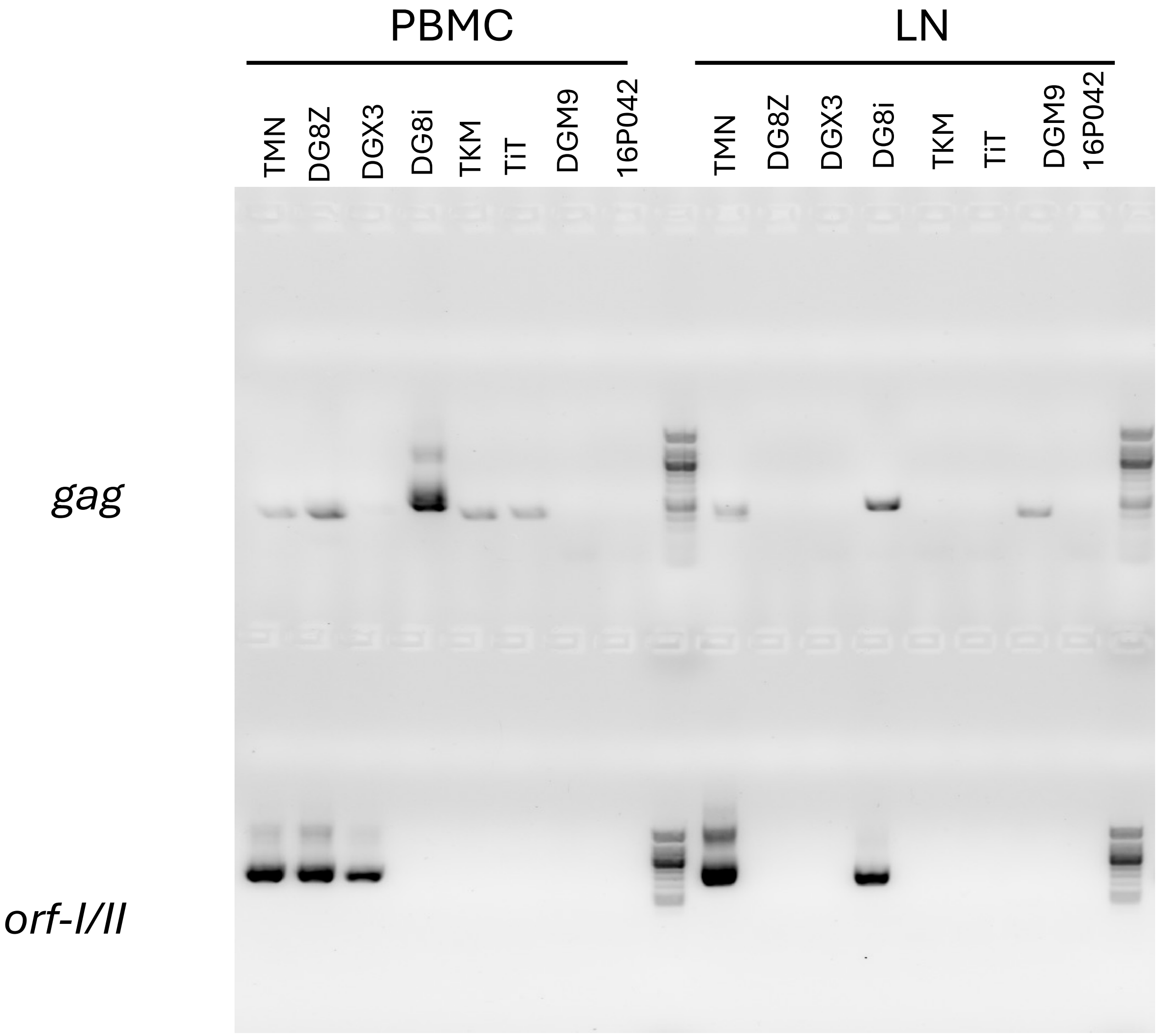

Supplementary figures 3a,c,e,f

Study_1_HTLV-1A/C*ol-L* _PBMC_Week_12

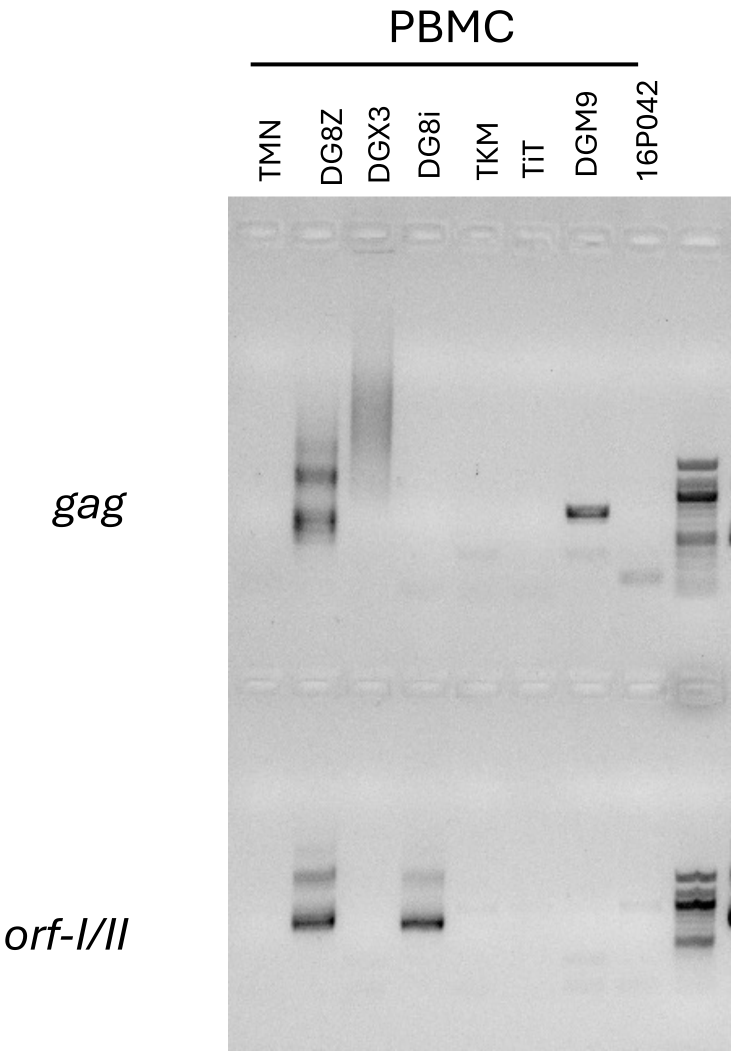

Supplementary figures 3a,c,e,f

Study_1_HTLV-1A/C$_{ol\text{-}L}$ _PBMC_Week_16_Week_21

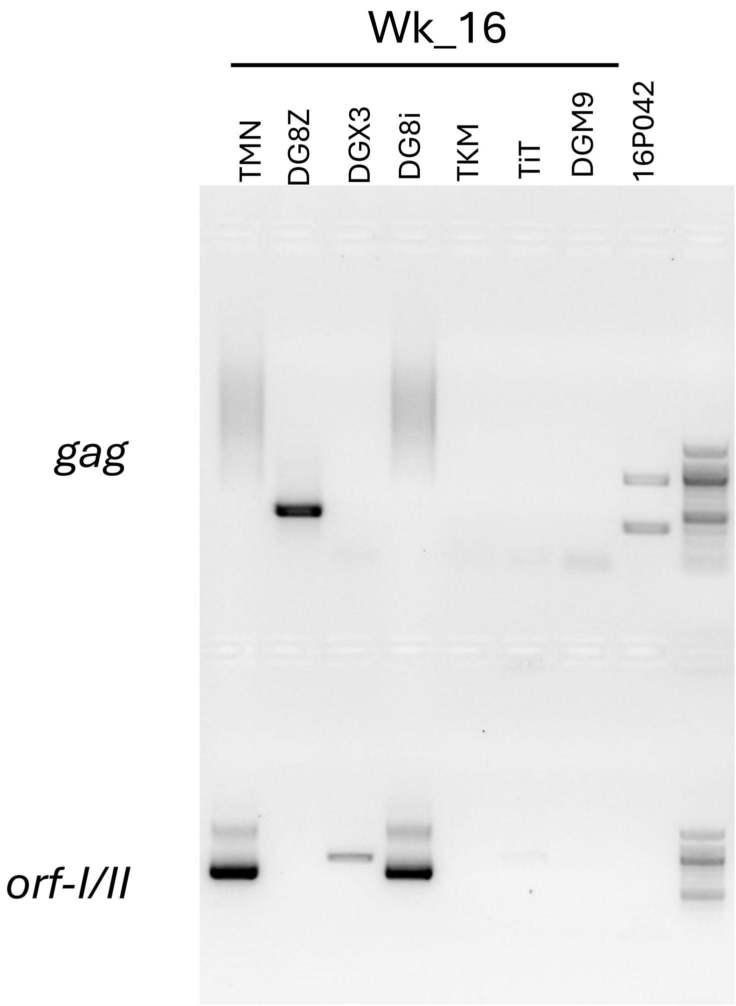

Supplementary figures 3a,c,e,f

Study_1_HTLV-1A/C$_{ol-L}$ _PBMC_BAL_Week_21

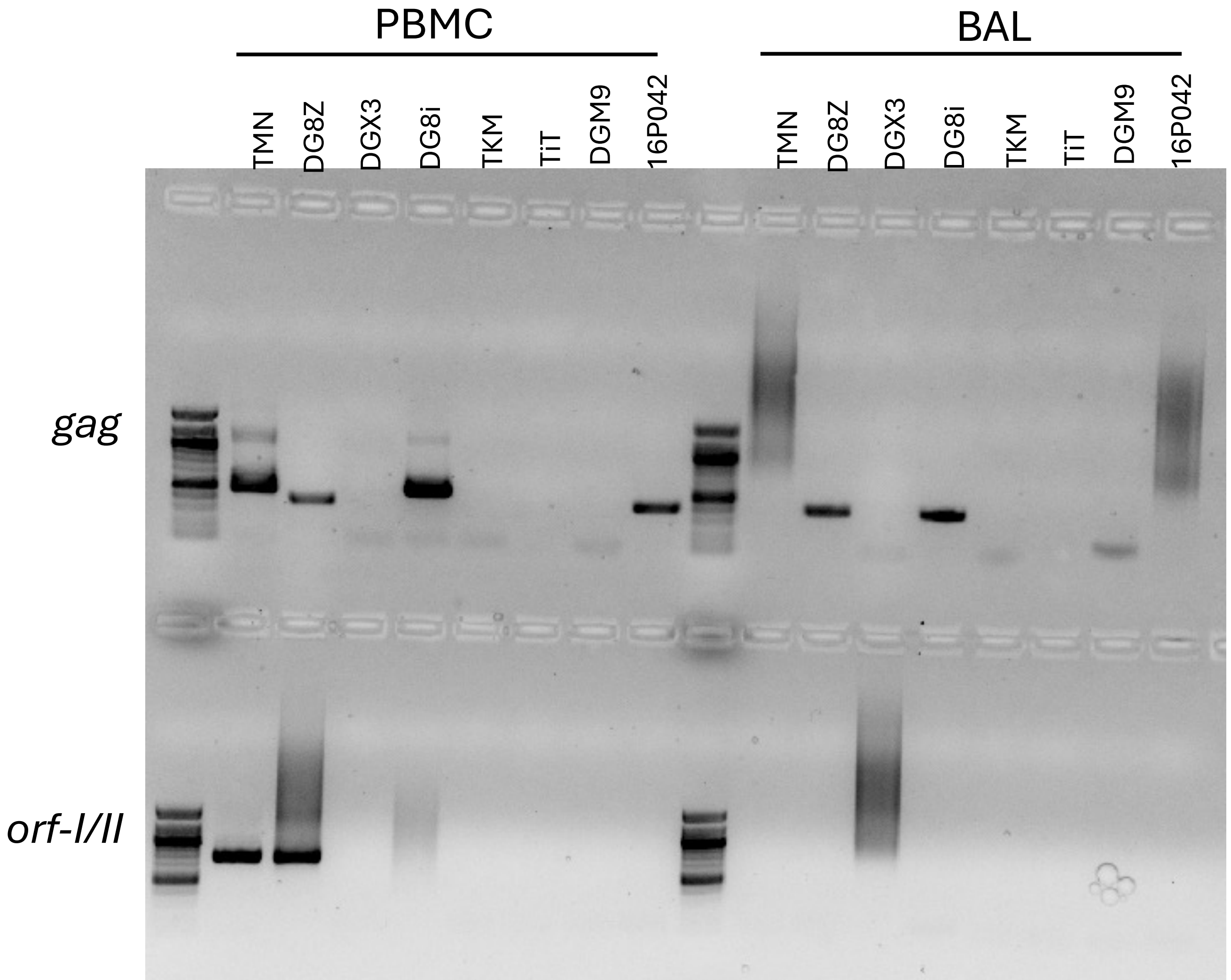

Supplementary figures 3a,c,e,f

Study_2_HTLV-1A_PBMC_Week_03_Week_05_Week_07

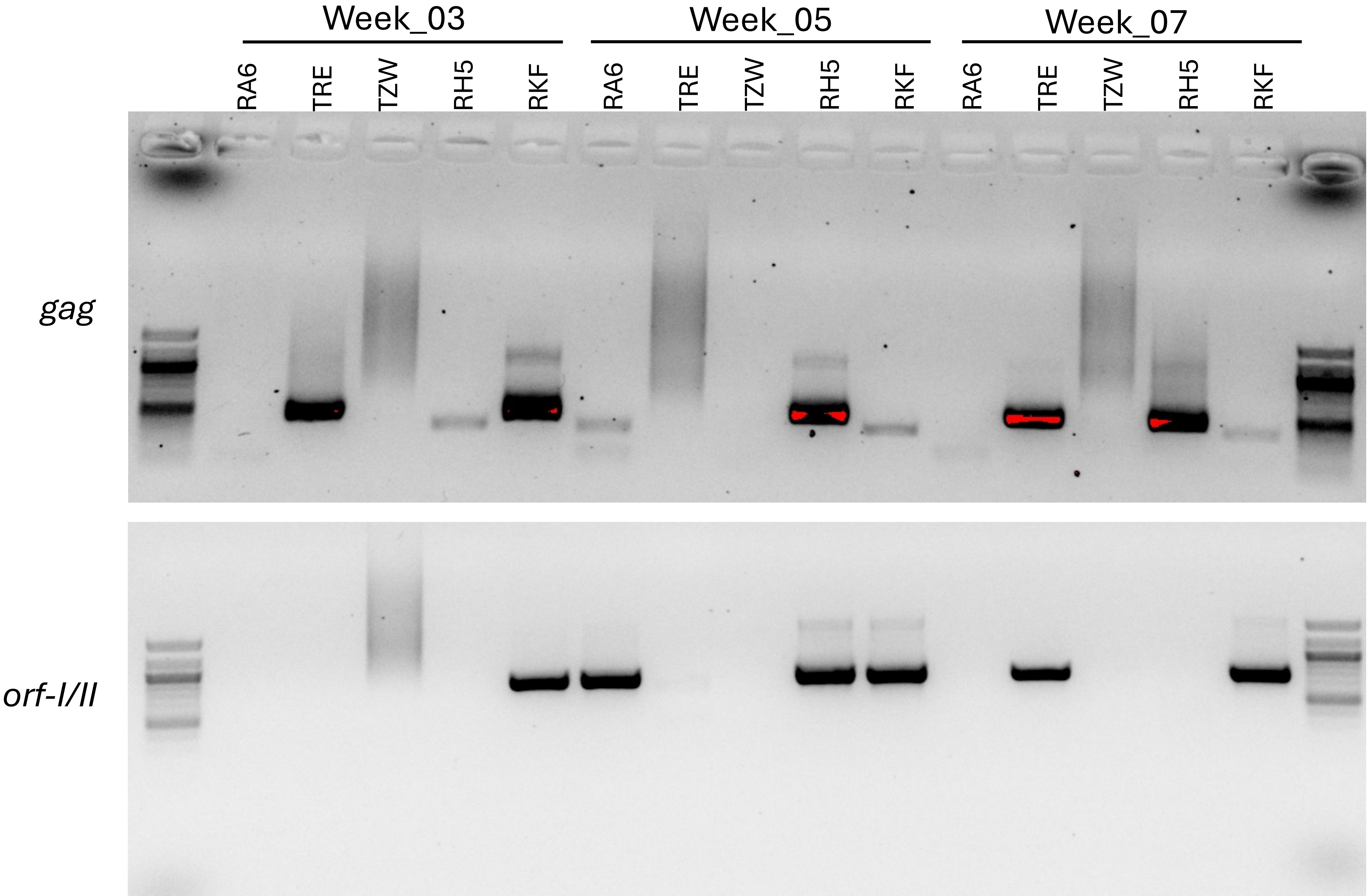

Supplementary figures 3a,c,e,f

Study_2_HTLV-1A_PBMC_Week_10_Week_12

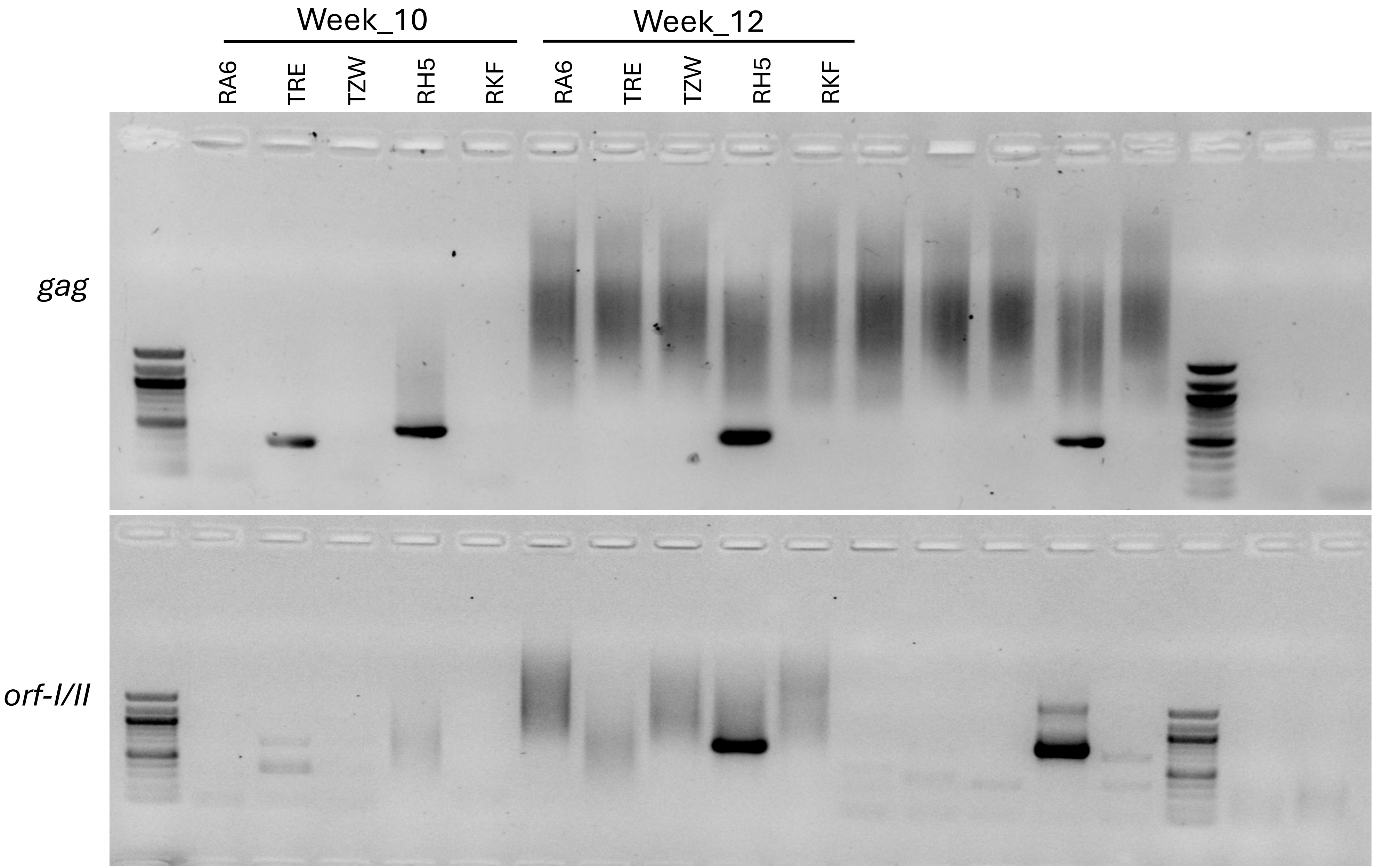

Supplementary figures 3a,c,e,f

Study_2 _HTLV-1A_PBMC_Week_16_Week_21

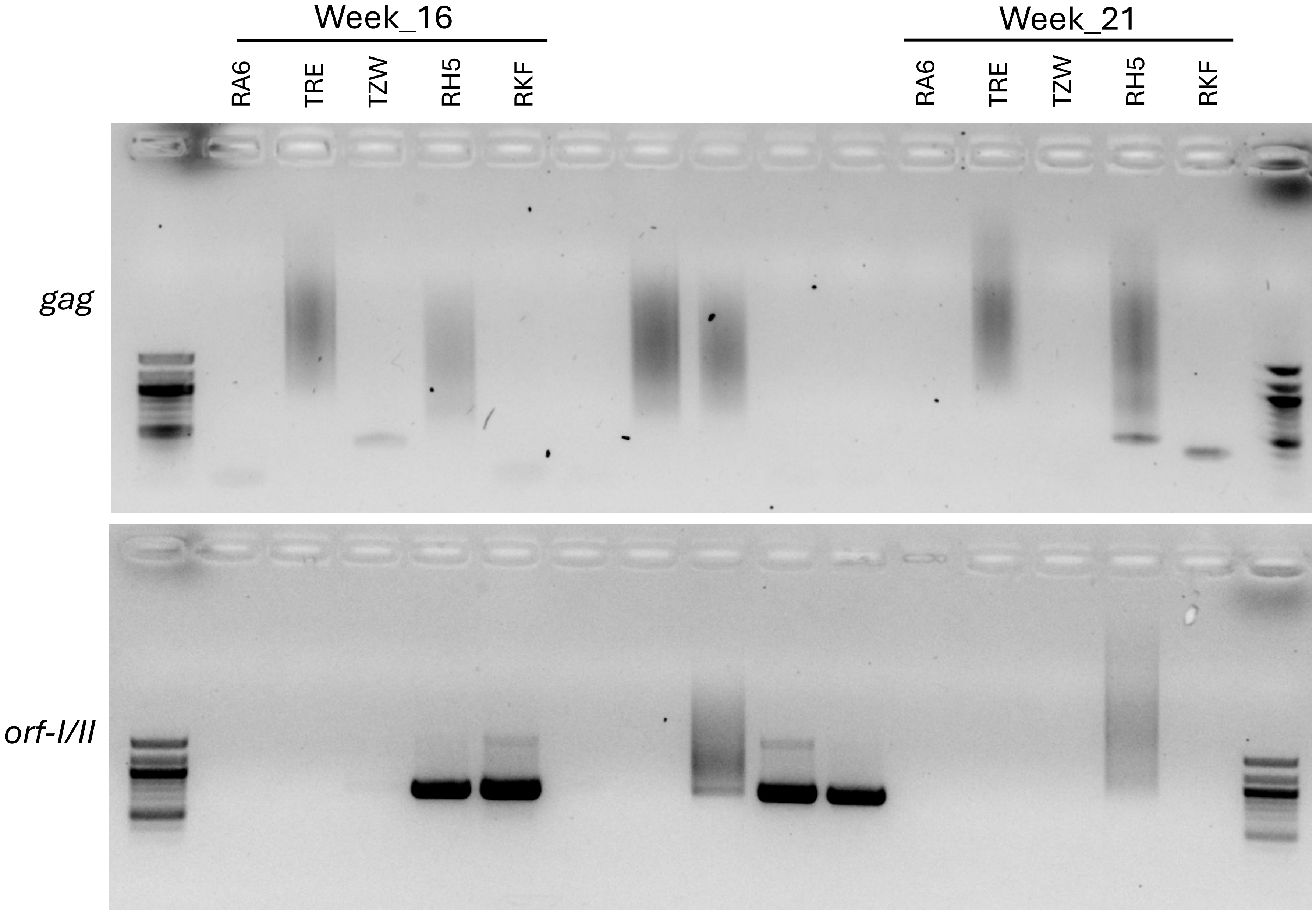

Supplementary figures 3a,c,e,f

Study_2 _HTLV-1A_LN_Week_05_Week_10

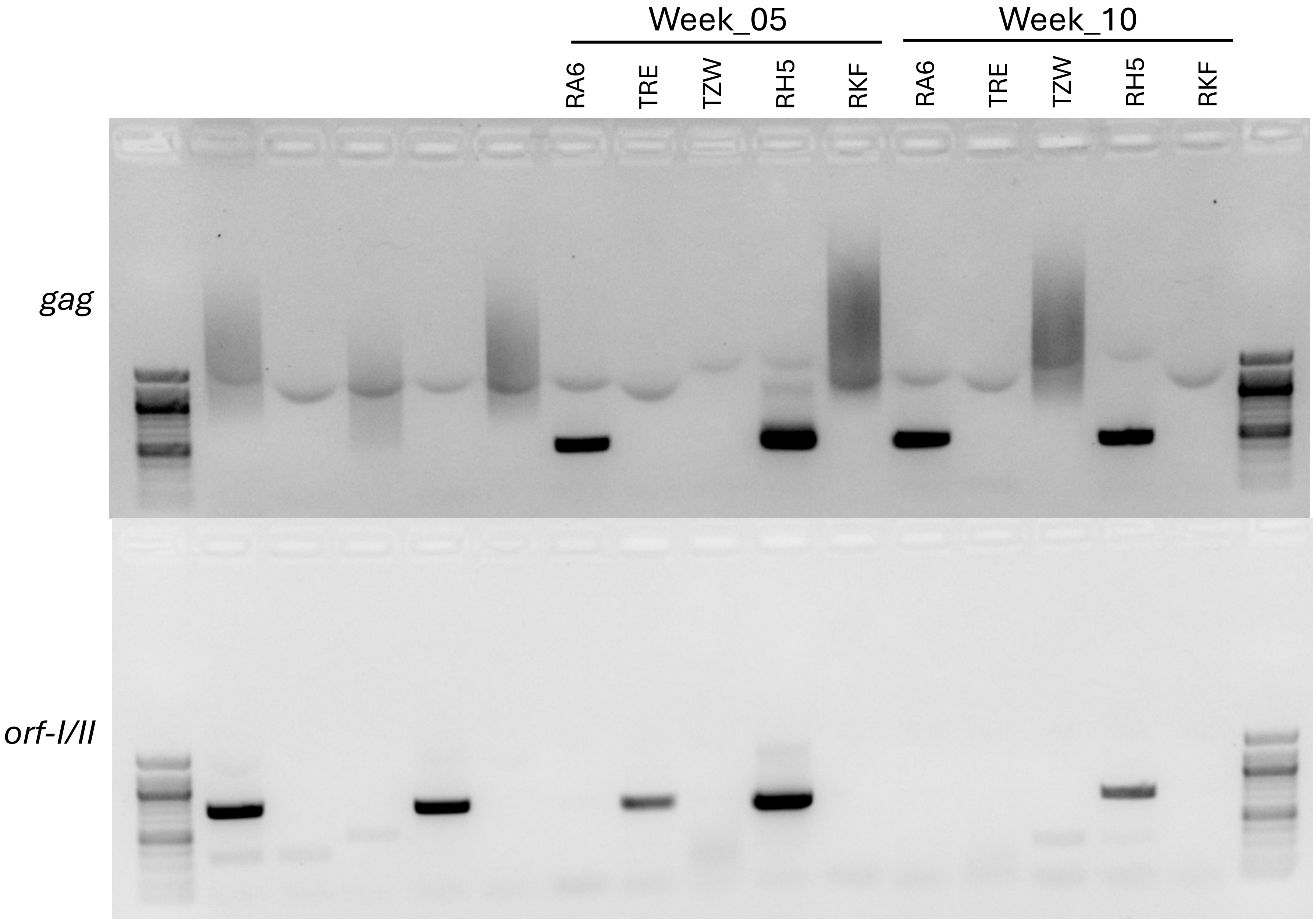

Supplementary figures 3a,c,e,f

Study_2 _HTLV-1A_BM_Week_07

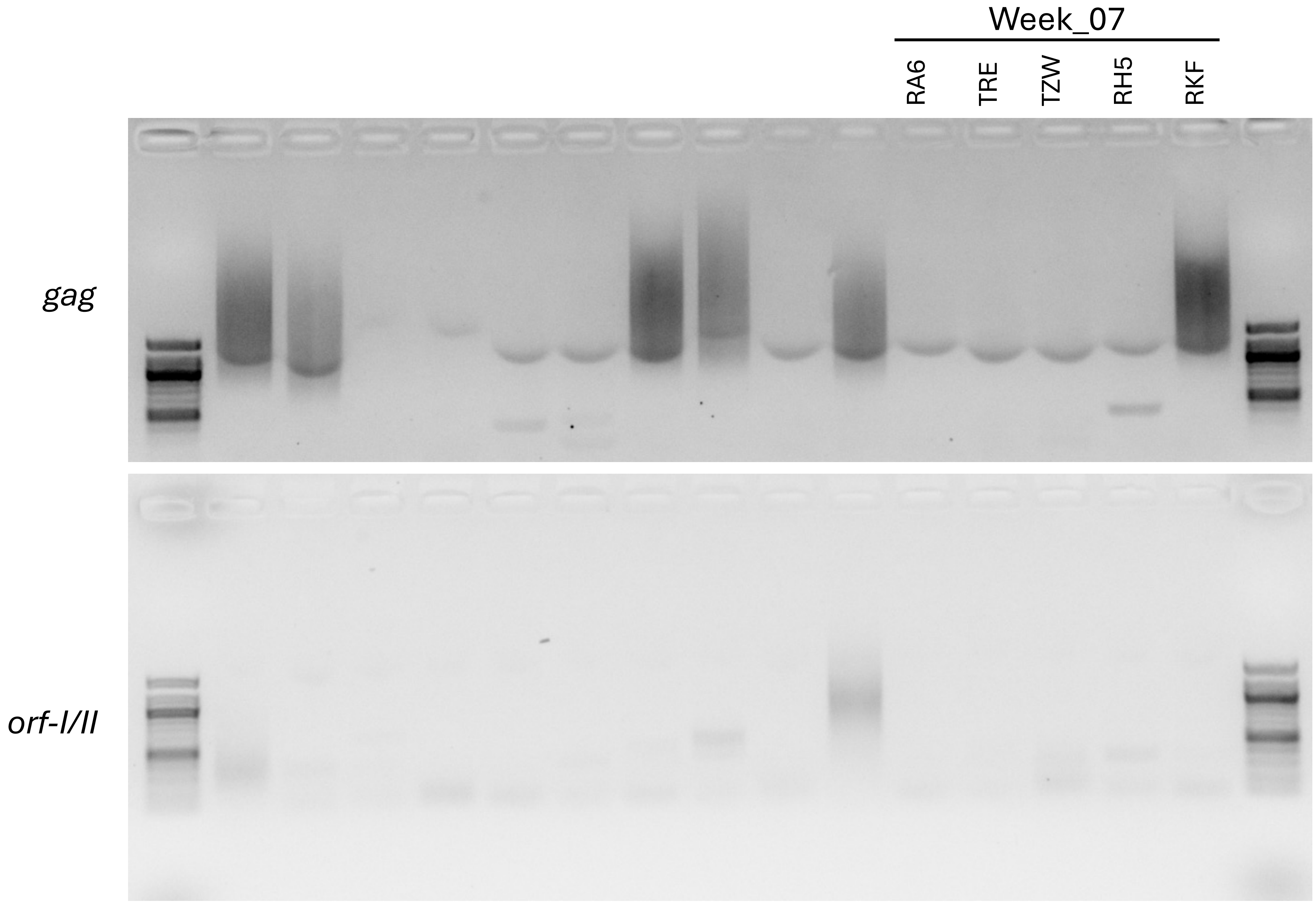

Supplementary figures 3a,c,e,f

Study_2 _HTLV-1A_BM_Week_12_BAL_Week_05

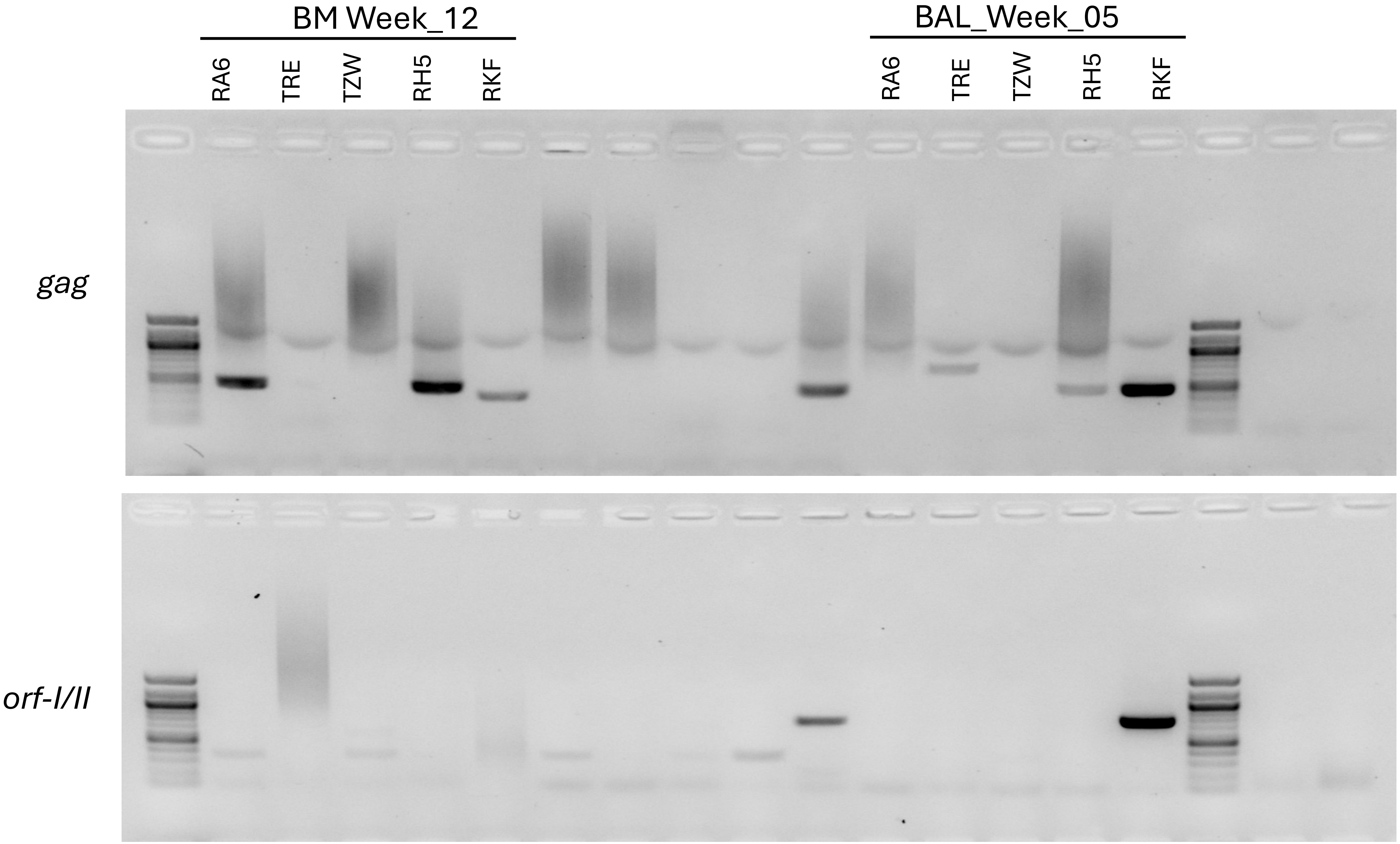

Supplementary figures 3a,c,e,f

Study_2 _HTLV-1A_BAL_Week_12_Week_21

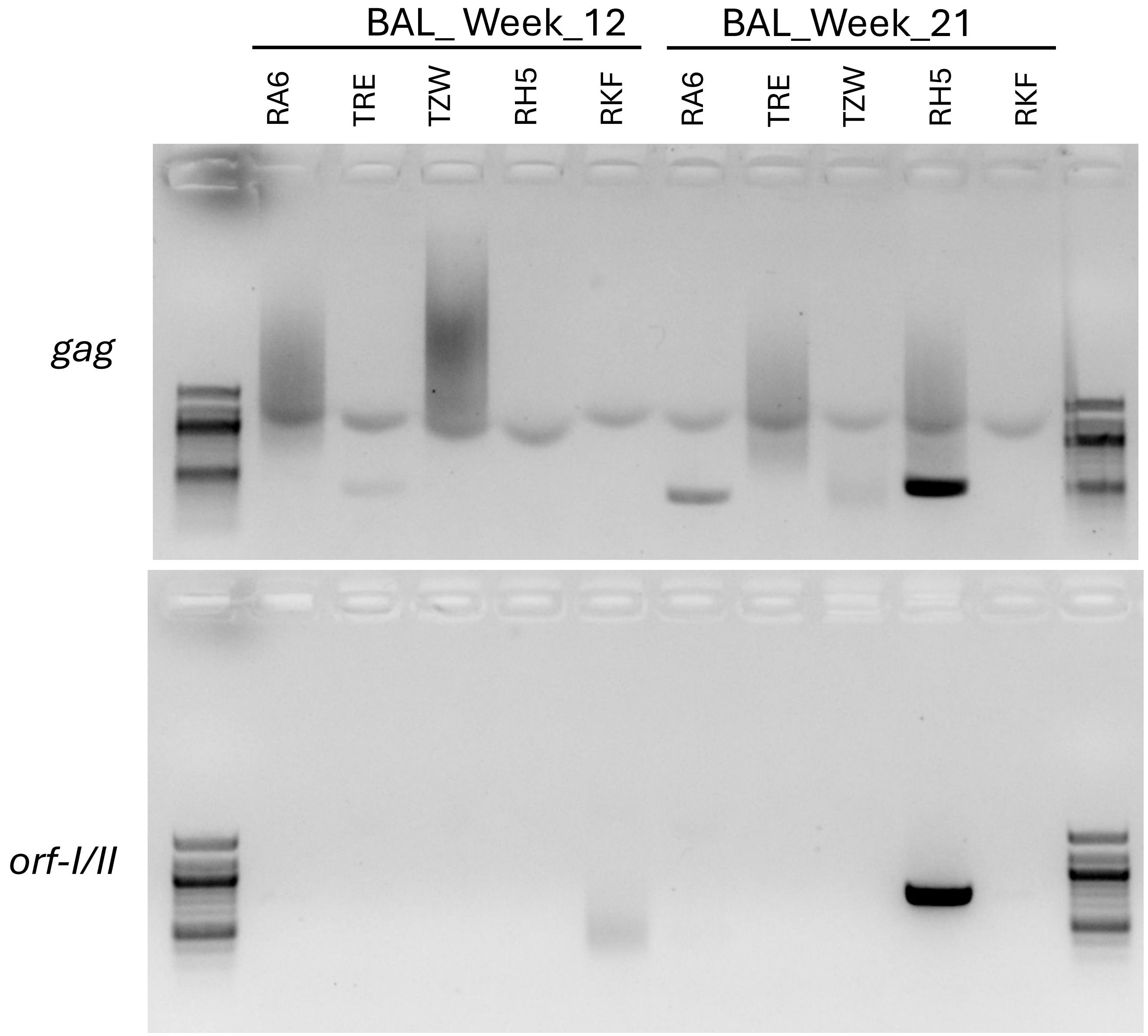

Supplementary figures 3a,c,e,f

Study_2 _HTLV-1A/C_{ol-L}_DHF6_PBMC_LN_BM_BAL_All_timepoint

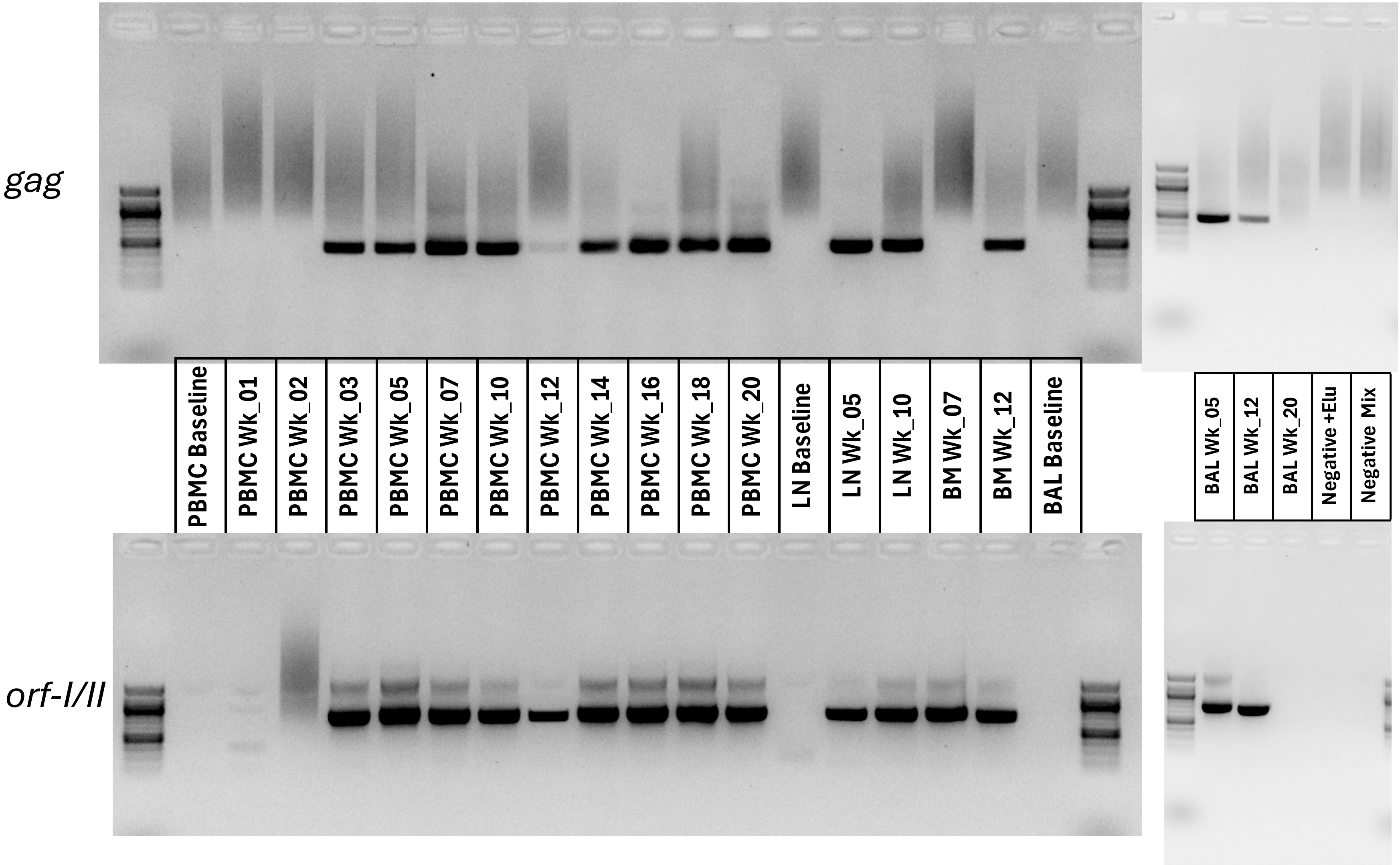

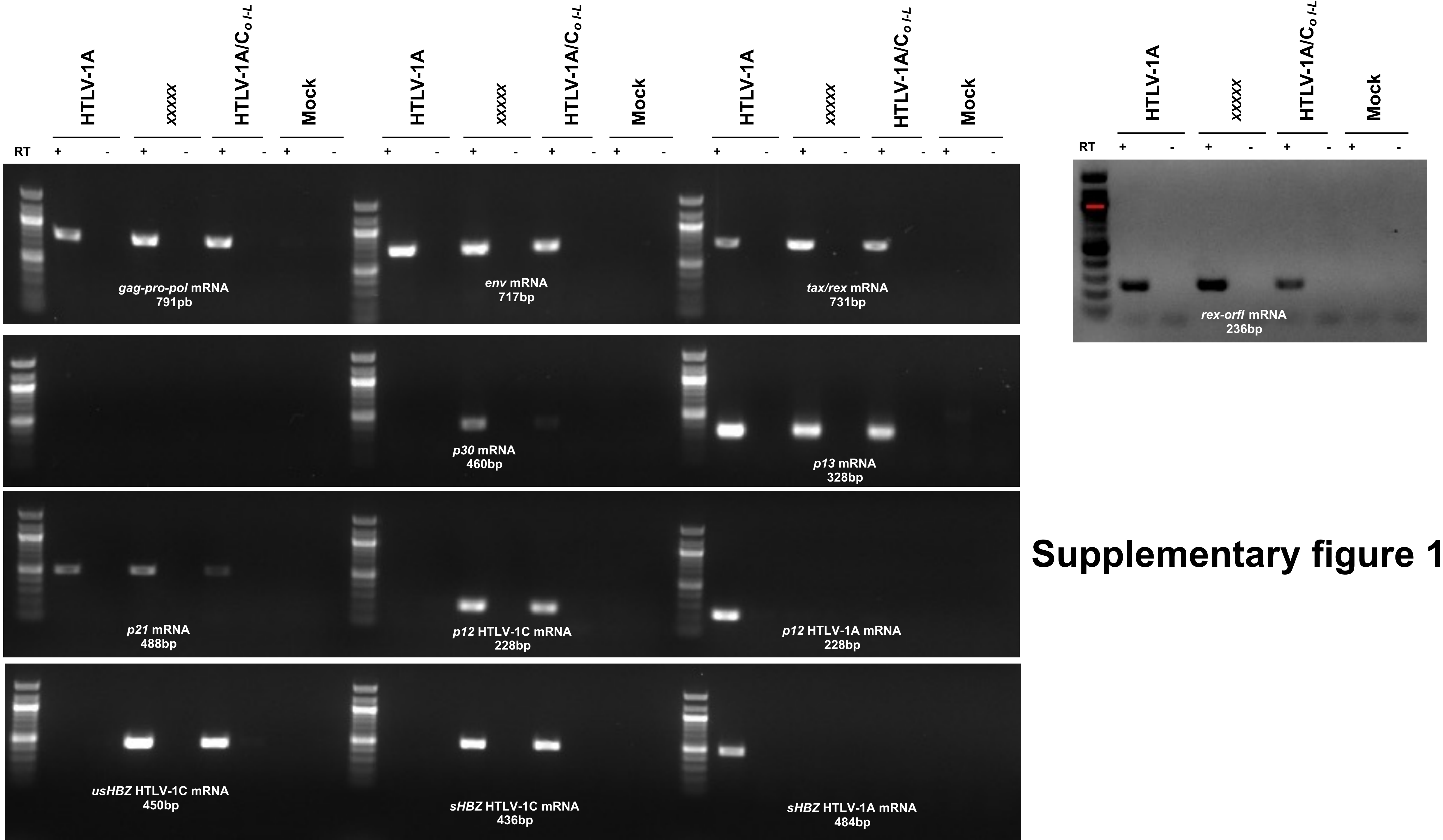

Supplementary figure 11b

Supplementary figure 11e

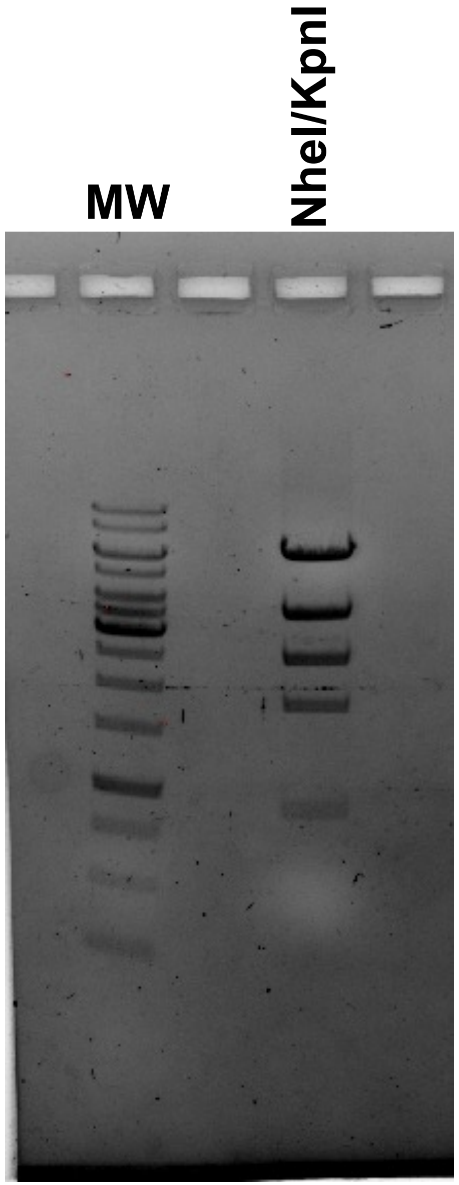

Supplementary figure 11f

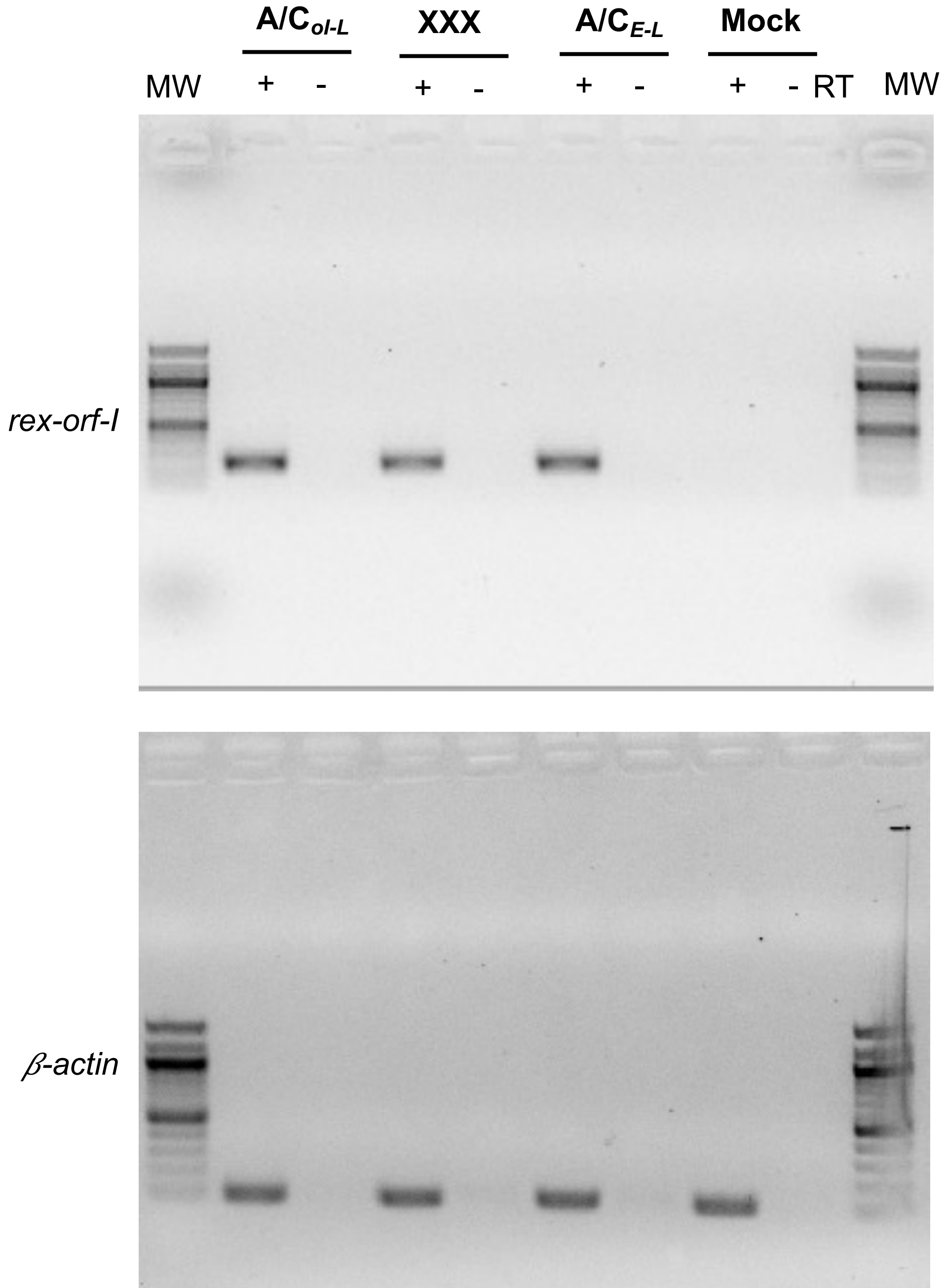

Supplementary figure 12a

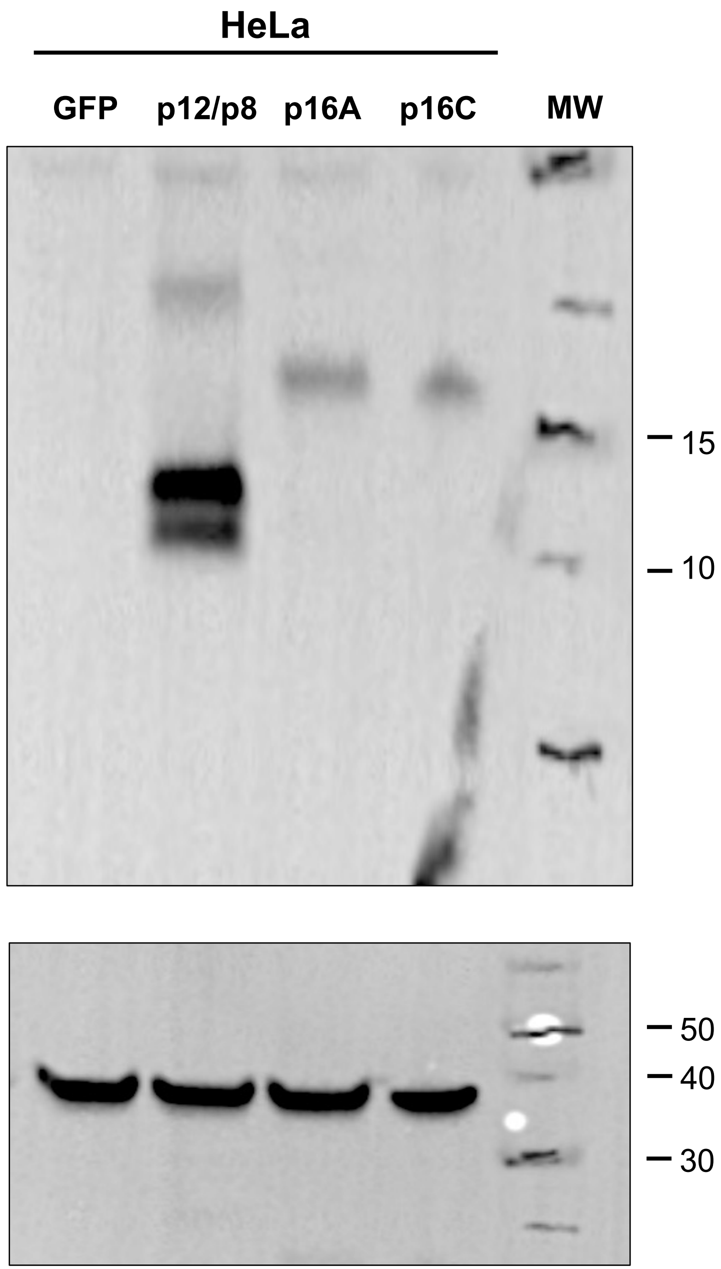

Supplementary figure 12c

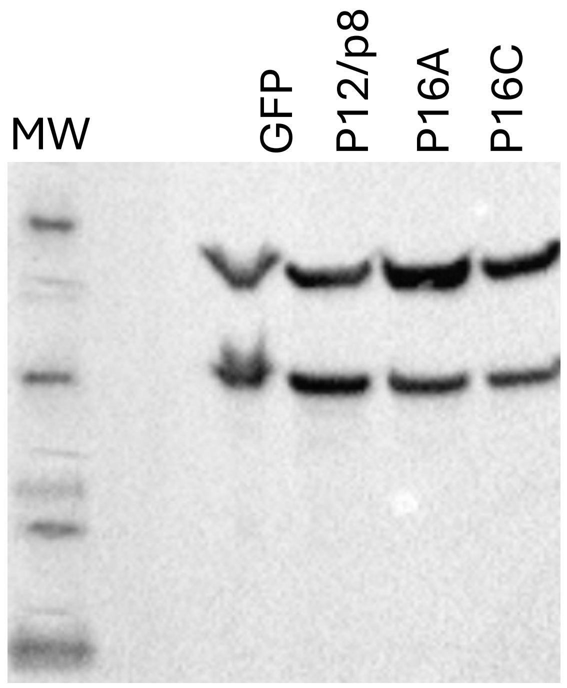

β-actin (Rabbit Cell Signaling Technology, D6A8, 1:1000)

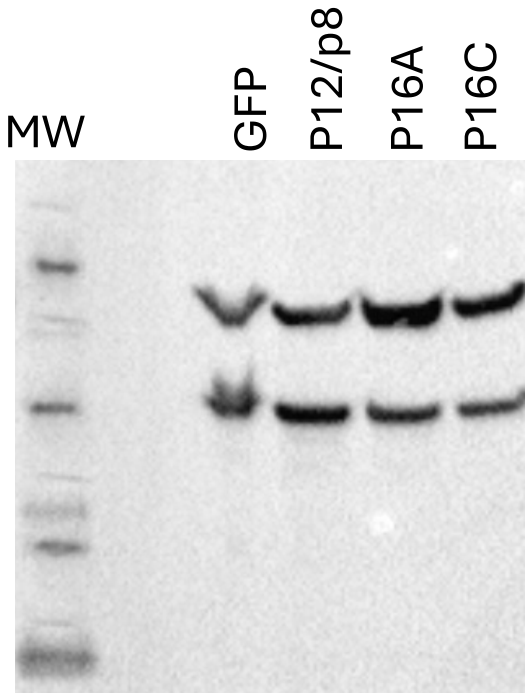

GFP (Mouse, Thermo Fisher Scientific, cat. #MA5-15256)

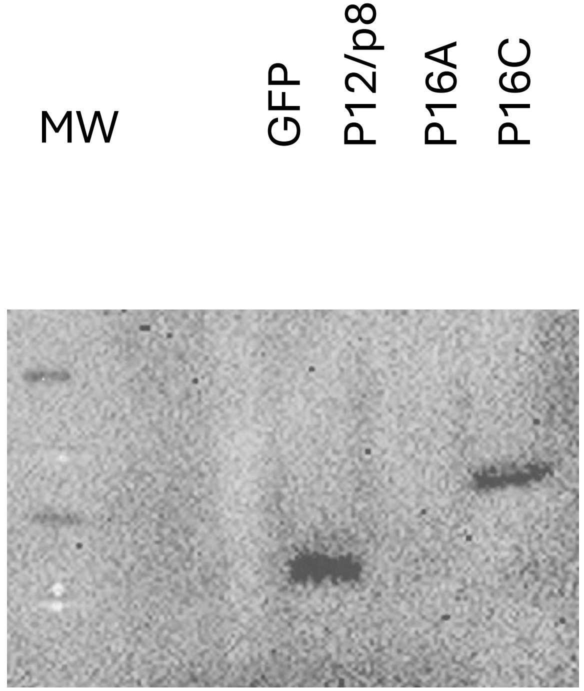

HA (Rabbit, Cell Signaling Technology, C29F4, 1:1000)

Supplementary figure 12d

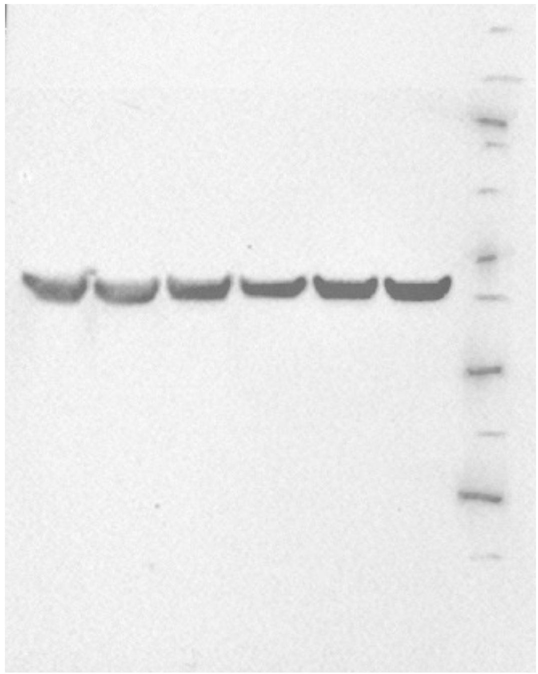

β-actin (Rabbit Cell Signaling Technology, D6A8, 1:1000)

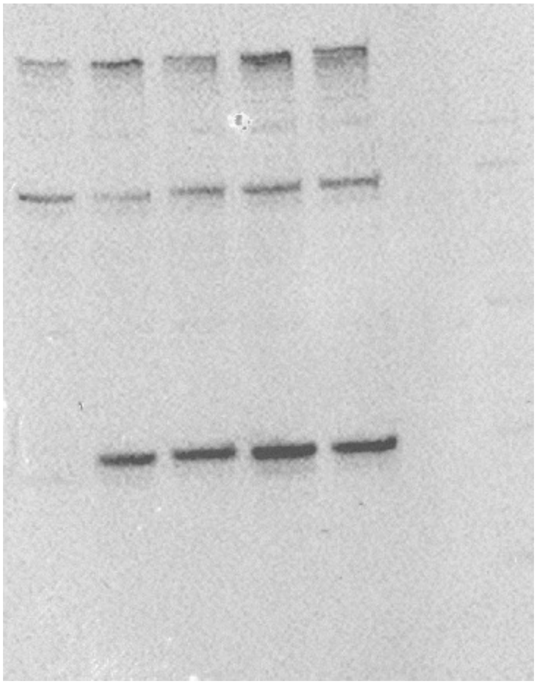

GFP (Mouse, Thermo Fisher Scientific, cat. #MA5-15256)

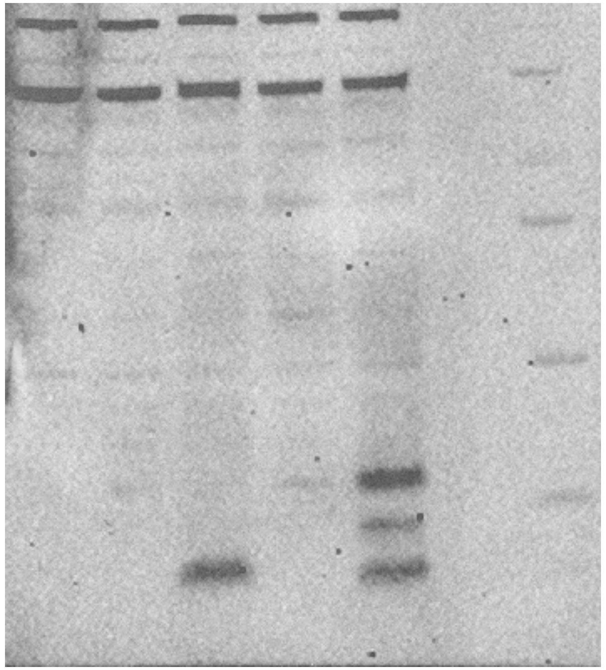

HA (Rabbit, Cell Signaling Technology, C29F4, 1:1000)

Figure 6b,c and Supplementary Table 3

Study_1_HTLV-1A_DG8Z_TMN_TiT
all_lung_lobes_sacrifice_DNA

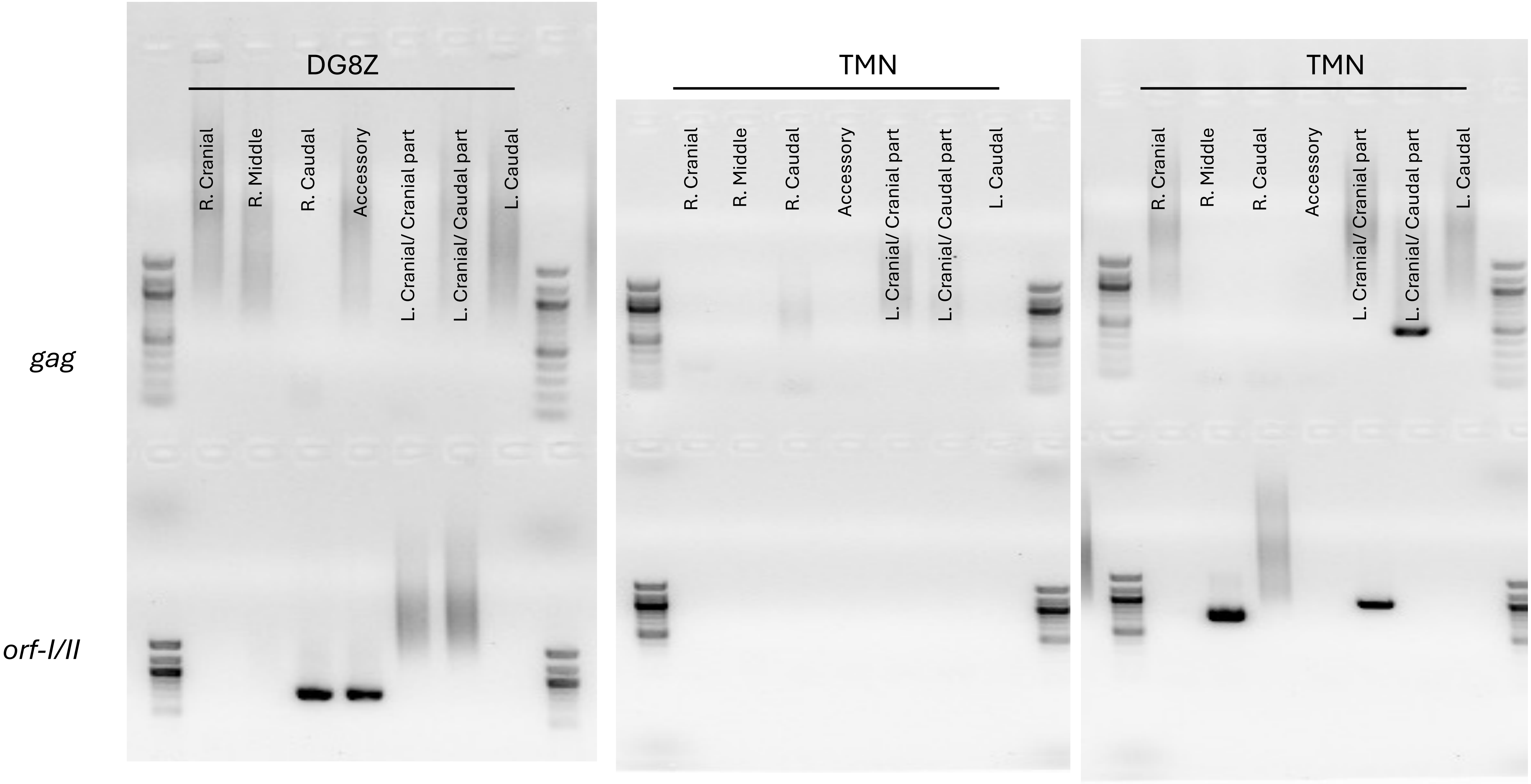

Figure 6b,c and Supplementary Table 3

Study_1 _HTLV-1A/C$_{ol\text{-}L}$_DG8Z_TMN_TiT
all_lung_lobes_sacrifice_*gag*_*rex-orf-I*_Transcript

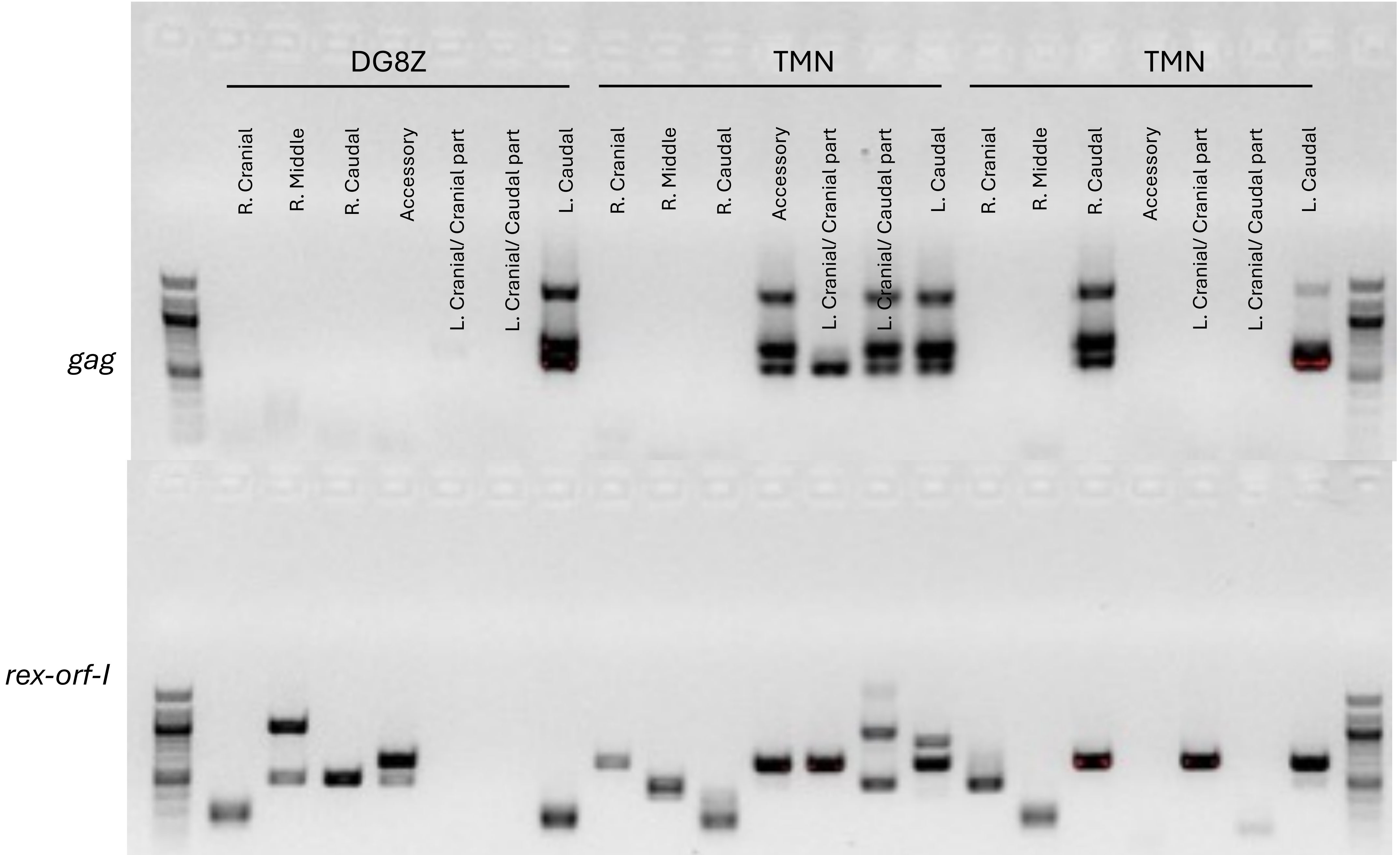

Figure 6b,c and Supplementary Table 3

Study_1 _HTLV-1A/C$_{ol\text{-}L}$ _DG8Z_TMN_TiT
all_lung_lobes_sacrifice_*gag*_*rex-orf-I*_Transcript

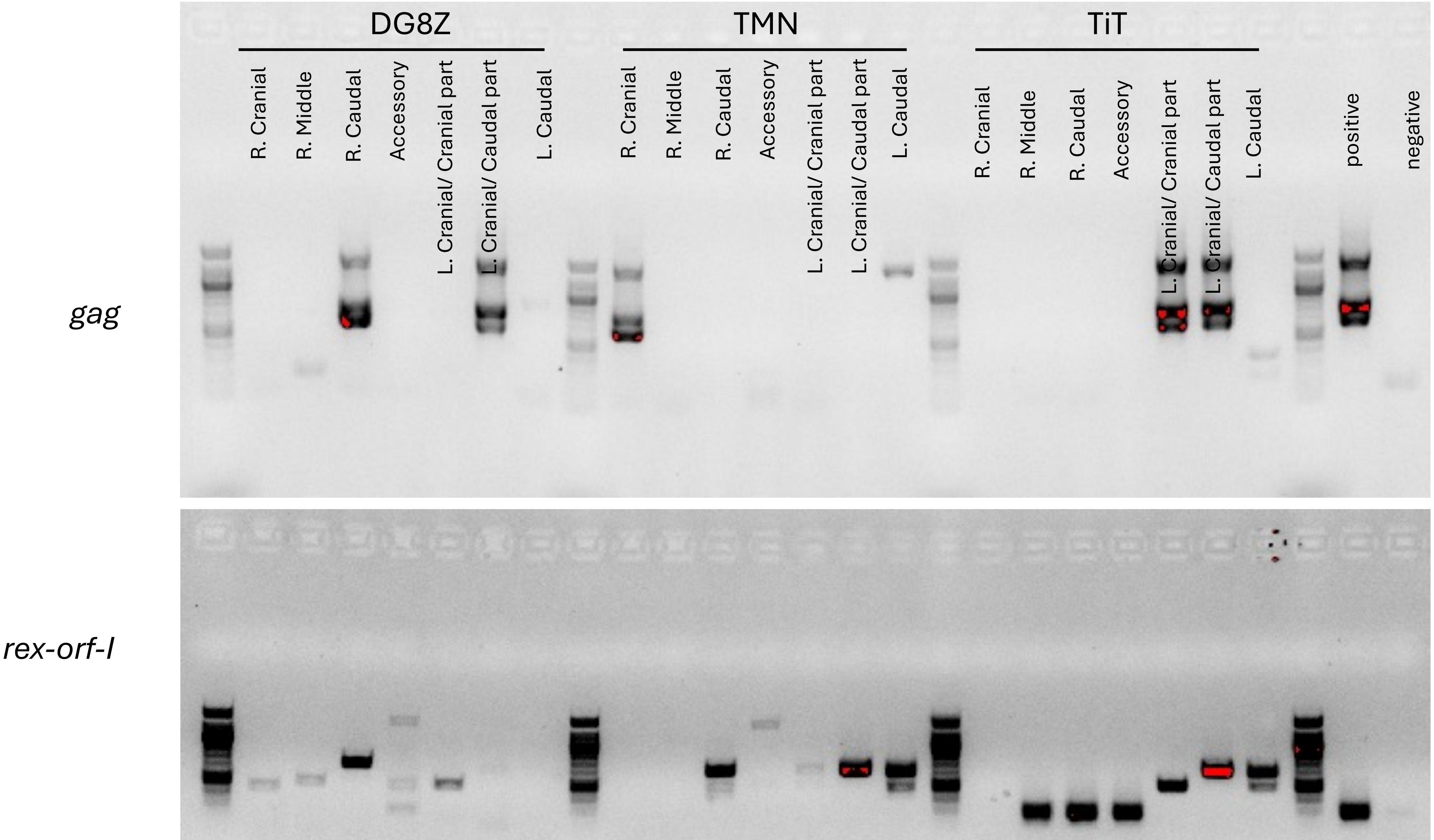

Figure 6b,c and Supplementary Table 3

Study_1 _HTLV-1A/C$_{ol\text{-}L}$ _DG8Z_TMN_TiT
all_lung_lobes_sacrifice_ *usHBZ*_Transcripts

usHBZ

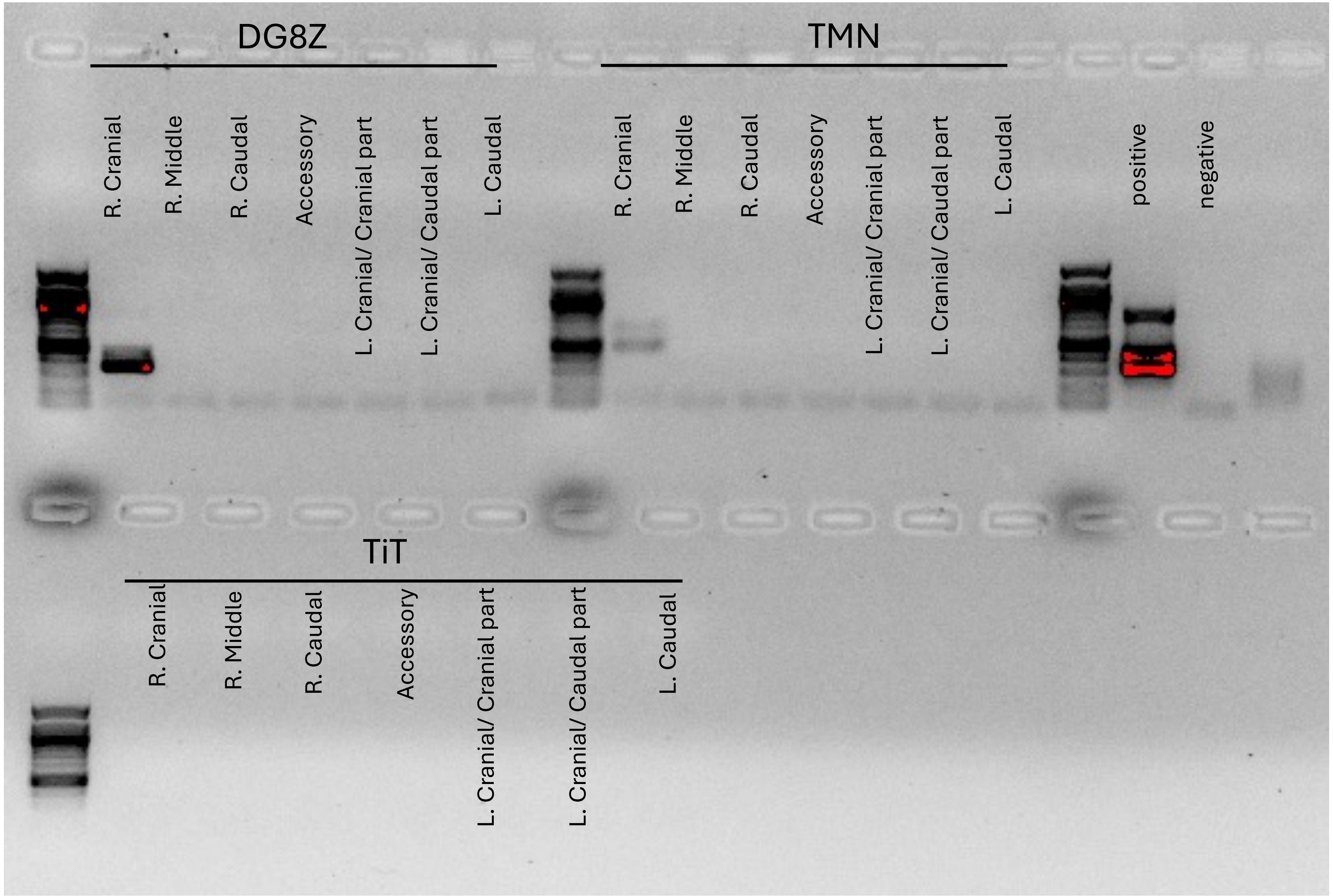

Figure 6b,c and Supplementary Table 3

Study_1 _HTLV-1A/C$_{ol\text{-}L}$ _DG8Z_TMN_TiT
all_lung_lobes_sacrifice_ *sHBZ*_Transcripts

sHBZ

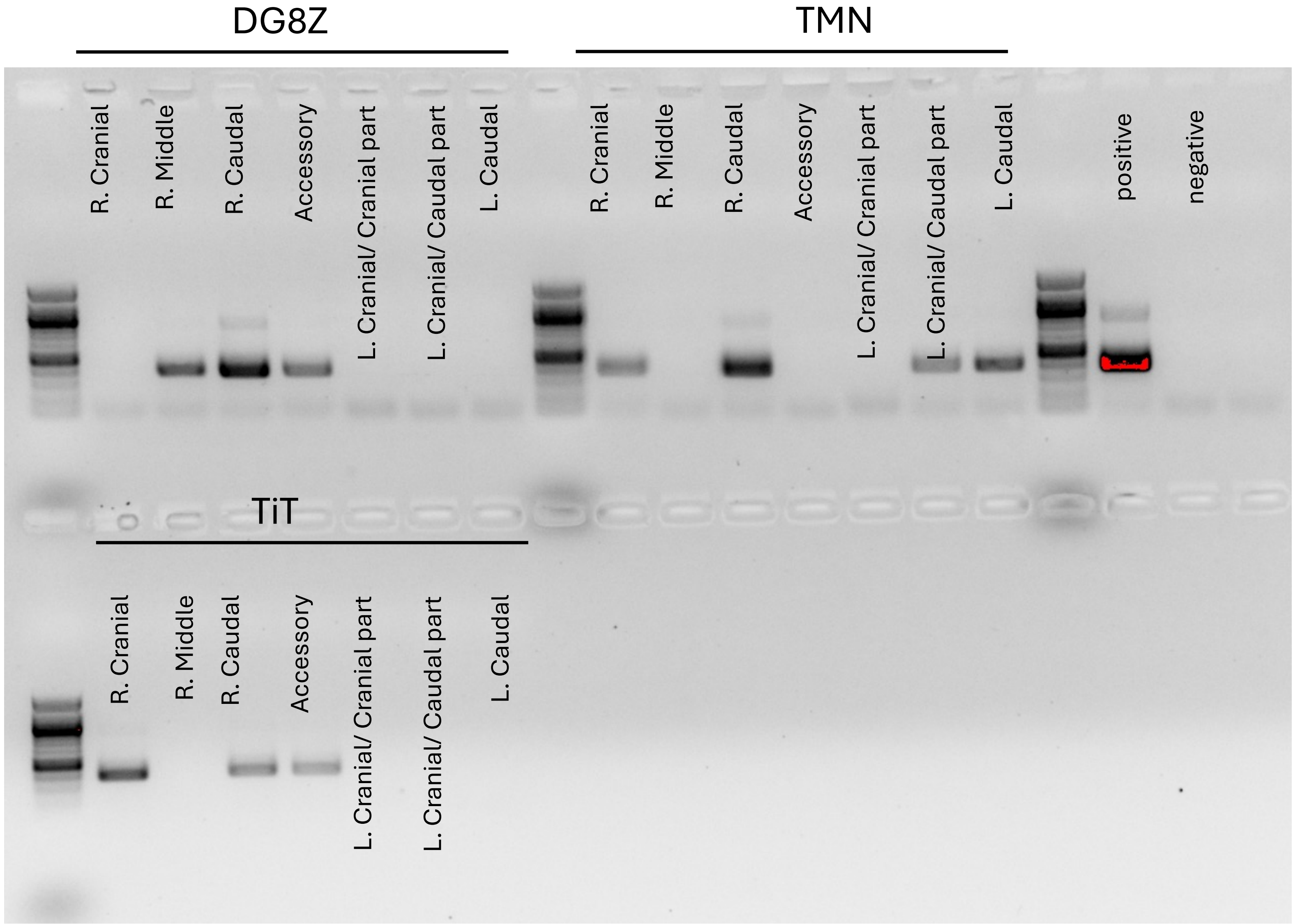

Figure 6b,c and Supplementary Table 3

Study_2_HTLV-1A_RA6_TRE
all_lung_lobes_sacrifice_DNA

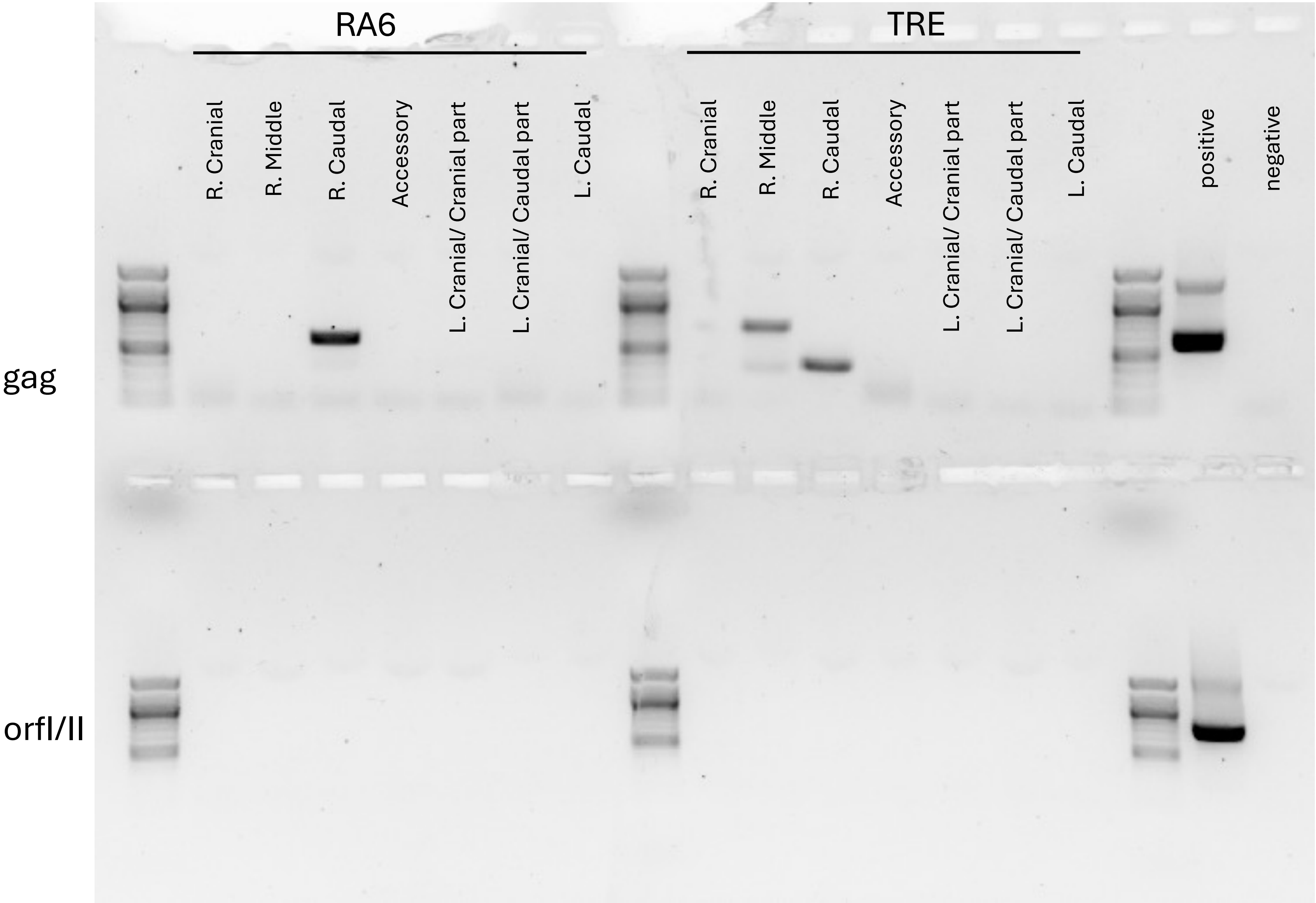

Figure 6b,c and Supplementary Table 3

Study_2_HTLV-1A_TZW_RH5
all_lung_lobes_sacrifice_DNA

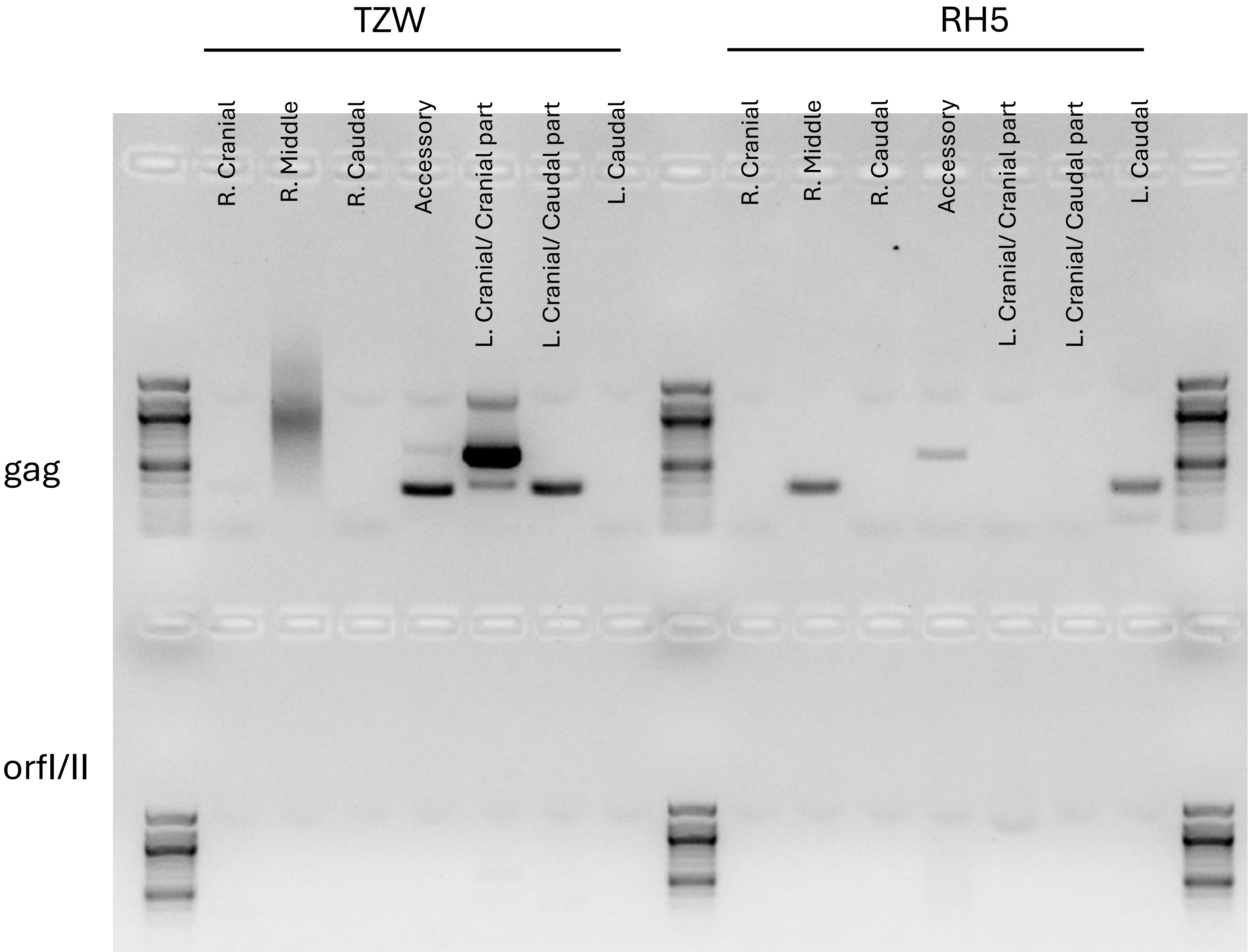

Figure 6b,c and Supplementary Table 3

Study_2 _RKF_DHF6
all_lung_lobes_sacrifice_DNA

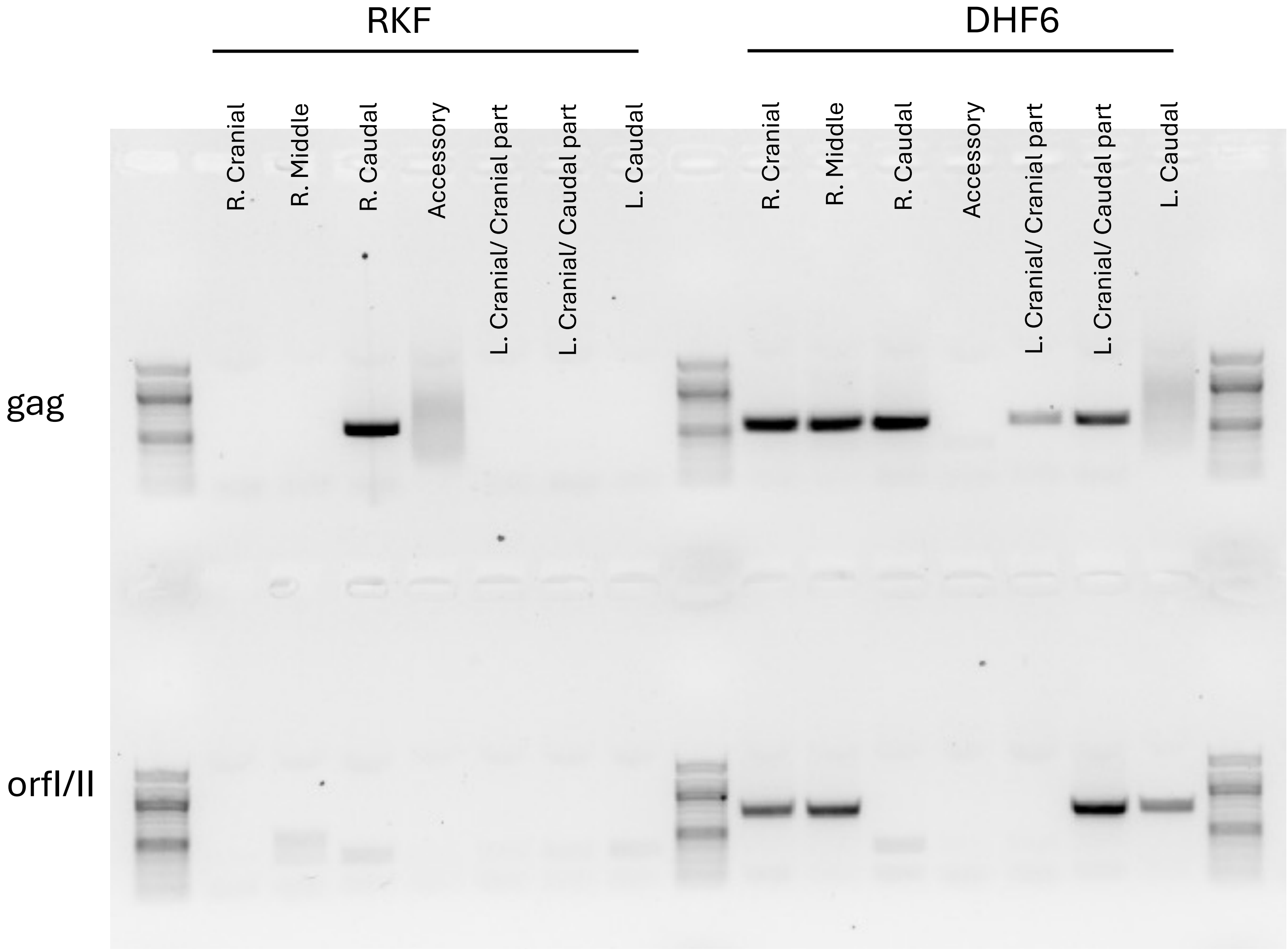

Figure 6b,c and Supplementary Table 3

Study_2_HTLV-1A_RA6_TRE
all_lung_lobes_ *gag_rex-orf-I* _sacrifice_Transcripts

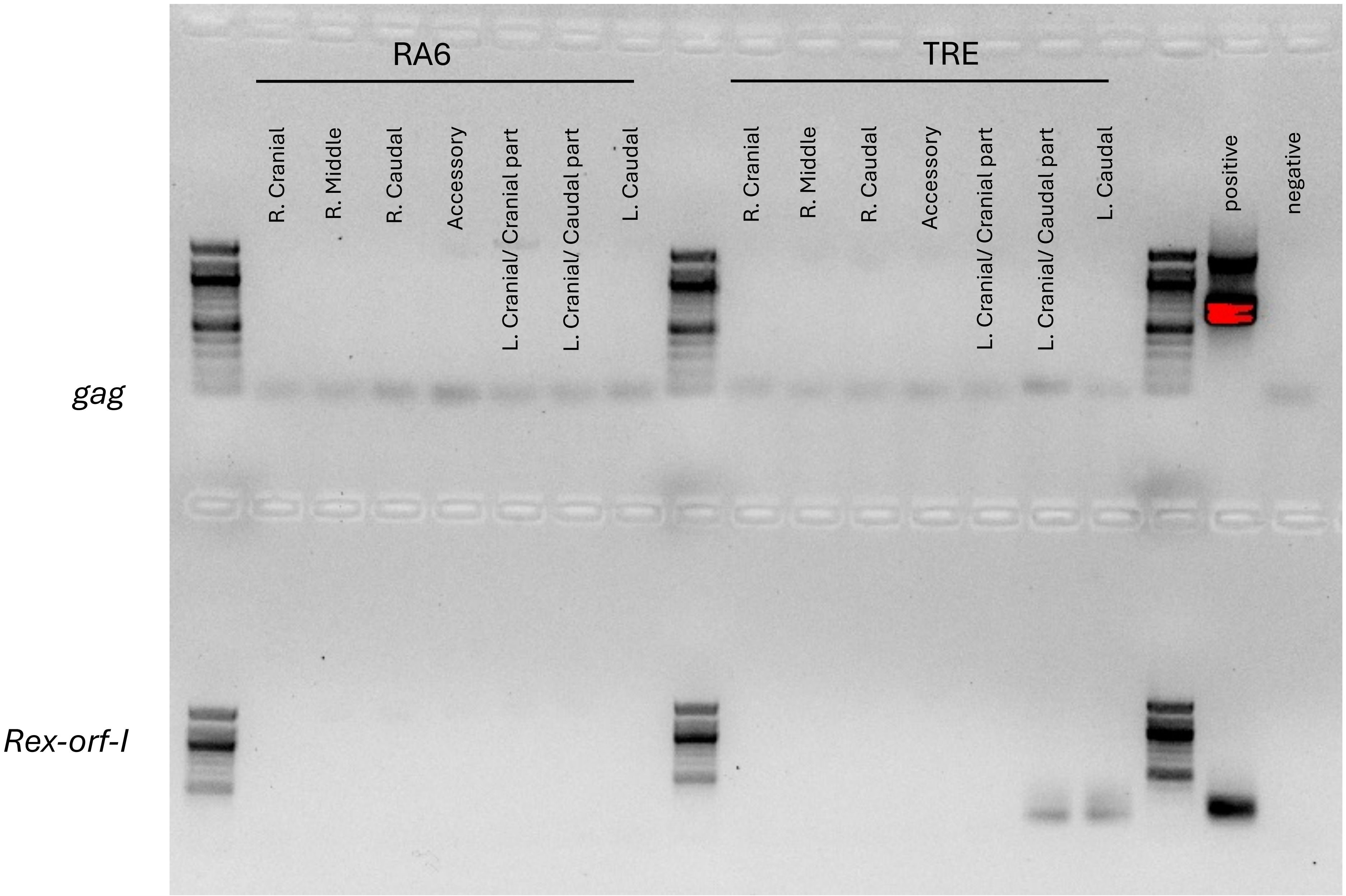

Figure 6b,c and Supplementary Table 3

Study_2_HTLV-1A_RA6_TRE
all_lung_lobes_sacrifice_*sHBZ*_*usHBZ*_Transcripts

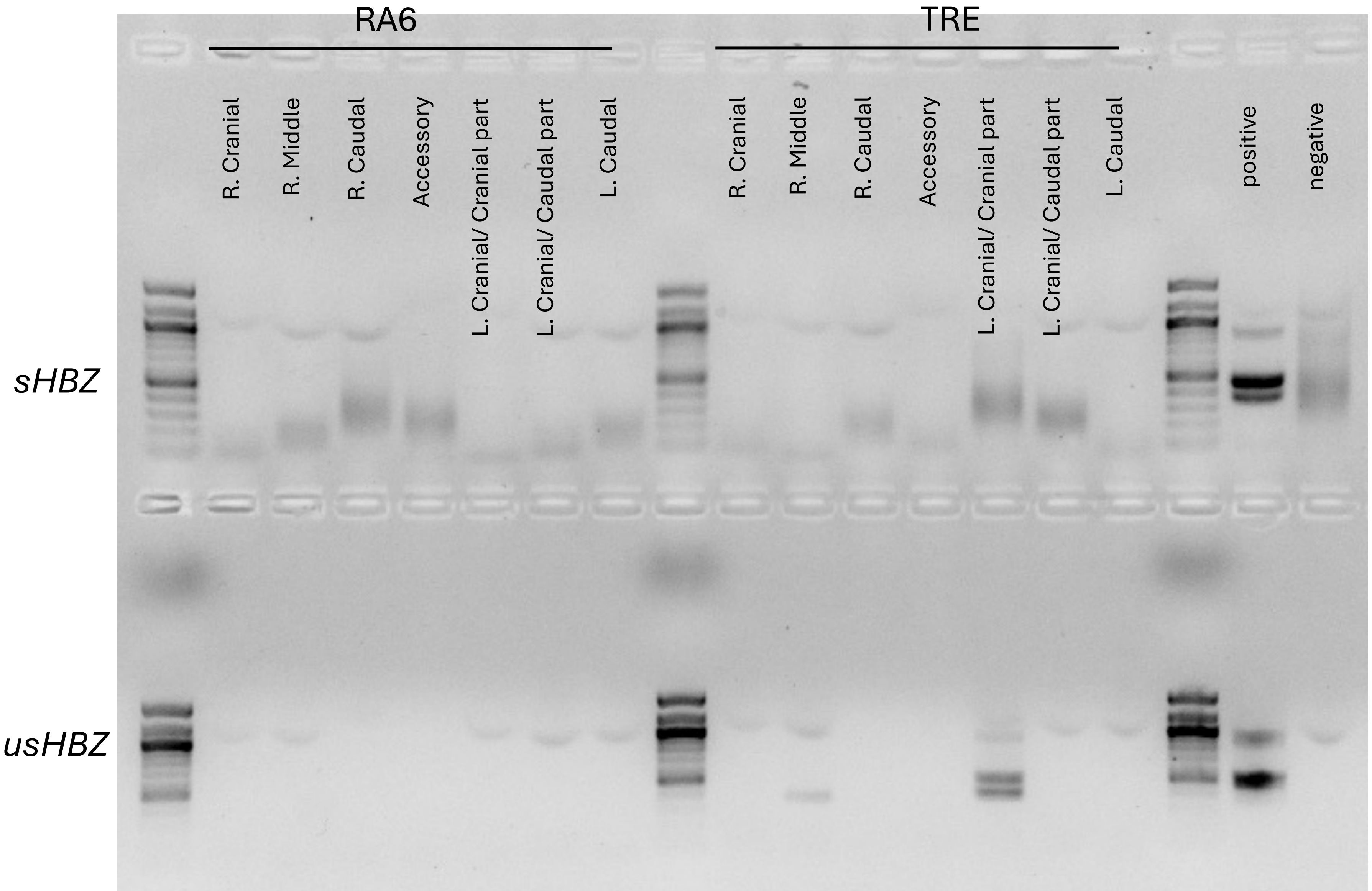

Figure 6b,c and Supplementary Table 3

Study_2_HTLV-1A_TZW_RH5
all_lung_lobes_ *gag_rex-orf-I*_sacrifice_Transcripts

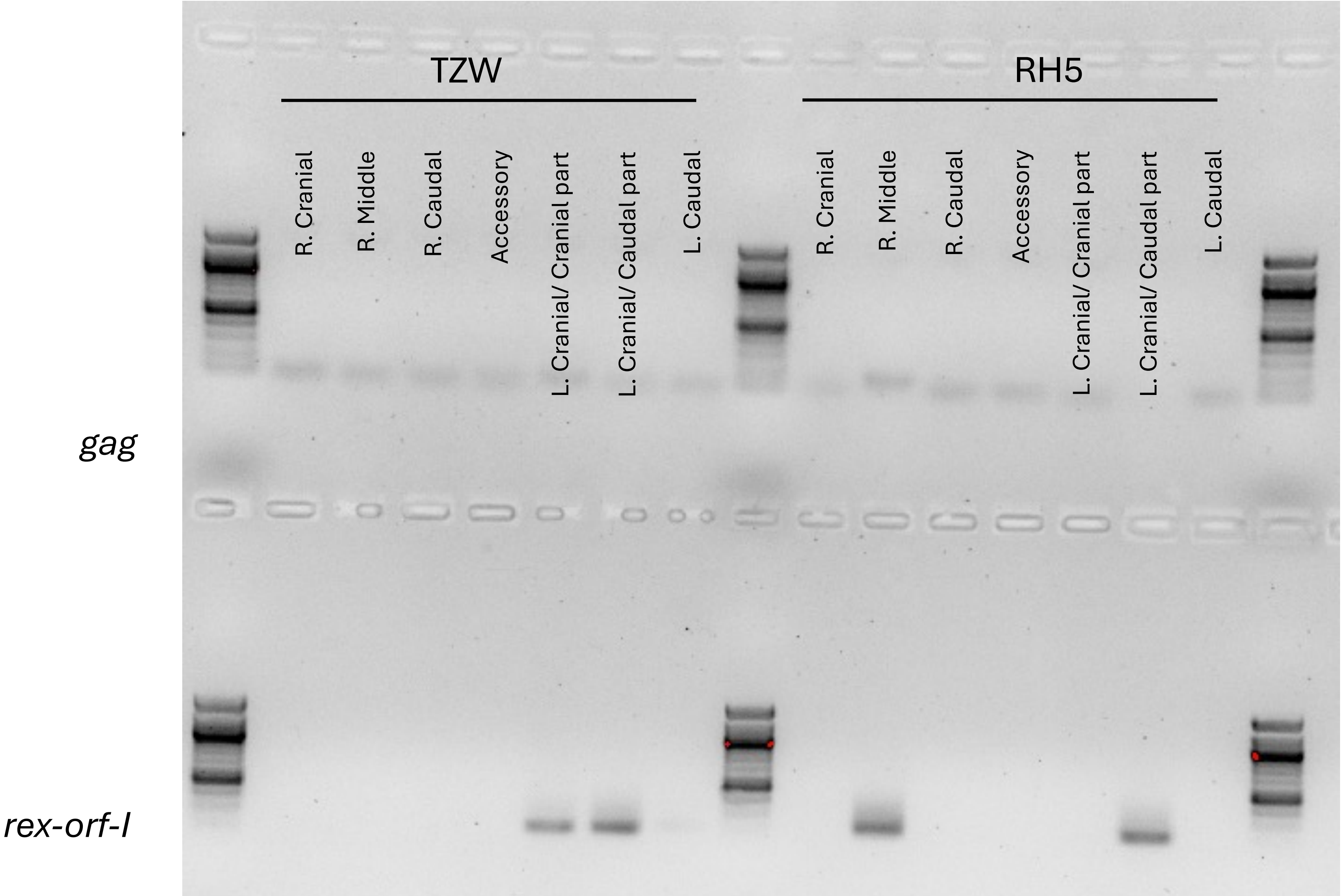

Figure 6b,c and Supplementary Table 3

Study_2_HTLV-1A_TZW_RH5
all_lung_lobes_sacrifice_*sHBZ*_*usHBZ*_Transcripts

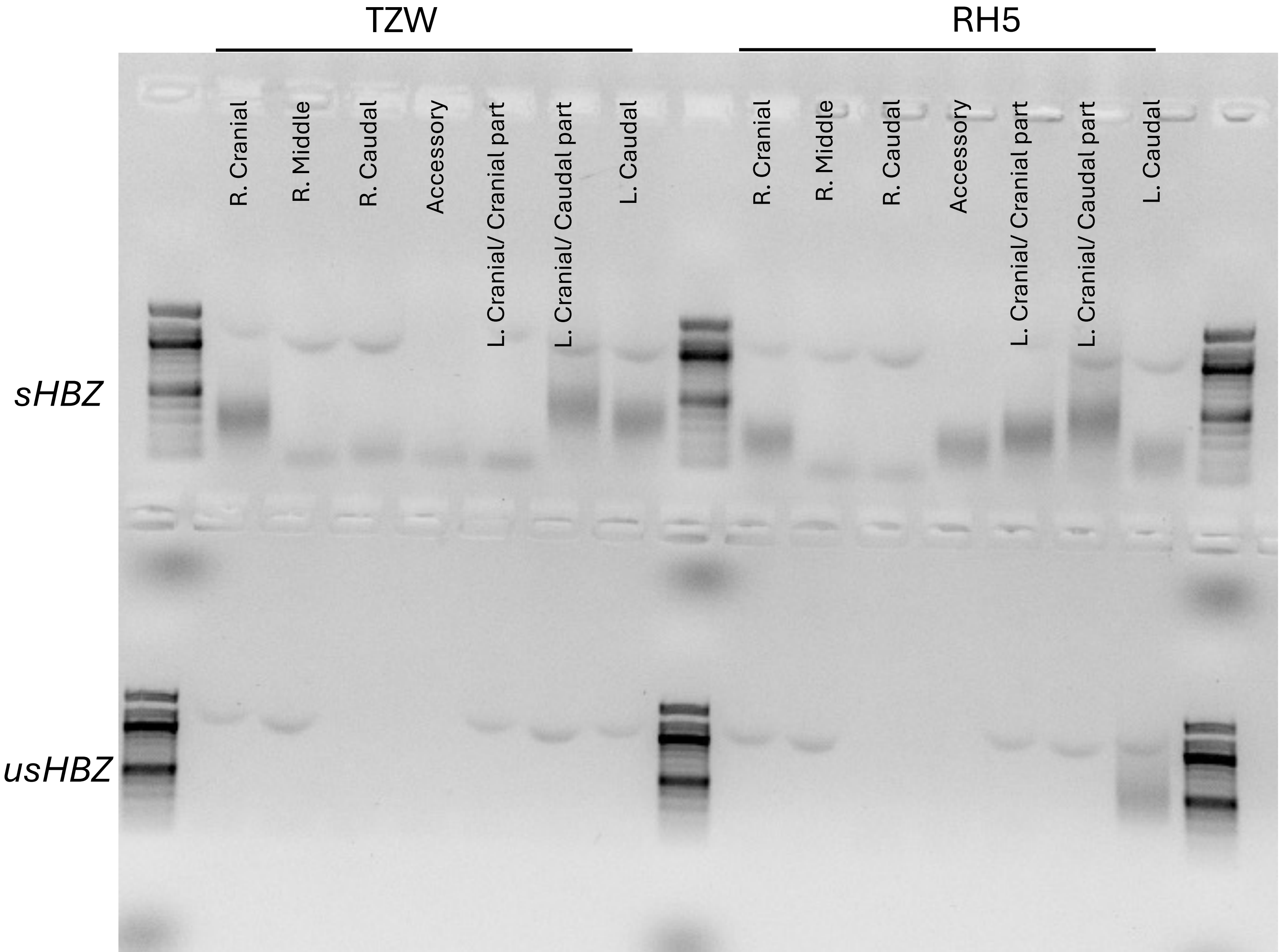

Figure 6b,c and Supplementary Table 3

Study_2_RKF_DHF6
all_lung_lobes_ *gag_rex-orf-I* _sacrifice_Transcripts

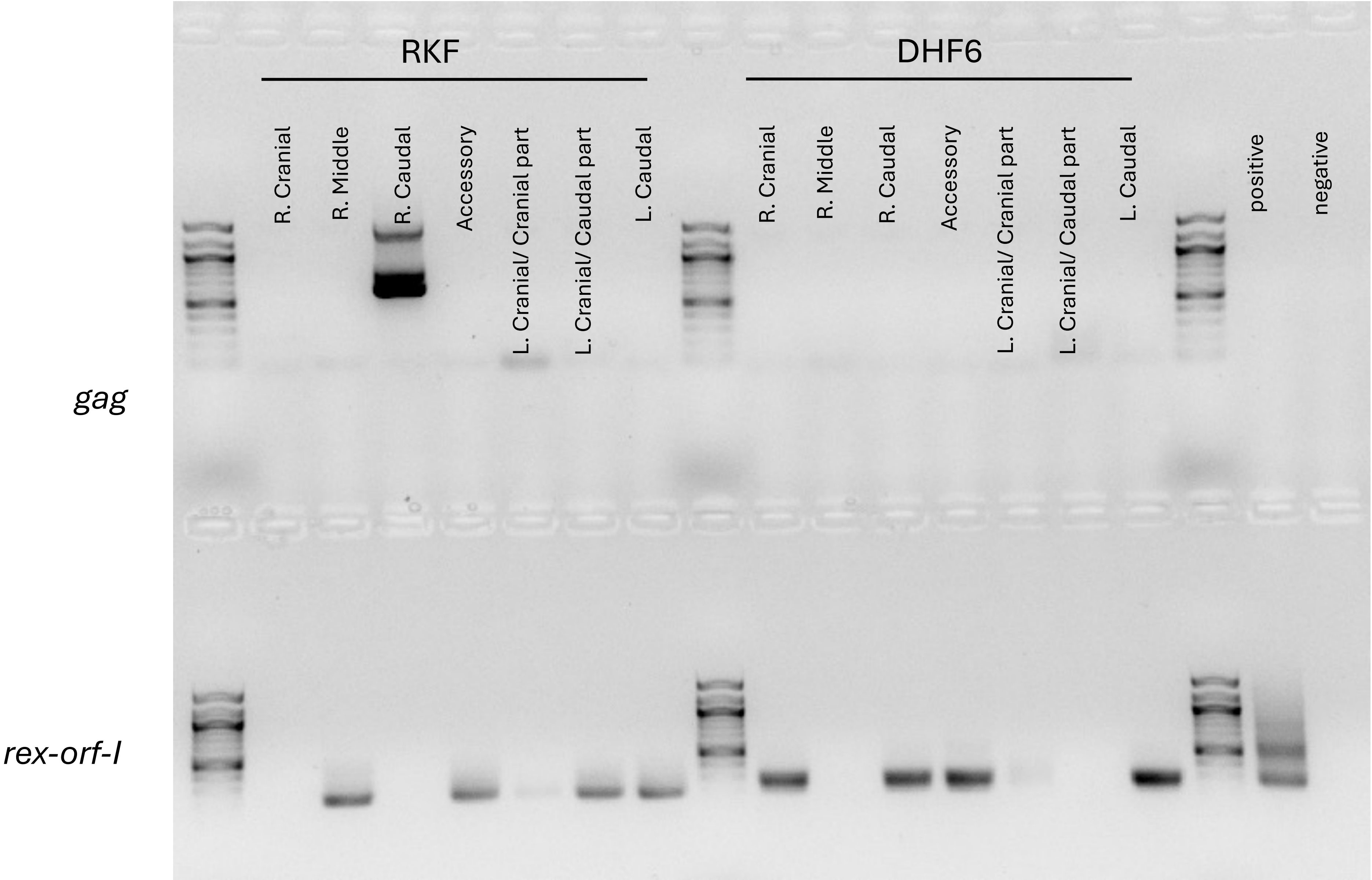

Figure 6b,c and Supplementary Table 3

Study_2_RKF_DHF6
all_lung_lobes_sacrifice_*sHBZ*_*usHBZ*_Transcripts

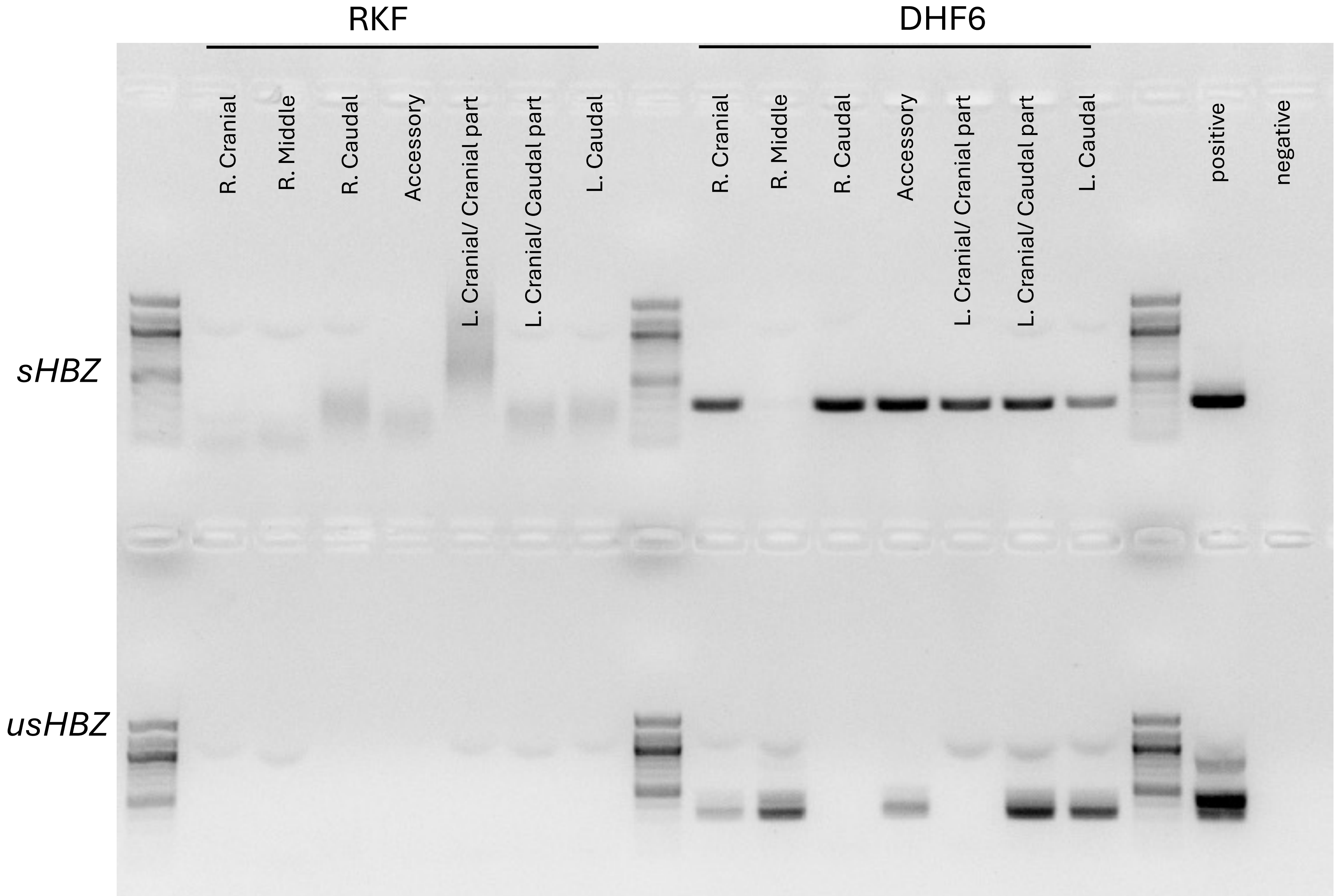

Supplement: Supplementary file 1 — Supplementary Information [file 41467_2025_63325_MOESM1_ESM.pdf]
