# Supplementary material for: High expression of Rex-orf-I and HBZ mRNAs and bronchiectasis in lung of HTLV-1A/C infected macaques
Source: Nat Commun. 2025 Sep 26;16:8470. doi: 10.1038/s41467-025-63325-1 (PMC12474910; doi:10.1038/s41467-025-63325-1)
Supplement: Supplementary file 3 — Reporting Summary [file 41467_2025_63325_MOESM3_ESM.pdf]

nature portfolio

Corresponding author(s): Genoveffa Franchini

Last updated by author(s): Aug 7, 2025

Reporting Summary

Nature Portfolio wishes to improve the reproducibility of the work that we publish. This form provides structure for consistency and transparency in reporting. For further information on Nature Portfolio policies, see our Editorial Policies and the Editorial Policy Checklist.

Statistics

For all statistical analyses, confirm that the following items are present in the figure legend, table legend, main text, or Methods section.

n/a	Confirmed	
☐	☒	The exact sample size (*n*) for each experimental group/condition, given as a discrete number and unit of measurement
☐	☒	A statement on whether measurements were taken from distinct samples or whether the same sample was measured repeatedly
☐	☒	The statistical test(s) used AND whether they are one- or two-sided<br>*Only common tests should be described solely by name; describe more complex techniques in the Methods section.*
☐	☒	A description of all covariates tested
☐	☒	A description of any assumptions or corrections, such as tests of normality and adjustment for multiple comparisons
☐	☒	A full description of the statistical parameters including central tendency (e.g. means) or other basic estimates (e.g. regression coefficient) AND variation (e.g. standard deviation) or associated estimates of uncertainty (e.g. confidence intervals)
☒	☐	For null hypothesis testing, the test statistic (e.g. *F*, *t*, *r*) with confidence intervals, effect sizes, degrees of freedom and *P* value noted<br>*Give P values as exact values whenever suitable.*
☒	☐	For Bayesian analysis, information on the choice of priors and Markov chain Monte Carlo settings
☐	☒	For hierarchical and complex designs, identification of the appropriate level for tests and full reporting of outcomes
☒	☐	Estimates of effect sizes (e.g. Cohen's *d*, Pearson's *r*), indicating how they were calculated

Our web collection on statistics for biologists contains articles on many of the points above.

Software and code

Policy information about availability of computer code

Data collection: n/a

Data analysis:
- Prism 10 for macOS, Version 10.2.3 (347), June 24, 2024
- FlowJo 10.10.0 (TreeStar, Inc.)
- FACSymphony A5
- FACSDiva software (BD Biosciences)
- R package edgeR version 3.30.3
- Microsoft Excel for Mac, Version 16.86 (24060916)
- SnapGene version 6.2 (GSL Biotech LLC)
- Image Lab Version 6.0.1 build 34 (Bio-Rad Laboratories, Inc.)
- Clustal Omega Multiple Sequence Alignment (MSA). (European Molecular Biology Laboratory-EBI) open source
- MACS2 (version 2.2.7.1)
- Olink NPX Signature software Version 1.12 (February 16, 2024)
- PhenoImager® HT 2.0 (AKOYA, Biosciences®).
- pheatmap R package 1.1.30

For manuscripts utilizing custom algorithms or software that are central to the research but not yet described in published literature, software must be made available to editors and reviewers. We strongly encourage code deposition in a community repository (e.g. GitHub). See the Nature Portfolio guidelines for submitting code & software for further information.

Data

Policy information about availability of data

All manuscripts must include a data availability statement. This statement should provide the following information, where applicable:

- Accession codes, unique identifiers, or web links for publicly available datasets
- A description of any restrictions on data availability
- For clinical datasets or third party data, please ensure that the statement adheres to our policy

The sequence of the pAB_HTLV-1A/CoI-L chimeric molecular clone have been deposited in NCBI GenBank nucleotide database under the accession no. PP860917. The HTLV-1C viral nucleotide sequence used in the cloning derived from virus isolated from PBMCs of an infected donor obtained from the Laboratory of Dr. Damian FJ Purcell (accession nos. PP596271, PP596272, PP596273, PP596274, PP596275, PP596276, PP596277, PP596278, PP596279, PP596280, PP596281, PP596282, PP596283, PP596284, PP596285, PP596286, PP596287, PP596288, PP596289, PP596290, PP596291, PP596292 for all patient proviruses). The data generated in this study has been deposited in Zenodo under accession code 10.5281/zenodo.16755081 https://zenodo.org/uploads/1675508166. Source data file are provided with this paper.

Research involving human participants, their data, or biological material

Policy information about studies with human participants or human data. See also policy information about sex, gender (identity/presentation), and sexual orientation and race, ethnicity and racism.

Reporting on sex and gender	Research was not conducted in human participants. This work does not generate human data. Clinical data from a separate study are used in analysis. All information relevant to that study is described in: Hirons, A.Y., D; Jansz, N; Ellenberg, P; Franchini, G; Einsiedel, L; Khoury, G; Purcell DFJ. Unique viral genomic features of HTLV-1 subtype-C in Central Australia and pathogenetic implications. Retrovirology (2024).
Reporting on race, ethnicity, or other socially relevant groupings	n/a
Population characteristics	n/a
Recruitment	n/a
Ethics oversight	n/a

Note that full information on the approval of the study protocol must also be provided in the manuscript.

Field-specific reporting

Please select the one below that is the best fit for your research. If you are not sure, read the appropriate sections before making your selection.

☒ Life sciences ☐ Behavioural & social sciences ☐ Ecological, evolutionary & environmental sciences

For a reference copy of the document with all sections, see nature.com/documents/nr-reporting-summary-flat.pdf

Life sciences study design

All studies must disclose on these points even when the disclosure is negative.

Sample size	The samples size of each group was determined by consultation with our statistician. Based on our previous study following different type of depletions the groups exposed to the HTLV-1A virus showed a range of infection between 30% and 100% with an average of 76% of animals infected per group. on another hand groups exposed to another virus (HTLV-1p12KO virus) carrying a single point mutation of the translation initiation codon of a regulatory gene (orf-I), showed an infection rate between 20 and 100% with an average of 48.3% of animals infected per group. Considering that in the previous studies the use of up to 5 animals per group, allowed us to detect a difference in the frequency of the infection and induction of immune-responses, and since that we expect that the chimeric virus to behave similarly to HTLV-1p12KO virus, the use of up to 5 animals per group was enough to identify differences between the groups exposed to different type of viruses. The primary outcome of the study is infectivity, which means the presence or absence of established infection after viral exposure. With only 5 animals, a binary outcome of this sort does not provide a lot of power, but based on our p12KO virus studies we expect a clear difference between HTLV-1 A/CoI-L in immune competent animals compared to depleted animals (Moles et al, PLos. Pathogens 2022). The 95% confidence intervals(CI) for observed frequencies of a binary outcome in a group of size 5. If we have a probability that lies outside several of the CIs, then we can state that those outcomes, if observed, will be evidence rejecting the null hypothesis that this group has the given probability. For example, if our prior probability of infectivity is 0.1, then any outcome of 4 or more out of 8 will reject the null hypothesis with 95%confidence. This does not give us an accurate calculation of how likely 4+ out of 8 is, but we can as our previous studies (Valeri et al, 2010; Pise-Masison et al, 2014; Moles et al, 2022) to assess possible outcomes.In comparing individual groups, the Fisher's exact test would be most appropriate. If one group has a In the in vitro studies at least three biological replicates were run for each experiment in order to achieve significance. Except the whole cord blood cell experiment where just two independent donors were used. However in vivo study run in macaques considering NIH statistician consultation, we determined that for the pilot study up to 8 animals per group will allow us to assess the proposed questions of infectivity. Moreover, in our previous studies, following different types of depletions, groups exposed to the WT virus showed a range of infection between 30% and 100% with an average of 76% per group. On another hand, groups exposed to the p12KO

virus, showed an infection range between 20 and 100% with an average of 48.3% of animals infected per group. Considering that in the previous studies the use of up to 5 animals per group, allowed us to detect a difference in the frequency of infection and induction of immune-responses, and that we expect the chimeric virus to behave similarly to p12KO virus; the use of up to 8 animals per group increases the chance to identify differences between the groups exposed to different type of viruses. "Based on the analysis, 5 animals per group will provide sufficient statistical power to detect a difference in infectivity."

The primary outcome of the study is infectivity, which means the presence or absence of established infection after viral exposure. With only 5 animals, a binary outcome of this sort does not provide a lot of power, but based on our p12KO virus studies we expect a clear difference between HTLV-1 A/CoI-L in immune competent animals compared to depleted animals (Moles et al, PLos. Pathogens 2022). The 95% confidence intervals(CI) for observed frequencies of a binary outcome in a group of size 5. If we have a probability that lies outside several of the CIs, then we can state that those outcomes, if observed, will be evidence rejecting the null hypothesis that this group has the given probability. For example, if our prior probability

of infectivity is 0.1, then any outcome of 4 or more out of 8 will reject the null hypothesis with 95%confidence. This does not give us an accurate calculation of how likely 4+ out of 8 is, but we can as our previous studies (Valeri et al, 2010; Pise-Masison et al, 2014; Moles et al, 2022) to assess possible outcomes.In comparing individual groups, the Fisher's exact test would be most appropriate. If one group has a probability of 0.10 and the other has a probability of 0.80, then there is 79% power of this test having a result of p< 0.05. If the second group has probability 0.85 or 0.90, the power increases to 87% or 93%. If infectivity is rarer, specifically, 0.05, then probabilities in the second group of 0.75, 0.80 or 0.85 will have power of 80%, 88% or 93%.

Data exclusions

No data were excluded.

Replication

The nature of the samples analyzed in the present studies, the limited amount of each sample collected from each animal and the cost of the non-human primate studies do not allow us to replicate the experiments. In the reported assays the replicates are represented by each animal enrolled in the study. All the data have been obtained with validated assays that have been used in previous publish work.

In the in vitro studies at least three biological replicates were run for each experiment in order to achieve significance. Except the whole cord blood cell experiment where just two independent donors were used.

Randomization

Nineteen male and female rhesus macaques uninfected with SIV/SHIV as demonstrated by several consecutive negative PCRs and seronegative for simian T-cell lymphotropic virus 1 at the initiation of the study were randomized into three groups based on sex, age, weight, and their prior enrollment in other studies (Supplementary Table 1)39. Five animals from the α-CD8/NK/Clodrosome®/HTLV-1A/CoI-L group and ten animals from α-CD8/NK/Clodrosome®/HTLV-1A were treated for three consecutive days (Day -3, -2, -1) with an anti-CD8 monoclonal antibody the clone MT807R1 targeting the α/α chain of the CD8+ lymphocytes and NK cells, in addition to a single dose of Clodrosome® delivered in Liposome (Day -1) targeting the phagocytic cells. Four control animals in the IgG/Liposome/HTLV-1A/CoI-L group were treated for three consecutive days (Day -3, -2, -1) with the isotype control antibody IgG OKT3, reactive against the human CD3 molecules, in addition to a single dose (Day -1) of Encapsome® corresponding to an empty Liposome. Both antibodies, anti-CD8 and IgG OKT3, were purchased from the NHP Reagent Resource Program (University of Massachusetts Medical School, Worcester, MA) while Clodrosome® (cat. #CLD-8909) and Encapsome® (cat. #CLD-8910) were purchased from Encapsula NanoSciences (Brentwood, TN). All treatments were administrated intravenously at 5 mg/kg/dose/day prior to the intravenous inoculation of 1108 or 1.5108 lethally-irradiated 729.6 lymphoblastoid B-cell lines producing either HTLV-1A or HTLV-1A/CoI-L, respectively. The inoculated cell number was normalized for p19 Gag antigen production and viral DNA level to reflect the amount used in our previous studies33,35,39. Animals were monitored for over 21 weeks post viral inoculation and then euthanized to study viral dissemination and pathogenesis in tissues except for TMN, DG8Z, TiT, RA6, TRE, TZW, RH5, RKF and DHF6 that were monitored for over 48 weeks. Except for randomization purposes, the sex of the animals was not considered as descriminating factor in our study. Since the aim of our study was to investigate the infectivity and the pathogenicity of the newly constructed chimeric molecular clone, both male and female were used in each group.

Blinding

For macaques studies (antibody/treatment administration and virus inoculation): the investigators that prepared the reagents were unblinded whereas the veterinarian staff was blinded. For immunological assays and virological assays: during the execution and analyses of the experiments the investigators were blinded. Investigators were unblinded after generating the results in order to perform the final analyses of the data.

Reporting for specific materials, systems and methods

We require information from authors about some types of materials, experimental systems and methods used in many studies. Here, indicate whether each material, system or method listed is relevant to your study. If you are not sure if a list item applies to your research, read the appropriate section before selecting a response.

Materials & experimental systems

n/a	Involved in the study	
	x	Antibodies
	x	Eukaryotic cell lines
x		Palaeontology and archaeology
	x	Animals and other organisms
x		Clinical data
x		Dual use research of concern
x		Plants

Methods

n/a	Involved in the study	
x		ChIP-seq
	x	Flow cytometry
x		MRI-based neuroimaging

Antibodies

Antibodies used

As reported in the Method section:
1- For the primary CD4+ cells coculture experiments PE anti-CD4 (clone L200; cat. # 550630, BD Biosciences) was used for cell isolation.
2- For the whole blood cell phenotyping, the Following antibodies were used: FITC anti-CD8 (clone DK25; cat. #FCMAB176F; EMB Millipore Corp.), BB700 anti-CD14 (clone M5E2; cat. #745790; BD Biosciences), PE-Cy5 anti-CD95 (clone DX2; cat. #305610; BioLegend), PE-Cy7 anti CD159 (NKG2a) (clone Z199; cat. #B10246; Beckman Coulter), APC anti-CD66abce (clone TET2; cat. #130-118-539; Miltenyi Biotec), Alexa 700 anti-CD3 (clone SP34-2; cat. #557917; BD Biosciences), APC-Cy7 anti-CD11b (clone ICRF44; cat. #557754; BD Biosciences), BV421 anti-CD16 (clone 3G8; cat. #562874; BD Biosciences), BV570 anti-CD20 (clone 2H7; cat. #302332; BioLegend), BV750 anti-CD4 (clone L200; cat. #747202; BD Biosciences), BV786 anti-CD45 (clone D058-1283; cat. #563861; BD Biosciences), BUV496 anti-CD28 (clone CD28.2; cat. #741168; BD Biosciences), BUV563 anti-CD49d (clone 9F10; cat. #749455; BD Biosciences), BUV661 anti-HLA-DR (clone G-46-6; cat. #612980; BD Biosciences), BV711 anti-CD11c (clone B-ly6; cat. #741139; BD Biosciences), BV650 anti-CD123 (clone 7G3; cat. #572392; BD Biosciences), BUV805 anti-CD8 (clone SK1; cat. #612889; BD Biosciences). Blue LIVE/Dead viability dye (cat. #L23105; Thermo Fisher Scientific, Waltham, MA) was used to exclude dead cells.
3- To measure neutrophils, monocytes, myeloid dendritic cells (mDC) and plasmacytoid dendritic cells (pDC) in bronchoalveolar lavage (BAL) and whole blood of the animals, the following antibodies were used for cell surface staining: FITC anti-CD66abce (clone TET2; cat. #130-116-522; Miltenyi Biotec), BB700 anti-CD162 (clone KPL-1; cat. #745768; BD Biosciences), Alexa 700 anti-CD3 (clone SP34-2; cat. #557917; BD Biosciences), Alexa 700 anti-CD20 (clone 2H7; cat. #560631; BD Biosciences), APCCy7 anti-CD11b (clone ICRF44; cat. #47-0118-42; Invitrogen™), BV480 anti-CD11c (clone 3.9; cat. #748269; BD Biosciences), BV650 anti-CD8 (clone RPA-T8; cat. #563821; BD Biosciences), BV750 anti-CD206 (clone 19.2; cat. #746891; BD Biosciences), BV786 anti-CD45 (clone D058-1283; cat. #563861; BD Biosciences), BUV395 anti-123 (clone 7G3; cat. #564195; BD Biosciences), BUV496 anti-CD16 (clone 3G8; cat. #612944; BD Biosciences), BUV563 anti-CD163 (clone GH1/61; cat. #741402; BD Biosciences), BUV661 anti-HLA-DR (clone G46-6; cat. #612980; BD Biosciences), BUV737 anti-CD64 (clone 10.1; cat. #564426; BD Biosciences), BUV805 anti-CD14 (clone M5E2; cat. #612902; BD Biosciences). The following antibodies were used for intracellular staining, PE anti-MPO (clone MPO455-8E6; cat. #12-1299-42; Invitrogen™), BV421 anti-IL-8 (clone G265-8; cat. #563310; BD Biosciences), BV605 anti-TNF-(clone mAB11; cat. #502936; BioLegend) and BV711 anti-IL-10 (clone JES3-9D7; cat. #564050; BD Biosciences). CD14+ monocytes used in the efferocytosis assay were isolated using human CD14 MicroBeads (cat. #130-050-201, Miltenyi Biotec).
4- For the western blot and the immunofluorescence assays the following antibodies were used: HTLV-1 p24 Gag (cat. #4310 Applied Biological Laboratories), HTLV-1 gp46 envelope (cat. # CBMAB-V208-1154-FY, Creative Biolabs), Tax (Tab172), Tax (LT-4, cat. #MABF3063, Millipore Sigma), Tax (1A3, cat. #ab26997, Abcam), GFP (cat. #MA5-15256, Thermo Fisher Scientific,), -actin (cat. # D6A8, Cell Signaling Technology), GAPDH (cat. # D3U4C, Cell Signaling Technology), HA (cat. # C29F4, Cell Signaling Technology), anti- Mouse or anti-Rabbit secondary antibody (cat. # NA931 and NA934 respectively ThermoFisher scientific).
5- For the immunohistochemistry assay the following antibodies were used: CD3 (cat. # MCA1477, BioRad) with secondary antibody Rabbit anti-Rat IgG (Vector Laboratories), CD20 (cat. # M0755, DAKO/Agilent), Microglia/Iba1 (cat. # CP290, Biocare), Smooth Muscle Actin (cat. # ab5694, Abcam)

Validation

- All the antibodies used are reported as cross-reactive with the Monkey either on Reactivity database of the Nonhuman Primate Reagent Resource (NHPRR) website (https://www.nhpreagents.org/) or by the manufacturers' websites.
- CD8 (DK25) : https://www.nhpreagents.org/ReactivityDatabase
- CD14 (M5E2): https://www.nhpreagents.org/ReactivityDatabase
- CD95 (DX2): https://www.biolegend.com/fr-ch/products/pe-cyanine5-anti-human-cd95-fas-antibody-644
- CD159 (clone Z199): https://www.nhpreagents.org/ReactivityDatabase
- CD66abce (TET2): https://www.miltenyibiotec.com/US-en/products/cd66abce-antibody-anti-humantet2.html#conjugate=fitc:size=100-tests-in-200-ul
- CD3 (SP34-2): https://www.bdbiosciences.com/en-eu/products/reagents/flow-cytometry-reagents/research-reagents/single-colorantibodies-ruo/alexa-fluor-700-mouse-anti-human-cd3.557917?tab=product_details
- CD11b (ICRF44): https://www.bdbiosciences.com/en-eu/products/reagents/flow-cytometry-reagents/research-reagents/singlecolor-antibodies-ruo/apc-cy-7-mouse-anti-human-cd11b.557754?tab=product_details
- CD16 (3G8): https://www.bdbiosciences.com/en-eu/products/reagents/flow-cytometry-reagents/research-reagents/single-colorantibodies-ruo/bv421-mouse-anti-human-cd16.562874?tab=product_details
- CD20 (2H7): https://www.biolegend.com/fr-fr/products/brilliant-violet-570-anti-human-cd20-antibody-7456
- CD4 (L200): https://www.nhpreagents.org/ReactivityDatabase
- CD45 (Clone D058-1283): https://www.bdbiosciences.com/en-us/products/reagents/flow-cytometry-reagents/research-reagents/single-color-antibodies-ruo/bv786-mouse-anti-nhp-cd45.563861?tab=product_details
- CD28 (CD28.2): https://www.bdbiosciences.com/en-us/products/reagents/flow-cytometry-reagents/research-reagents/singlecolor-antibodies-ruo/buv496-mouse-anti-human-cd28.741168?tab=product_details
- CD49d (9F10): https://www.nhpreagents.org/ReactivityDatabase
- HLA-DR (G-46-6): https://www.bdbiosciences.com/en-us/products/reagents/flow-cytometry-reagents/research-reagents/singlecolor-antibodies-ruo/buv661-mouse-anti-human-hla-dr.612980?tab=product_details
- CD11c (B-Ly6): https://www.nhpreagents.org/ReactivityDatabase
- CD123 (7G3): https://www.nhpreagents.org/ReactivityDatabase
- CD8 (SK1): https://www.bdbiosciences.com/en-us/products/reagents/flow-cytometry-reagents/research-reagents/single-

colorantibodies-
ruo/buv805-mouse-anti-human-cd8.612889?tab=product_details
- CD66 (130-116-522): https://www.miltenyibiotec.com/US-en/products/cd66abce-antibody-anti-humantet2.html#conjugate=fitc:size=100-tests-in-200-ul
- CD162 (KPL-1): https://www.nhpreagents.org/ReactivityDatabase
- CD3 (SP34-2): https://www.bdbiosciences.com/en-us/products/reagents/flow-cytometry-reagents/research-reagents/single-colorantibodies-
ruo/alexa-fluor-700-mouse-anti-human-cd3.557917?tab=product_details
- CD20 (2H7): https://www.bdbiosciences.com/en-us/products/reagents/flow-cytometry-reagents/research-reagents/single-colorantibodies-
ruo/pe-cf594-mouse-anti-human-cd20.562295
- CD11b (ICRF44): https://www.thermofisher.com/us/en/home/life-science/cell-analysis/cell-analysis-learning-center/cell-analysisresource-
library/ebioscience-resources/human-antibody-cross-reactivity-chart.html
- CD11c (3.9): https://www.bdbiosciences.com/en-us/products/reagents/flow-cytometry-reagents/research-reagents/single-colorantibodies-
ruo/apc-r700-mouse-anti-human-cd11c.566610?tab=product_details
- CD8 (RPA-T8): https://www.thermofisher.com/us/en/home/life-science/cell-analysis/cell-analysis-learning-center/cell-analysisresource-
library/ebioscience-resources/human-antibody-cross-reactivity-chart.html
- CD206 (19.2): https://www.nhpreagents.org/ReactivityDatabase
- CD163 (GHI/61): https://www.nhpreagents.org/ReactivityDatabase
- HLA-DR (G46-6): https://www.fishersci.com/shop/products/hla-dr-mouse-anti-human-rhesus-cynomolgus-baboon-r718-cloneg46-6-also-known-as-l243-bd-horizon/p-7227060
- CD64 (10.1): https://www.nhpreagents.org/ReactivityDatabase
- IL-8 (G265-8): https://www.nhpreagents.org/ReactivityDatabase
- TNF- (mAB11): https://www.biolegend.com/en-gb/products/brilliant-violet-605-anti-human-tnf-alpha-antibody-7679?GroupID=GROUP24
- IL-10 (JES3-9D7): https://www.nhpreagents.org/ReactivityDatabase

Eukaryotic cell lines

Policy information about cell lines and Sex and Gender in Research

Cell line source(s)	HEK293T , THP-1, Jurkat- T cell lines from the NCI .<br>729.6 lymphoblastoid B-cell lines kindly provided by Patrick Green, Department of Veterinary Biosciences, Ohio State University.
Authentication	The cells were not authenticated
Mycoplasma contamination	cells were not tested for mycoplasma contamination
Commonly misidentified lines<br>(See ICLAC register)	The cell lines used in the study are not listed in the recent Cross-Contaminations_v13_distribution list. Those cells were not authenticated.

Animals and other research organisms

Policy information about studies involving animals; ARRIVE guidelines recommended for reporting animal research, and Sex and Gender in Research

Laboratory animals	*For laboratory animals, report species, strain and age OR state that the study did not involve laboratory animals.*
Wild animals	All animals used in this study were Indian rhesus macaques (Macaca mulatta) obtained from the free-range colony on Morgan Island (South Carolina) or Covance Research Products (Princeton, NJ). The macaques aged between 5 and 8 years at the initiation of the study
Reporting on sex	Male and female rhesus macaques were used in this study.
Field-collected samples	n/a
Ethics oversight	Animals were housed and maintained at the NCI Animal Facility at the NIH, Bethesda, MD. The NIH is accredited by AAALAC International and follows the Public Health Service Policy for the Care and Use of Laboratory Animals. Animal care was provided in accordance with the procedures outlined in the 'Guide for Care and Use of Laboratory Animals' (National Research Council; 2011; National Academy Press; Washington, D.C.). Animals were handled in accordance with AAALAC standards in an AAALAC-accredited facility (OLAW, Animal Welfare Assurance A4149-01 for NIH). All animal care and procedures were carried out under protocols approved by the NCI and/or NIAID Animal Care and Use Committees before study initiation (ACUC; Protocol VB-043). Animals were closely monitored daily for any signs of illness, and appropriate medical care was provided as needed. Animals were socially housed per the approved ACUC protocol and social compatibility. All clinical procedures, including biopsy collection, administration of anesthetics and analgesics, and euthanasia, were carried out under the direction of a laboratory animal veterinarian. Steps were taken to ensure the welfare of the animals and minimize discomfort of all animals used in this study. Animals were fed daily with a

fresh diet of primate biscuits, fruit, peanuts, and other food items to maintain body weight or normal growth. Animals were monitored for mental health and provided with physical enrichment including sanitized toys, destructible enrichment (cardboard and other paper products), and audio and visual stimulation.

Note that full information on the approval of the study protocol must also be provided in the manuscript.

Plants

Seed stocks	n/a
Novel plant genotypes	n/a
Authentication	n/a

Flow Cytometry

Plots

Confirm that:

- [x] The axis labels state the marker and fluorochrome used (e.g. CD4-FITC).
- [x] The axis scales are clearly visible. Include numbers along axes only for bottom left plot of group (a 'group' is an analysis of identical markers).
- [x] All plots are contour plots with outliers or pseudocolor plots.
- [x] A numerical value for number of cells or percentage (with statistics) is provided.

Methodology

Sample preparation

For the whole blood cell phenotyping, 100µl of fresh EDTA whole blood were stained with Fluorochrome-conjugated mAbs. 1ul of the Following antibodies were used: FITC anti-CD8 (clone DK25; cat. #FCMAB176F; EMB Millipore Corp.), BB700 anti-CD14 (clone M5E2; cat. #745790; BD Biosciences), PE-Cy5 anti-CD95 (clone DX2; cat. #305610; BioLegend), PE-Cy7 anti CD159 (NKG2a) (clone Z199; cat. #B10246; Beckman Coulter), APC anti-CD66abce (clone TET2; cat. #130-118-539; Miltenyi Biotec), Alexa 700 anti-CD3 (clone SP34-2; cat. #557917; BD Biosciences), APC-Cy7 anti-CD11b (clone ICRF44; cat. #557754; BD Biosciences), BV421 anti-CD16 (clone 3G8; cat. #562874; BD Biosciences), BV570 anti-CD20 (clone 2H7; cat. #302332; BioLegend), BV750 anti-CD4 (clone L200; cat. #747202; BD Biosciences), BV786 anti-CD45 (clone D058-1283; cat. #563861; BD Biosciences), BUV496 anti-CD28 (clone CD28.2; cat. #741168; BD Biosciences), BUV563 anti-CD49d (clone 9F10; cat. #749455; BD Biosciences), BUV661 anti-HLA-DR (clone G-46-6; cat. #612980; BD Biosciences), BV711 anti-CD11c (clone B-ly6; cat. #741139; BD Biosciences), BV650 anti-CD123 (clone 7G3; cat. #572392; BD Biosciences), BUV805 anti-CD8 (clone SK1; cat. #612889; BD Biosciences). Blue LIVE/Dead viability dye (cat. #L23105; Thermo Fisher Scientific, Waltham, MA) was used to exclude dead cells.

Briefly, following the 30 minutes staining at RT the red blood cells were lysed by incubating the samples with the BD FACS Lysing solution (cat. #349202 BD Biosciences, San Jose, CA) for 10 min at RT. Samples were washed with PBS and resuspended in 1% ultrapure formaldehyde (cat. #1008B-10 Tousimis, Rockville, MD). Flow cytometry acquisitions were performed on a FACSymphony A5 and examined using FACSDiva software (BD Biosciences) by acquiring all stained cells. Data was further analyzed using FlowJo v10.1 (TreeStar, Inc., Ashland, OR).

To measure neutrophils, monocytes, myeloid dendritic cells (mDC) and plasmacytoid dendritic cells (pDC) in bronchoalveolar lavage (BAL) and whole blood of the animals, 200ul of whole EDTA blood and 1x106 cells freshly isolated from BAL were stained with Blue LIVE/DEAD viability dye (cat. #L34962, Thermo Fisher Scientific) to exclude dead cells. 5ul of the following antibodies were used for cell surface staining: FITC anti-CD66abce (clone TET2; cat. #130-116-522; Miltenyi Biotec), BB700 anti-CD162 (clone KPL-1; cat. #745768; BD Biosciences), Alexa 700 anti-CD3 (clone SP34-2; cat. #557917; BD Biosciences), Alexa 700 anti-CD20 (clone 2H7; cat. #560631; BD Biosciences), APC-Cy7 anti-CD11b (clone ICRF44; cat. #47-0118-42; Invitrogen™), BV480 anti-CD11c (clone 3.9; cat. #748269; BD Biosciences), BV650 anti-CD8 (clone RPA-T8; cat. #563821; BD Biosciences), BV750 anti-CD206 (clone 19.2; cat. #746891; BD Biosciences), BV786 anti-CD45 (clone D058-1283; cat. #563861; BD Biosciences), BUV395 anti-123 (clone 7G3; cat. #564195; BD Biosciences), BUV496 anti-CD16 (clone 3G8; cat. #612944; BD Biosciences), BUV563 anti-CD163 (clone GH1/61; cat. #741402; BD Biosciences), BUV661 anti-HLA-DR (clone G46-6; cat. #612980; BD Biosciences), BUV737 anti-CD64 (clone 10.1; cat. #564426; BD Biosciences), BUV805 anti-CD14 (clone M5E2; cat. #612902; BD Biosciences). Subsequently cells were permeabilized with Foxp3 / Transcription Factor Staining Buffer Set (Invitrogen, cat. #00-5523-00) according to manufacturer recommendation. The following antibodies were used for intracellular staining, PE anti-MPO (clone MPO455-8E6; cat. #12-1299-42; Invitrogen™), BV421 anti-IL-8 (clone G265-8; cat. #563310; BD Biosciences), BV605 anti-TNF-α (clone mAB11; cat. #502936; BioLegend) and BV711 anti-IL-10 (clone JES3-9D7; cat. #564050; BD Biosciences). Samples were washed with PBS and resuspended in 1% ultrapure formaldehyde (cat. #1008B-10 Tousimis, Rockville, MD). Flow cytometry acquisitions were performed on a FACSymphony A5 and examined using FACSDiva software (BD Biosciences) by acquiring all stained cells. Data was further analyzed using FlowJo v10.1 (TreeStar, Inc., Ashland, OR).

Instrument	FACSymphony A5
Software	Acquisition was done using FACSDiva software (BD Biosciences); and cytometry data were analyzed using FlowJo LLC 10.10.0 (TreeStar, Inc.)
Cell population abundance	No cell sorting experiment was performed.
Gating strategy	1- T cell lineages were identified following the gating strategy; i) Singlets/Live/CD45+/CD20-CD14-/CD3+CD4+ for CD4+ T cells, ii) CD4+CD8-/CD95+ for CD4+ memory helper T cells, iii) CD4+CD95- for CD4+ naïve helper T cells, iv) CD4+CD95-CD28- for CD4+ effector memory helper T cells, v) CD4+CD95-CD28+ for CD4+ central memory helper T-cells, vi) CD3+CD4-CD8+ for CD8+ cytotoxic T cells (CTLs), vii) CD3+CD8+CD95+ for memory CTLs, viii) CD8+CD95- for naïve CTLs, ix) CD8+CD95+CD28- for effector memory CTLs, x) CD8+CD95+-CD28+ for central memory CTLs. NK and neutrophils were identified following the gating strategies Singlets/Live/CD45+/CD3-CD20-/CD14-/CD8+NKG2a+, and Singlets/Live/CD45+/CD20-CD3-/CD8-/CD123-CD11c-/CD14-CD16-/CD66abce+ respectively. Monocyte populations were identified as Singlets/Live/CD45+/HLA-DR+CD20-/CD3-CD8- and differentiated by the expression of CD14 and CD16. Classical monocytes were identified as CD14+CD16-, intermediate monocytes as CD14+CD16+, and non-classical monocytes as CD14-CD16+.<br>2- Myeloid cells in the blood were identified following the gating strategy, i) Singlets/Live/CD45+/CD3-CD20-CD8-/CD123-CD11c-/CD14-CD16-/CD66abce+ cells for Neutrophils, ii) Singlets/Live/CD45+/CD3-CD20-CD8-/HLA-DR+/FSC-AlowSSC-Alow and differentiated by the expression of CD14 and CD16 for monocytes, classical monocytes (CD14+CD16-), Intermediate monocytes (CD14+CD16+), and non-classical as (CD14-CD16+); iii) pDC and mDc Singlets/Live/CD45+/CD3-CD20-CD8-/HLA-DR+/CD14-/CD123+CD11c- cells and Singlets/Live/CD45+/CD3-CD20-CD8-/HLA-DR+/CD14-/CD123-CD11c+ cells for pDC and mDC respectively.<br>3- Myeloid cells in the BAL were identified following the gating strategy; i) Singlets/Live/CD45+/CD206-/CD163-/CD3-CD20-CD8-/CD123-CD11c-/CD14-CD16-/CD66abce+ for Neutrophils, ii) Singlets/Live/CD45+/CD206-/CD163-/CD3-CD20-CD8-/HLA-DR+/FSC-AlowSSC-Alow and differentiated by the expression of CD14 and CD16 infiltrated monocytes, classical monocytes (CD14+CD16-), Intermediate monocytes (CD14+CD16+), and non-classical as (CD14-CD16+); (iii) Singlets/Live/CD45+/CD206-/CD163-/CD3-CD20-CD8-/HLA-DR+/CD14-/CD123+CD11c- cells and Singlets/Live/CD45+/CD3-CD20-CD8-/HLA-DR+/CD14-/CD123-CD11c+ cells for pDC and mDc respectively.<br>4- In the efferocytosis assay the following gating strategy was used: FSC/SSC/Single cells/CytoTell™ Blue+/GFP+.

☒ Tick this box to confirm that a figure exemplifying the gating strategy is provided in the Supplementary Information.
