# Supplementary material for: Author Correction: High expression of Rex-orf-I and HBZ mRNAs and bronchiectasis in lung of HTLV-1A/C infected macaques
Source: Nat Commun. 2026 Sep 3;17:9455. doi: 10.1038/s41467-026-77329-y (PMC13542064; doi:10.1038/s41467-026-77329-y)

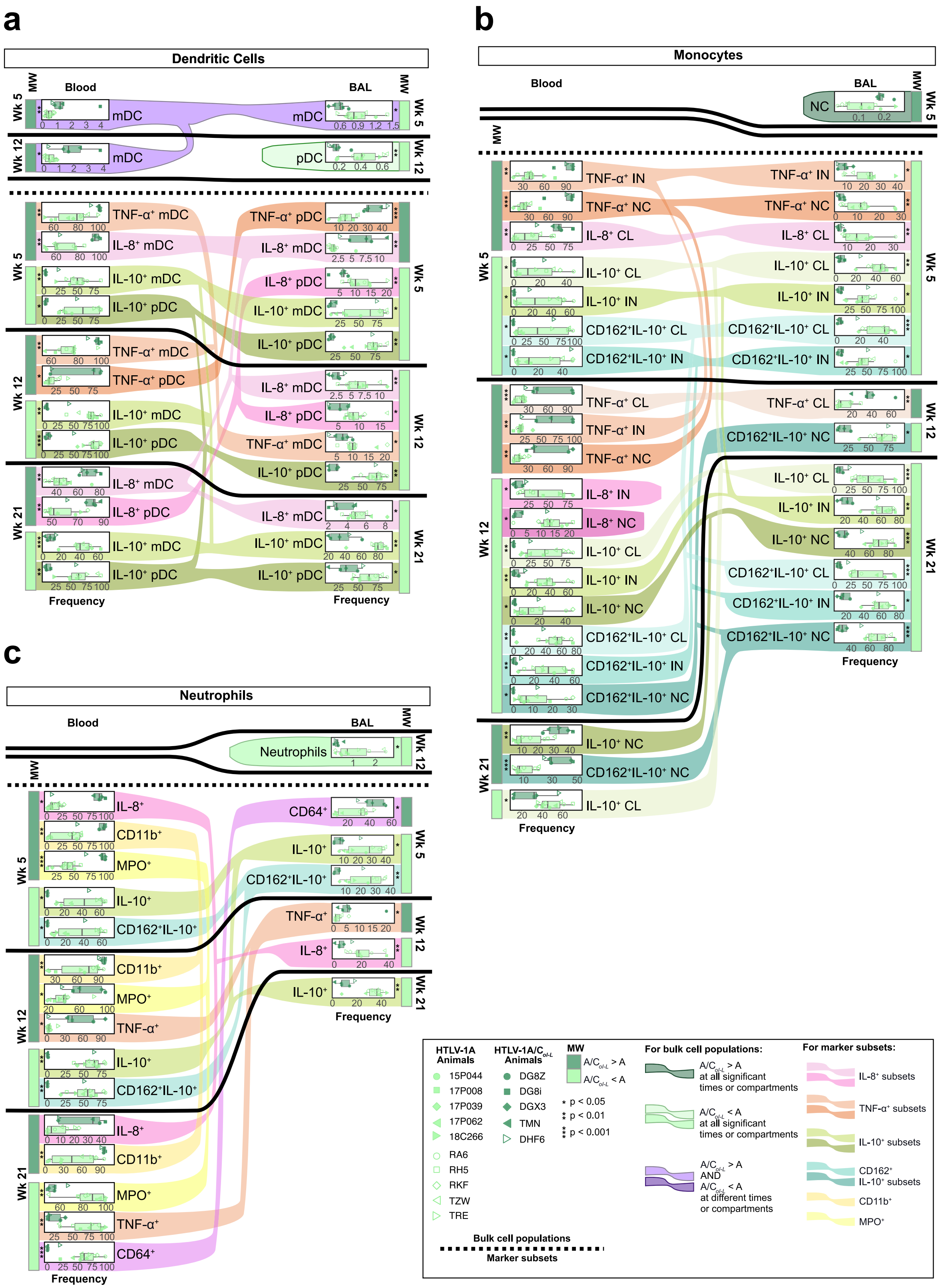
a
Dendritic Cells
Blood
BAL
MW
Wk 5
Wk 12
Wk 21
mDC
pDC
TNF-α⁺ mDC
IL-8⁺ mDC
IL-10⁺ mDC
IL-10⁺ pDC
TNF-α⁺ pDC
IL-8⁺ pDC
Frequency
b
Monocytes
NC
TNF-α⁺ IN
TNF-α⁺ NC
IL-8⁺ CL
IL-10⁺ CL
IL-10⁺ IN
CD162⁺IL-10⁺ CL
CD162⁺IL-10⁺ IN
TNF-α⁺ CL
CD162⁺IL-10⁺ NC
IL-8⁺ IN
IL-8⁺ NC
IL-10⁺ NC
c
Neutrophils
IL-8⁺
CD11b⁺
MPO⁺
IL-10⁺
CD162⁺IL-10⁺
TNF-α⁺
CD64⁺
HTLV-1A Animals
HTLV-1A/C_{orf-I} Animals
15P044
17P008
17P039
17P062
18C266
RA6
RH5
RKF
TZW
TRE
DG8Z
DG8i
DGX3
TMN
DHF6
A/C_{orf-I} > A
A/C_{orf-I} < A
* p < 0.05
** p < 0.01
*** p < 0.001
For bulk cell populations:
A/C_{orf-I} > A at all significant times or compartments
A/C_{orf-I} < A at all significant times or compartments
A/C_{orf-I} > A AND A/C_{orf-I} < A at different times or compartments
For marker subsets:
IL-8⁺ subsets
TNF-α⁺ subsets
IL-10⁺ subsets
CD162⁺ IL-10⁺ subsets
CD11b⁺
MPO⁺
Bulk cell populations
Marker subsets

Supplementary Figure 6

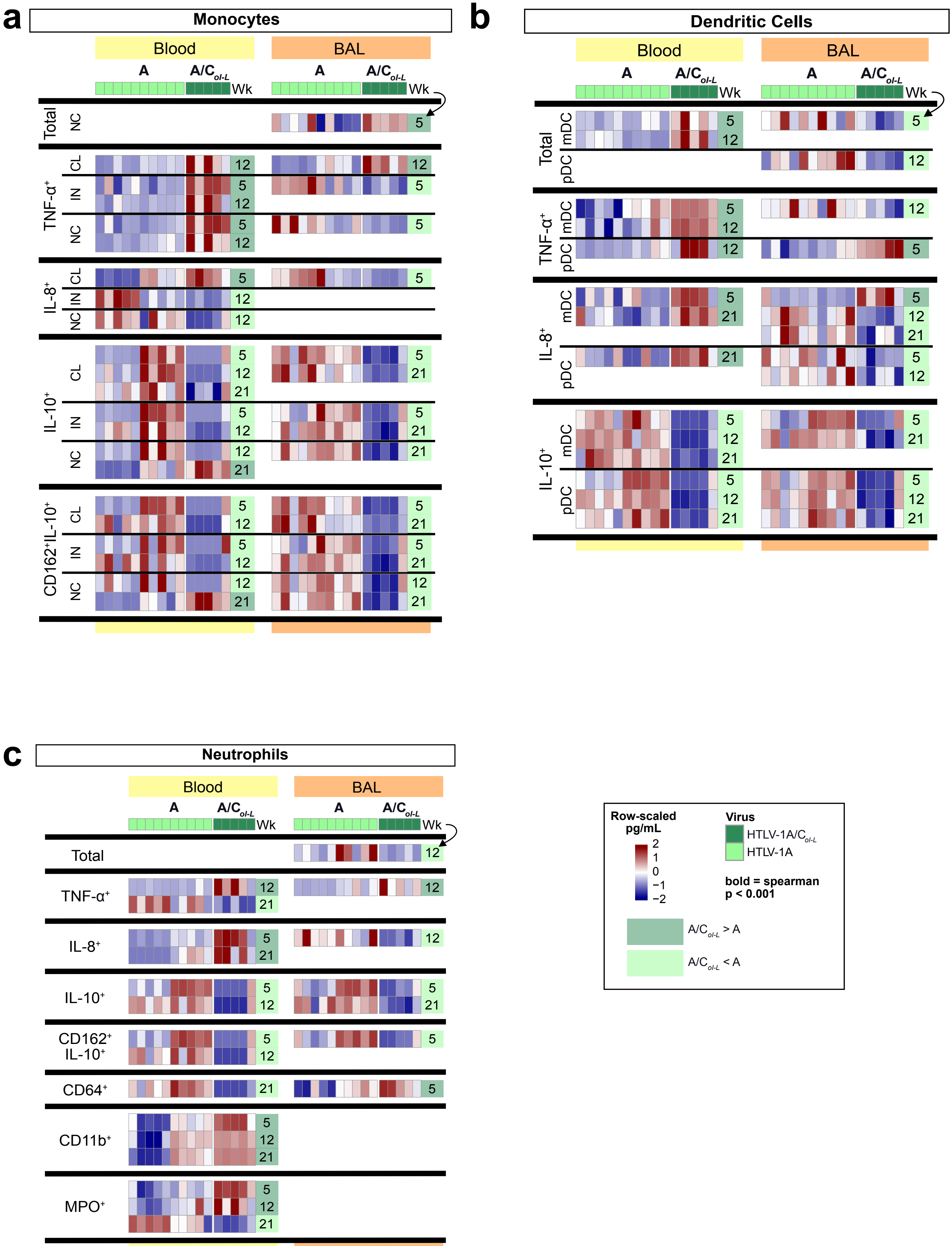

Supplementary Figure 7

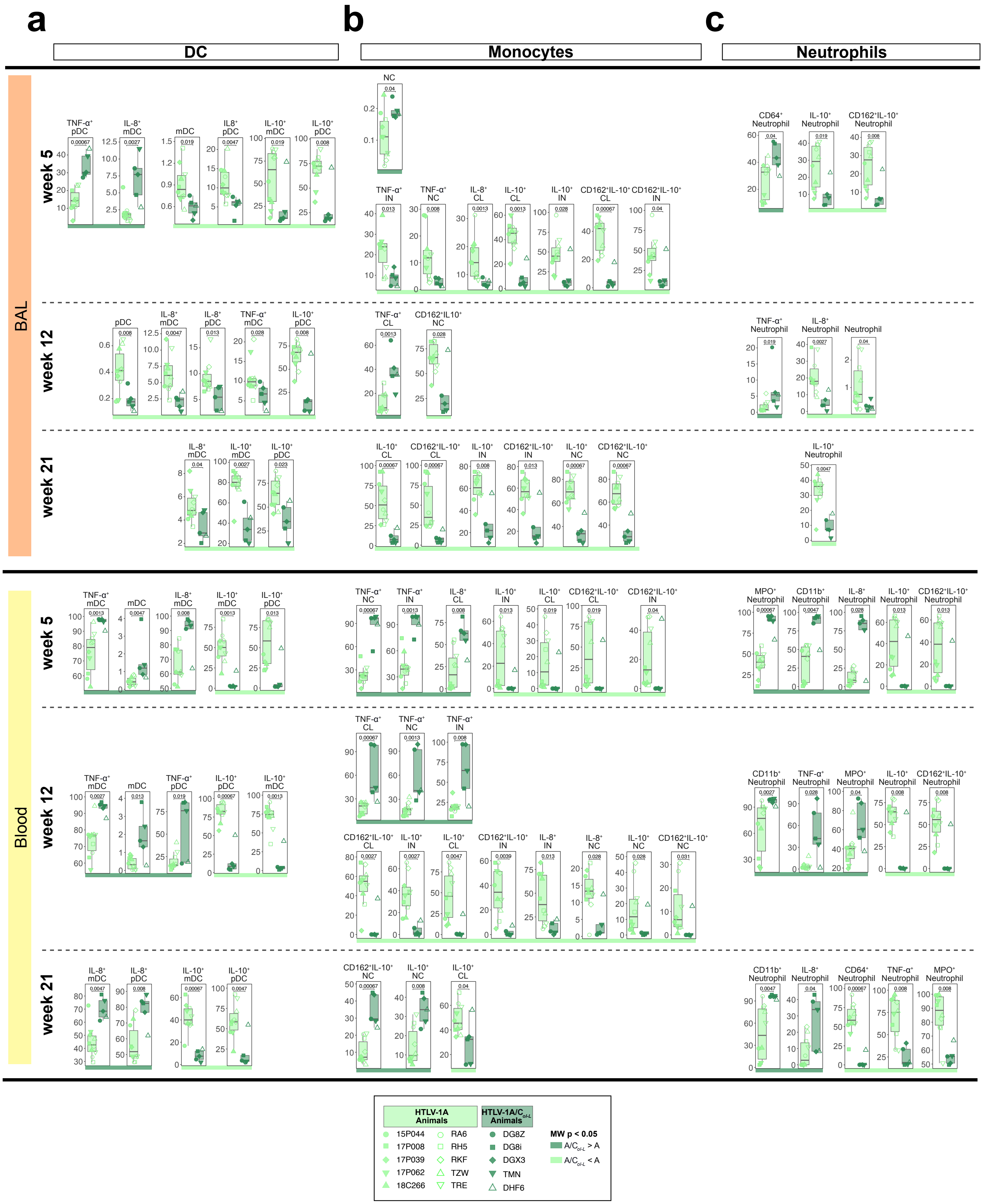

Supplement: Supplementary file 1 [file 41467_2026_77329_MOESM1_ESM.pdf]
